# Cytotoxic Phenanthrene, Dihydrophenanthrene, and Dihydrostilbene Derivatives and Other Aromatic Compounds from *Combretum laxum*

**DOI:** 10.3390/molecules25143154

**Published:** 2020-07-10

**Authors:** Eder Bisoli, Talita Vilalva Freire, Nídia Cristiane Yoshida, Walmir Silva Garcez, Lyara Meira Marinho Queiróz, Maria de Fátima Cepa Matos, Renata Trentin Perdomo, Fernanda Rodrigues Garcez

**Affiliations:** 1 Institute of Chemistry, Universidade Federal de Mato Grosso do Sul, Campo Grande 79074-460, MS, Brazil; ederbisoli@yahoo.com.br (E.B.); talita.vilalva@gmail.com (T.V.F.); nidiayoshida@gmail.com (N.C.Y.); walmir.garcez@ufms.br (W.S.G.); 2 Laboratory of Molecular Biology and Cell Culture, School of Pharmaceutical Sciences, Food Technology, and Nutrition, Universidade Federal de Mato Grosso do Sul, Campo Grande 79070-900, MS, Brazil; lyarameira@hotmail.com (L.M.M.Q.); matosmfc@gmail.com (M.d.F.C.M.); renataperdomo@gmail.com (R.T.P.)

**Keywords:** combretaceae, cytotoxic activity, bibenzyl, phenanthrene, dihydrophenanthrene, antioxidant activity

## Abstract

The chemical investigation of the roots and stems of *Combretum laxum* yielded a new dihydrostilbene derivative, 4′-hydroxy-3,3′,4-trimethoxy-5-(3,4,5-trimethoxyphenoxy)-bibenzyl (**1**), two phenanthrenes (**2**–**3**), and three dihydrophenanthrenes (**4**–**6**), along with one lignan, three triterpenoids, one aurone, one flavone, one naphthoquinone, and two benzoic acid derivatives. Their structures were determined by 1D and 2D nuclear magnetic resonance (NMR) spectroscopic techniques and/or mass spectrometry data. The occurrence of dihydrostilbenoid, phenanthrene and dihydrophenanthrene derivatives is unprecedented in a *Combretum* species native to the American continent. 2,7-Dihydroxy-4,6-dimethoxyphenanthrene, 2,6-dihydroxy-4,7-dimethoxy-9,10-dihydrophenanthrene and 5-*O*-methyl apigenin are novel findings in the Combretaceae, as is the isolation of compounds belonging to the chemical classes of aurones and naphthoquinones, while (+)-syringaresinol is reported for the first time in the genus *Combretum*. Compounds **1**–**6** were also evaluated for their in vitro cytotoxicity against five human cancer cell lines, and radical-scavenging ability against 1,1-diphenyl-2-picryl-hydrazyl (DPPH). 6-Methoxycoelonin (**4**) was the most cytotoxic against melanoma cells (IC_50_ 2.59 ± 0.11 µM), with a high selectivity index compared with its toxicity against nontumor mammalian cells (SI 25.1). Callosin (**6**), despite exhibiting the strongest DPPH-scavenging activity (IC_50_ 17.7 ± 0.3 µM), proved marginally inhibitory to the five cancer cell lines tested, indicating that, at least for these cells, antioxidant potential is unrelated to antiproliferative activity.

## 1. Introduction

The genus *Combretum*, found in tropical and subtropical areas, is the largest within the Combretaceae and most of its species are extensively used in the folk medicine of African and Asian countries for the treatment of a wide variety of health disorders [1,2,3]. Amongst the approximately 20 genera comprising the Combretaceae, the genus *Combretum* is notable for providing a number of classes of biologically active chemical constituents. Among these, typical examples are the combretastatins and their analogues—stilbenoid derivatives which are included in the group of the most potent antineoplastic agents of natural origin [4,5,6,7].

*Combretum laxum* Jacq., popularly known as “pombeiro branco”, is a woody shrub distributed in different habitats throughout Brazil, and in Mato Grosso do Sul state (Midwest Brazil) is part of the Pantanal biome flora. In the course of the chemical investigation on Brazilian plants from the Pantanal, we previously reported the isolation of 11 triterpenoid derivatives from the stems of *C. laxum* [8]. In the present study, we describe the isolation of the new dihydrostilbenoid 4′-hydroxy-3,3′,4-trimethoxy-5-(3,4,5-trimethoxyphenoxy)-bibenzyl (**1**), the phenanthrenes 2,7-dihydroxy-4,6-dimethoxyphenanthrene (**2**) and 2,6-dihydroxy-3,4,7-trimethoxyphenanthrene (**3**), the dihydrophenanthrenes 6-methoxycoelonin (**4**), 2,6-dihydroxy-3,4,7-trimethoxy-9,10-dihydrophenanthrene (**5**), and callosin (**6**), and the lignan syringaresinol (**7**) from its roots, in addition to the triterpenoids arjunolic (13), betulinic (14) and maslinic (**15**) acids—two of which were already described in our former study on the stems of *C. laxum*. Further investigation of the stems afforded the aurone sulfuretin (**8**), the flavone 5-*O*-methyl apigenin (**9**), the naphthoquinone lapachol (**10**), and the benzoic acid derivatives 3,4-dimethoxybenzoic (**11**) and 3-hydroxy-4-methoxybenzoic acids (**12**). Compounds **2**, **6** and **9** are being reported for the first time in the Combretaceae, as well as **7** in the genus *Combretum*, while this is the second reported occurrence of **3** and **4** in this genus. This is also the first time that compounds belonging to the chemical classes of aurones and naphthoquinones are being described in a member from the Combretaceae. The cytotoxic potential of compounds **1**–**6** against five human cancer cell lines, namely MCF-7, 786-0, UACC-62, NCI/ADR-RES, and Hep2, as well as their free-radical scavenging ability were also evaluated in this work.

## 2. Results and Discussion

### 2.1. Extraction, Isolation, and NMR Spectroscopic Data

**Figure 1:**
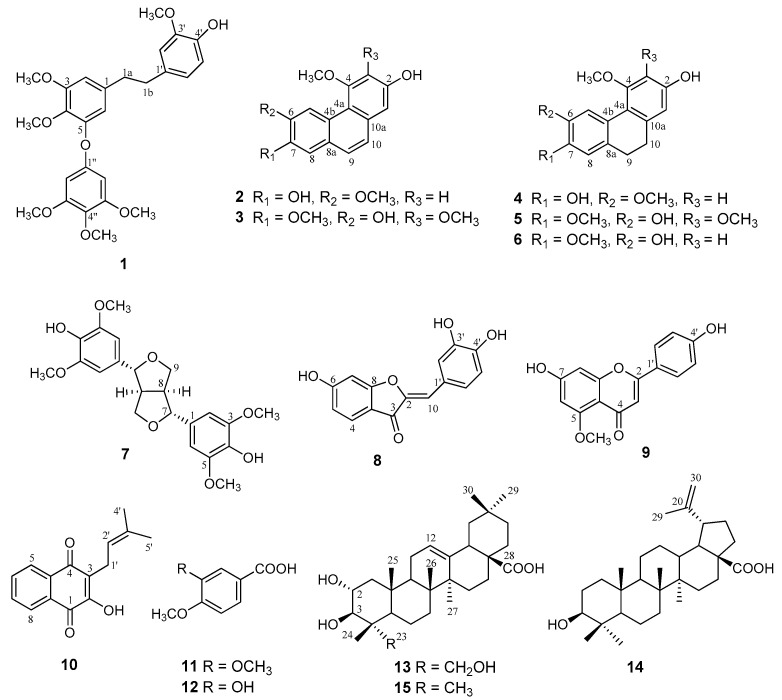
Structures of compounds (**1**–**15**) isolated from the roots and stems of*Combretum laxum*.

After a combination of column chromatography on silica gel, gel filtration on Sephadex LH-20 and reversed-phase HPLC separations of the CH_2_Cl_2_ phase resulting from partitioning of the EtOH extract from the roots of *C. laxum*, compounds **1**–**7**, and **13**–**15**, comprising phenanthrenes, dihydrophenanthrenes, dihydrostilbene, lignan, and triterpenes (Figure 1), were obtained.

Compound **1** was isolated as a yellow powder. Its molecular formula was determined to be C_26_H_30_O_8_, as revealed from its HRESIMS (high resolution electrospray ionization mass spectrometry) data (*m*/*z* 509.1588 [M + K]^+^) data (Figure S1). The aromatic nature of **1** was deduced by the presence of 16 carbon signals observed in the ^13^C NMR spectrum as seven methines, ranging from δ_C_ 93 to 122, and 11 quaternary carbons (including eight oxygen-bearing carbons) found between δ_C_ 132 to 135, as well as by proton resonances in the region of δ_H_ 6.08 to 6.68 (Table 1) [9]. These spectra also showed signals for six aromatic methoxy groups, wherein two were shown to be sterically hindered, as revealed by their chemical shifts at δ_C_ 61.0 and 61.3 [9]. In the ^1^H nuclear magnetic resonance (NMR) spectrum, two multiplets assignable to two pairs of methylene benzylic protons were observed at δ_H_ 2.74 and 2.76 [9,10], which in turn showed correlations in the HSQC (heteronuclear single quantum coherence) spectrum with the carbon signals at δ_C_ 39.4 and 38.6, respectively. These data, together with long-range HMBC (heteronuclear multiple bond correlation) correlations between the foregoing protons and benzene ring carbons at δ_C_ 134.7 and 139.3, respectively, were in accordance with the presence of a dihydrostilbene moiety in the structure of **1** [10,11]. Further information given by the chemical shifts and splitting patterns of the signals of the aromatic protons indicated that one benzene ring of the bibenzyl unit (ring A) was 3,4,5-trioxygenated, as revealed by a pair of *meta*-coupled protons at δ_H_ 6.25 and 6.30 (*J* = 3.0 Hz each) [9,10], which, in turn, showed cross-peak correlations in the HSQC spectrum with the carbon signals at δ_C_ 105.0 and 110.3, respectively. Three protons displayed as an ABC set at δ_H_ 6.64 (d, *J* = 2.0 Hz), 6.68 (d, *J* = 9.0 Hz), and 6.60 (dd, *J* = 9.0, 2.0 Hz) supported a 3,4-dioxygenated substitution pattern for the other benzene ring (ring B) in the structure of **1** [9,10]. The remaining signals observed in the ^1^H and ^13^C NMR spectra of **1** were ascribable to a 1,3,4,5-tetraoxygenated benzene ring, as shown by the two-proton singlet at δ_H_ 6.08 and carbon resonances at δ_C_ 155.4 (C), 94.0 (2 × CH), 155.0 (2 × C). Compound **1** was thus assumed to be an oxygenated dihydrostilbene derivative, bearing an additional 3,4,5-trioxygenated phenoxy substituent. The HMBC experiments allowed the positions of the oxygenated functions in the benzene rings A and B of the dihydrostilbene moiety, as well as in the trioxygenated phenoxy substituent to be ascertained. Accordingly, the signal at δ_H_ 3.77 related to one of the aromatic methoxy groups showed a three-bond proton-carbon correlation with the carbon signal at δ_C_ 148.7 in the HMBC spectrum. A prominent cross-peak between the latter and the doublet at δ_H_ 6.68 (H-5′, ^3^*J*), as well as a two-bond correlation with the doublet at δ_H_ 6.64, established the location of the OCH_3_ group in the 3,4-dioxygenated B ring at C-3′. No correlations were found between the other methoxy protons and the carbon resonances assigned to the B ring, thus supporting the placement of a hydroxy group at C-4′ (δ_C_ 145.6). Long-range connectivities between C-4′ and H-2′, H-5′ and H-6′ corroborated these assignments. Other correlations discernible in the HSQC and HMBC spectra allowed the positions of the methoxy groups at δ_H_ 3.75/δ_C_ 56.3 and δ_H_ 3.73/δ_C_ 61.0 to be established at C-3 and C-4 of the A ring, respectively, as well as the linkage site of the trimethoxylated phenoxy substituent at C-5. Particularly, the signal at δ_H_ 6.25 related to H-2 showed two- and three-bond correlations with the carbon signals at δ_C_ 154.2 and 135.8, which were thus assigned to C-3 and C-4, respectively. The latter also displayed a three-bond-correlation with H-6 (δ_H_ 6.30). The HMBC spectrum also exhibited a two-bond correlation between H-6 and the carbon signal at δ_C_ 151.2 attributed to C-5, whose chemical shift was in accordance with that of an aromatic carbon linked to a phenoxy substituent. From the foregoing data, the structure of compound **1** was deduced to be 4′-hydroxy-3,3′,4-trimethoxy-5-(3,4,5-trimethoxyphenoxy)-bibenzyl, hitherto unreported in the literature.

The molecular formula of compound **2** was deduced as C_16_H_14_O_4_ based on the [M + H]^+^ ion at *m*/*z* 271.0963 in the HRESIMS (Figure S8), indicating 10 degrees of unsaturation. The 1D-NMR data of **2** clearly revealed its aromatic nature and supported the presence of eight fully substituted carbons (four of which oxygenated), and six methine carbons, in addition to two aromatic methoxy groups, evidenced at δ_H_ 4.02/δ_C_ 56.2 and δ_H_ 4.12/δ_C_ 56.0 (Table 2). The ^1^H NMR spectrum of **2** showed a pair of *meta*-coupled protons in a 1,2,3,5-tetrasubstituted benzene ring, at δ_H_ 6.79 and 6.89 (*J* = 3.0 Hz each), two isolated protons at δ_H_ 7.24 (s) and 9.11 (s), and a pair of *ortho*-coupled protons at δ_H_ 7.44 and 7.56 (d, *J* = 9.0 Hz each). The chemical shifts and splitting patterns of these last two protons were shown to be characteristic of H-9 and H-10 of a phenanthrene derivative [12], which in turn showed cross-peak correlations in the HSQC spectrum with carbon resonances at δ_C_ 127.9 and 125.4, respectively. Therefore, resonances of the doublets at δ_H_ 6.79 and 6.89 corresponding to the *meta*-coupled protons, and that of the one-proton singlet at δ_H_ 9.11, together with their respective ^1^H-^13^C connectivities detectable in the HSQC spectrum, implied that the structure of **2** comprised a 2,4,6,7-tetraoxygenated phenanthrene skeleton. Considering that only two methoxy groups were identified in the NMR spectra, the remaining oxygenated functions in the structure of **2** must be attributed to the presence of two hydroxyls. The signals at δ_H_ 7.24 and 9.11 were thus ascribed to H-8 and the anisotropically deshielded H-5, respectively [13], which depicted correlations with the corresponding carbon resonances at δ_C_ 112.2 and 109.6 in the HSQC spectrum. HMBC long-range connectivities from H-5 and H-8 to C-6 (δ_C_ 148.3, ^2^*J* and ^3^*J*, respectively), C-7 (δ_C_ 145.8, ^3^*J* and ^2^*J*, respectively), and C-4b (δ_C_ 125.5, ^2^*J* and ^3^*J*, respectively), in addition to ^3^*J* couplings between H-5 and C-4a (δ_C_ 115.5) and C-8a (δ_C_ 128.2), and between H-8 and C-9 (δ_C_ 127.9) were consistent with the foregoing assignments. The relative positions of the hydroxy and methoxy functionalities in **2** were ascertained from key nuclear Overhauser effect (NOE) relationships found between the methoxy at δ_H_ 4.12 and H-3 (d, δ_H_ 6.79), and between the methoxy at δ_H_ 4.02 and H-5 (δ_H_ 9.11), thus indicating the location of these OCH_3_ groups at C-4 and C-6, respectively. The linkage sites of the hydroxy functions were therefore determined at C-2 and C-7. Compound **2** was thus shown to be 2,7-dihydroxy-4,6-dimethoxyphenanthrene, whose spectroscopic data agreed with those reported for this phenanthrene obtained from *Bulbophyllum vaginatum* (Orchidaceae) [14], which is thus being described for the first time in the Combretaceae.

The molecular formula of **3** was established as C_17_H_16_O_5_, as deduced by an [M + H]^+^ ion at *m*/*z* 301.1076 in the HRESIMS (Figure S15). This data, together with analysis of the ^1^H and ^13^C NMR spectra of **3**, revealed their high similarity to those of **2** (Table 2). However, the spectrometric data of **3** indicated the presence of five oxygenated substituents (three of which as methoxy groups), instead of four as in compound **2**, located at carbons C-2, C-3, C-4, C-6, and C-7 in the phenanthrene skeleton. The foregoing information were supported by a singlet at δ_H_ 7.04 ascribable to H-1, which showed a connectivity with the carbon signal at δ_C_ 109.8 in the HSQC spectrum, which in turn displayed a three-bond correlation with H-10 (δ_H_ 7.33, d, *J* = 9.0 Hz) in the HMBC spectrum. Likewise, long-range connectivities were observed between H-1 and C-2 (δ_C_ 150.4, ^2^*J*), C-3 (δ_C_ 142.9, ^3^*J*), C-10 (δ_C_ 124.9, ^3^*J*), and C-4a (δ_C_ 118.9, ^3^*J*). Since the chemical shifts of two of the methoxy carbons (δ_C_ 60.5 and 61.5) revealed their sterically hindered nature, they were placed at C-3 and C-4 positions. Three-bond correlations between the methoxyl protons at δ_H_ 4.00, 3.98, and 3.99 and C-3, C-4 (δ_C_ 152.8), and C-7 (δ_C_ 148.4), respectively, determined the attachment of the methoxy groups to these corresponding aromatic carbons, therefore establishing the location of the hydroxy functions at C-2 (δ_C_ 150.4) and C-6 (δ_C_ 147.4). Further correlations discernible in the HMBC spectrum between H-5 (δ_H_ 8.90) and both C-6 (δ_C_ 147.4, ^2^*J*) and C-7 (δ_C_ 148.4, ^3^*J*), H-8 (δ_h_ 7.27) and both C-6 (^3^*J*) and C-7 (^2^*J*), and H-9 (δ_H_ 7.50) and C-8 (δ_C_ 109.6, ^3^*J*), together with NOE-cross peaks observed between H-8 and OCH_3_ at δ_H_ 3.99, and between H-5 and OCH_3_ at δ_H_ 3.98, reinforced these assignments. The foregoing data could be satisfactorily assembled to establish the structure of **3** as 2,6-dihydroxy-3,4,7-trimethoxyphenanthrene, formerly obtained as a plant constituent only from *Combretum apiculatum*, but with no full description of its ^1^H and ^13^C NMR data [15], which are being reported herein for the first time.

Compound **4** had the molecular formula of C_16_H_16_O_4_ (nine degrees of unsaturation), as determined by HRESIMS (*m*/*z* 273.1118, [M + H]^+^) [Figure S21]. Its ^1^H and ^13^C NMR data closely resembled those of **2** (Table 2), except for the lack of signals at δ_H_ 7.56 and 7.44 assigned to H-9 and H-10, respectively, in the phenanthrene skeleton of **2**, and the presence instead of two-proton multiplets at δ_H_ 2.58 and 2.60 attributable to benzylic methylene groups of a 9,10-dihydrophenanthrene derivative [16]. Likewise, the signals at δ_C_ 127.9 (C-9) and 125.4 (C-10) in the ^13^C NMR spectrum of **2** were replaced by δ_C_ 32.1 and 30.2, respectively, in the spectrum of **4**, therefore establishing the structure of **4** as 2,7-dihydroxy-4,6-dimethoxy-9,10-dihydrophenanthrene. Additional evidence for structure **4** was provided by correlations observed in the HSQC, HMBC and NOESY spectra. The spectrometric data of **4** were in accordance with those of 6-methoxycoelonin [17], previously isolated from the orchid *Cymbidium aloifolium* [18] and further obtained from other Orchidaceae [17,19,20], in addition to *Dioscorea nipponica* (Dioscoriaceae) [21], but scarcely reported in the Combretaceae, e.g., in *Combretum hereroense* [22].

Compound **5** was assigned the molecular formula C_17_H_18_O_5_ on the basis of its HRESIMS (*m*/*z* 303.1235, [M + H]^+^) [Figure S29], with nine degrees of unsaturation. 1D- and 2D-NMR spectroscopic analysis of **5** revealed, as occurred with compounds **2** and **4**, that the structure of **5** differed from that of **3** only for the presence in the former of methylene sp^3^ carbons at C-9 and C-10 (Table 2). This assumption was confirmed by the signals at 30.4 and 31.5 in the ^13^C NMR spectrum, which showed correlations with the four-proton singlet at 2.60 in the HSQC spectrum, in addition to further information given by the 1D- and 2D-NMR spectra, including those provided by a NOESY experiment. Thus, compound **5** was shown to be 2,6-dihydroxy-3,4,7-trimethoxy-9,10-dihydrophenanthrene, a previously reported dihydrophenanthrene isolated from *Combretum molle* and *C. apiculatum* [15,23]. Its ^1^H and ^13^C NMR data are being reported for the first time.

Compound **6** had a molecular formula of C_16_H_16_O_4_ as determined from the [M + H]^+^ ion at *m*/*z* 273.1121 in the HRESIMS (Figure S37). Comparison of the 1D- and 2D-NMR spectra of **6** with those of **5** indicated their close relationship (Table 2), except for the absence of a methoxy group at C-3 in the former, as revealed by the signals of two *meta*-coupling protons at δ_H_ 6.30 (*J* = 3.0 Hz, H-1) and 6.39 (*J* = 3.0 Hz, H-3). Correlations in the HSQC spectrum between H-1/C-1 (δ_C_ 108.5) and H-3/C-3 (δ_C_ 99.3), as well as further correlations observed in the HSQC, HMBC and nuclear Overhauser effect spectroscopy (NOESY) spectra, lent support to these assignments. Compound **6** was thus identified as 2,6-dihydroxy-4,7-dimethoxy-9,10-dihydrophenanthrene, whose NMR data agreed with those reported for callosin, previously isolated only from two species of the Orchidaceae (*Agrostophyllum callosum* and *Coelogyne flaccida* [17,24]. Therefore, this is the first reported occurrence of callosin from a member of the Combretaceae.

The signals in the ^1^H NMR spectrum of compound **7** assignable to a symmetric molecule bearing two 3,5-dimethoxy-4-hydroxy substituted aromatic rings at δ_H_ 6.65 (s, 4H) and 3.84 (s, 12H), together with those belonging to a spin system at δ_H_ 4.70 (d, *J* = 3.0 Hz, 2H), 3.13 (brs, 2H), 3.70–3.80 (m, 2H), and 4.18–4.24 (m, 2H), suggested that **7** was a bistetrahydrofuran lignan. This assumption was supported by the eight signals observed in the ^13^C NMR, in which four of them were attributed to the symmetrically substituted aromatic rings, namely four methines (δ_C_ 104.5), six oxygenated carbons (δ_C_ 136.2 and 149.3), and two carbons linked to the bistetrahydrofuran moiety (δ_C_ 133.1). The remaining four signals were ascribed to the four methoxy groups at δ_C_ 56.8, and to the methine (δ_C_ 55.5 and 72.5) and methylene (δ_C_ 87.6) carbons of the foregoing bistetrahydrofuran residue. These assignments were further corroborated by HSQC and HMBC correlations, while the carbon resonances of the tetrahydrofuran rings were indicative of the pseudoequatorial orientation of the aromatic rings in the structure of **7**, as well as established their linkage to C-7/C-7′ [25]. The optical rotation value and NMR spectroscopic characteristics of **7** agreed with those of the lignan (+)-syringaresinol [26], which is being reported for the first time in the genus *Combretum*. Despite their wide distribution in plants, lignans have been scarcely found in the Combretaceae, particularly within the genus *Combretum*, with only two records in *C. fruticosum* and *C. alfredi* [27,28].

The identities of the pentacyclic triterpenes arjunolic (**13**), betulinic (**14**), and maslinic (**15**) acids were verified by comparing their NMR spectroscopic data with those of authentic samples [8,29]. Triterpenes **13** and **14** have already been isolated in our previous study on the stems of *C. laxum* [8], while maslinic acid is of common occurrence in species of the Combretaceae, including those belonging to the genus *Combretum* [30].

After partitioning of the EtOH extract from the stems of *C. laxum*, the resulting CH_2_Cl_2_ phase afforded compounds **8**–**12**—comprising an aurone, a flavone, a naphthoquinone, and two benzoic acid derivatives (Figure 1)—after fractionation procedures by silicagel and Sephadex LH-20 column chromatography, and reversed-phase high performance liquid chromatography (HPLC).

Analysis of the ^1^H NMR spectrum of compound **8** revealed the presence of two sets of signals for a total of six aromatic protons, and a vinylic singlet at δ_H_ 6.58. These data, together with 15 signals in the range of δ_C_ 99–169 in the ^13^C NMR spectrum, indicated the flavonoid nature of **8**. The oxygenation pattern of rings A and C was defined by the characteristic signals of a 6,3′,4′-trihydroxylated flavonoid [31]. The signal at δ_H_ 6.58 was assigned to a methine proton attributable to H-10 of an aurone. This assumption was confirmed by the olefinic carbon resonances at δ_C_ 112.4 (CH) and 147.2 (C), thus assigned to C-10 and C-2, respectively. These data, along with additional information provided by ^1^H-^1^H correlation spectroscopy (COSY), HSQC, and HMBC experiments, led to the identification of **8** as the aurone sulfuretin, whose spectroscopic data were comparable to those obtained for **8** [32]. Although the ^1^H and ^13^C NMR data of **8** agreed with those published for sulfuretin, the previously reported resonance values for H-4 (δ 6.84) and H-5′ (δ 7.59) should be interchanged. This assumption was substantiated by correlations observed in the ^1^H-^1^H COSY spectrum of **8** between H-4 (δ 7.56) and H-5 (δ 6.72) and between H-5′ (δ 6.89) and H-6′ (δ 7.27), together with long-range connectivities observed in the HMBC spectrum from H-5′ to C-3′ (146.4) and C-6′(125.4), thus allowing unambiguous assignments of H-4 and H-5′ resonances as shown. Despite being present in various plant sources, the isolation of sulfuretin from *C. laxum* is noteworthy, because not only is it being reported for the first time in the Combretaceae, but also it is the first occurrence of aurones in this family.

Compound **9** exhibited in its ^1^H NMR spectrum characteristic signals of a 5,7,4′-trioxygenated flavone, viz. a typical proton singlet at δ_H_ 6.48 ascribed to H-3, along with a pair of broad singlets at δ_H_ 6.34 and 6.46 of *meta*-coupled protons at ring A (H-6 and H-8, respectively), and a pair of doublets of a *para*-oxygenated ring B at δ_H_ 7.78 (2H, *J* = 9.0 Hz, H-2′/H-6′) and 6.89 (2H, *J* = 9.0 Hz, H-3′/H-5′) [31]. This spectrum also showed a three-proton singlet at δ_H_ 3.85 ascribed to a methoxy group. Characteristic signals of ring C carbons were observed at δ_C_ 161.6 (C-2), 106.2 (C-3), and 180.2 (C-4) [33]. The linkage of the methoxyl group to C-5 was established by HMBC coupling of the methoxyl hydrogens to C-5 (δ_C_ 162.4), which was corroborated by a correlation discernible in the NOESY spectrum between H-6 and the methoxyl hydrogens. Therefore, compound **9** was shown to be 5-methoxy-7,4′-dihydroxyflavone, also known as 5-*O*-methyl apigenin, whose spectroscopic data were comparable to those of **9** [34]. This flavone derivative, with restricted distribution in plant species, is being reported for the first time in the Combretaceae.

The ^1^H NMR spectrum of **10** showed a pair of doublets at δ_H_ 8.05 and 8.10 (*J* = 6.0 Hz) and a pair of triplets of doublets at δ_H_ 7.73 and 7.66 (*J* = 7.0 and 1.5), which, together with the chemical shifts observed in the ^13^C NMR spectrum in the range of δ_C_ 126–135, were attributed to an *ortho*-substituted aromatic ring. Evidence of a 2-hydroxy-1,4-naphthoquinone skeleton bearing a side chain at C-3 was given by the presence of two carbonyl resonances at δ_C_ 181.7 and 184.5, as well as the signals of two substituted sp^2^ carbons at δ_C_ 123.5 and 152.7. The nature of the side chain was promptly established as a prenyl group, based on its characteristic methyl singlets at δ_H_/δ_c_ 1.67/25.7 and 1.77/17.9, as well as the signals ascribed to a trisubstituted double bond linked to methylene group, evidenced by the one-proton broad triplet at δ_H_ 5.19 (*J* = 6.0 Hz) and a two-proton doublet at δ_H_ 3.29 (*J* = 6.0 Hz), respectively. Accordingly, the remaining carbon resonances of the prenyl group were observed at δ_C_ 119.6 (C-2′), 133.8 (C-3′) and 22.6 (C-1′). Connectivities discernible from HSQC and HMBC experiments provided further evidence for the structure of compound **10**, which was identified as shown. Its ^1^H and ^13^C NMR data were in accordance with those reported in the literature for the 1,4-naphthoquinone known as lapachol [35]. Lapachol, which occurs in a number of plants belonging to several families, has long been recognized for its wide array of biological activities, particularly significant antitumor-promoting effects [36,37]. The isolation of lapachol from *C. laxum* is remarkable, since, to our knowledge, no reports on the occurrence of this or any other naphthoquinone representatives in the Combretaceae have hitherto been found in the literature.

Compounds **11** and **12** were readily identified as the benzoic acid derivatives 3,4-dimethoxybenzoic and 3-hydroxy-4-methoxybenzoic acids, respectively, whose NMR data were in full agreement with those reported in the literature [38].

### 2.2. In Vitro Cytotoxic Evaluations

Given the known antineoplastic potentialities of phenanthrene, dihydrophenanthrene and dihydrostilbenoid derivatives, particularly those obtained from members of the Combretaceae and Orchidaceae [7,39,40], compounds **1**-**6** were further assessed for their in vitro antiproliferative effects against five human neoplastic cell lines (except for **1** and **3**, which were tested against four cell lines due to insufficient material), based on the SRB (sulforhodamine B) assay and using cisplatin as a positive control.

**Figure 2:**
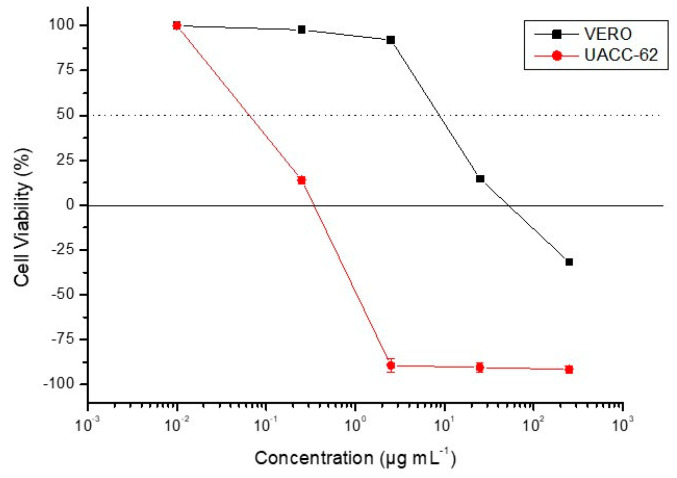
Effect of 6-methoxycoelonin (**4**) [0.25, 2.5, 25, and 250 µg mL^−1^] on cell viability in UACC-62 human melanoma and VERO nonneoplastic cell lines.

As depicted in Table 3, all compounds showed inhibitory activities against at least one of the five cell lines tested, with 6-methoxycoelonin (**4**) displaying a remarkable effect against UACC-62 cells (IC_50_ 2.59 ± 0.11 µM). This dihydrophenanthrene derivative proved not only seven times more active against this cell line than cisplatin, but also at least 86 times more potent than the other compounds tested. This result led us to assess the effect of **4** on nontumor mammalian VERO cells in order to determine its selectivity index. The obtained IC_50_ value, 65.12 ± 4.51 µM, revealed that **4** is roughly 25 times more selective for UACC-62 cells than for nontumor cells (SI = 25.1). In addition, **4** inhibited the proliferation of UACC-62 and VERO cells in a dose-dependent manner. As shown in Figure 2, the points above zero in the curves indicate that 6-methoxycoelonin had a cytostatic (growth inhibition) effect on UACC-62 and VERO cells at the concentrations of 0.25, 2.5 and 25 µg mL^−1^. In addition, as revealed by the points below zero in the growth curves, while **4** had a cytocidal (cell death) effect on UACC-62 cells from the concentration of 2.5 µg mL^−1^, the viability of nonneoplastic VERO cells at this same concentration remained close to 100% (*p* < 0.5).

A literature survey on the cytotoxic potentialities of the known compounds **2**–**6** revealed that the antiproliferative effects of 6-methoxycoelonin (**4**) against UACC-62, 786-0, Hep-2, and NCI/ADR RES cells are being described for the first time, while, to our knowledge, 2,7-dihydroxy-4,6-dimethoxyphenanthrene (**2**), 2,6-dihydroxy-3,4,7-trimethoxyphenanthrene (**3**), 2,6-dihydroxy-3,4,7-trimethoxy-9,10-dihydrophenanthrene (**5**), and callosin (**6**) have not yet been screened for their in vitro cytotoxic properties against any neoplastic cell line. Regarding the earlier reported effects of **4** against MCF-7 cells, two different IC_50_ values, namely 9.58 and 37.9 µM, were described for 6-methoxycoelonin [19,41], the latter being closer to that obtained in the present study.

Based on the IC_50_ values obtained for phenanthrene **2** compared with those of its corresponding 9,10-dihydro derivative **4**, cytotoxicity is significantly enhanced in the latter by reduction of carbons C-9 and C-10, particularly against UACC-62 cells, wherein **4** was at least 96 times more potent than **2**. Nevertheless, the assumption that cytotoxicity of the phenanthrenes and dihydrophenanthrenes might be directly related to the lack of aromaticity in ring B does not apply to phenanthrene **3** and its corresponding 9,10-dihydro derivative **5**, since their cytotoxic effects do not follow the same uniform pattern against the cells tested as that of **2** and **4**. Another significant feature observed for the tested dihydrophenanthrenes is that minimal structural differences, as that found between **4** and **6**, may account for expressive effects on their cytotoxic potentials. Accordingly, as demonstrated by their IC_50_ values, activity of **6**—which only differ from **4** by the change in position of the methoxyl and hydroxyl functionalities in ring A, at C-6 and C-7—is remarkably reduced against all cell lines when compared with that of **4**. Likewise, by analyzing the effects of compounds **2**, **4** and **6**, the inversion between the substituents at C-6 and C-7 (as in **4** and **6**) leads to a greater reduction of cytotoxicity than that caused by the maintenance of the aromaticity of ring in **2** when compared to its dihydro derivative **4**. On the other hand, regarding the cytotoxic effects of **6** and **5**, the presence of an additional methoxy substituent at C-3 renders **5** more potent against all cell lines tested. Therefore, at least with respect to the effects of the foregoing compounds against UACC-62 cells, the presence of methoxy and hydroxy groups at C-6 and C-7, respectively, as well as the lack of aromaticity of ring B can be considered as important structural features for cytotoxicity. Although no extensive studies on structure/activity relationships for natural phenanthrenes and dihydrophenanthrenes have been reported in the literature, some results from previous works suggest the relevance of the numbers and the substituted positions of methoxy and hydroxy groups in the phenanthrene/dihydrophenanthrene skeleton for the cytotoxic activity of these classes of natural compounds [19,20,42,43,44,45].

### 2.3. DPPH-Radical-Scavenging Assay

Antioxidants are known by their effects in the prevention of several oxidative stress associated diseases, such as cancer, given their ability to inhibit the oxidative damage to DNA caused by scavenging free radicals [46,47]. In this sense, the antioxidant potential of compounds **1**–**6** were further evaluated using the DPPH-radical-scavenging assay, in order to find whether the radical-scavenging ability of the structurally related compounds **2**–**6** correlated with their anticancer potential. As depicted in Table 4, compounds **2**, **5**, and **6** showed radical-scavenging capacity of similar potencies to the positive control caffeic acid, with IC_50_ values ranging from 17.7 ± 0.25 to 32.9 ± 0.25 µM, while **1**, **3**, and **4** had lower activities (IC_50_ values between 45.6 ± 0.35 and 56.5 ± 0.29 µM). Based on the results obtained for **2** and **4**, it can be inferred that unsaturation at C-9/C-10 had a positive influence on the antioxidant capacity of **2** in this assay. In contrast, however, a decrease in the antioxidant ability of **3** was observed when compared with that of **5**, thus suggesting that other structural features, such as the presence and/or nature of oxygenated substituents, might play key roles in the radical-scavenging capacity of phenanthrenes and their corresponding dihydro derivatives. Accordingly, significant differences in the DPPH-scavenging properties were observed for **4** and **6**, although their structures only differ for the position of the hydroxy and methoxy groups at C-6 and C-7. Likewise, the introduction of a methoxy group at C-3, as in **5**, lowers its scavenging capacity when compared with that of its structural related compound **6**. The foregoing results also revealed that callosin (**6**), despite exhibiting the strongest DPPH-scavenging activity, was the least cytotoxic compound, indicating that, at least for the cell lines tested, antioxidant potential is unrelated to antiproliferative activity. Several phenanthrene and dihydrophenathrene derivatives are known for their DPPH scavenging properties [39]. However, literature data reveal that, as observed for compounds **2**–**6**, no relevant structure-activity relationships could clearly be established within these chemical classes, being postulated that the antioxidant capacity seemed to be related with the number of phenolic hydroxyl groups, either alone or together with methoxy groups, among other particular structural characteristics [45,48,49]. Despite a number of reports on the cytotoxic potential of plant extracts together with their radical scavenging activity against DPPH, particularly from the Orchidaceae, which is recognized as a rich source of these phenanthrene- and dihydrophenathrene-type compounds [37,38,50,51,52], no comprehensive studies on possible relationships between structure and DPPH-scavenging/cytotoxic properties for these classes of compounds have, to our knowledge, been previously described.

## 3. Materials and Methods

### 3.1. General Experimental Procedures

HRESIMS data were acquired with electrospray ionization in negative ion mode on an UltrOTOF-Q instrument (Bruker Daltonics, Billerica, MA, USA). NMR spectroscopic data were recorded at room temperature in CDCl_3_, acetone-*d*_6_, CD_3_OD, and/or pyridine-d_5_ (Cambridge Isotope Laboratories, Andover, MA, USA) on a Bruker DPX-300 spectrometer (Bruker, Karlhue, Germany) operating at 300.13 MHz (^1^H)/75.47 MHz (^13^C). Standard pulse sequences were used for homo- and heteronuclear correlation experiments. Optical rotation was determined on a Perkin Elmer 341 polarimeter (λ = 589 nm, PerkinElmer Inc., Waltham, MA, USA). Column chromatography procedures were performed on silica gel 60 (70–230 mesh, Merck, Darmstadt, Germany), silica gel 60 RP-18 (230–400 mesh, Merck, Darmstadt, Germany) and Sephadex LH-20 (Amersham Biosciences, Buckinghamshire, UK). Reversed-phase semipreparative HPLC separations were carried out with a Shimadzu (Shimadzu, Kyoto, Japan) LC-6AD pump using a Phenomenex Luna RP-18 column (5 µm, 21.6 × 250 mm) at flow rates of 12 or 14 mL/min, with monitoring at 210, 230 or 254 nm.

### 3.2. Plant Material

Roots and stems of *C. laxum* were collected from Corumbá, Mato Grosso do Sul, Brazil, in July 2016. The plant material was identified by Prof. Arnildo Pott (Institute of Biosciences, Universidade Federal de Mato Grosso do Sul). A voucher specimen (no. 39343) has been deposited at the CGMS Herbarium of the Universidade Federal de Mato Grosso do Sul. License for research on Brazil’s biodiversity, #A5DBC20.

### 3.3. Extraction and Isolation

Air-dried and powdered roots of *C. laxum* (1422 g) were extracted at room temperature with EtOH. After concentration in vacuo, the residue obtained from the EtOH extract was partitioned between *n*-butanol and H_2_O 1:1. The resulting syrupy *n*-butanol phase was subsequently partitioned between MeOH–H_2_O 9:1 and hexane, and between MeOH–H_2_O 1:1 and CH_2_Cl_2_ to give the corresponding hexane (2.24 g) and CH_2_Cl_2_ (4.52 g) phases. The CH_2_Cl_2_ phase was chromatographed on a silica gel 70–230 mesh column, using step gradient elution with hexane, hexane–CH_2_Cl_2_ (1:1), CH_2_Cl_2_, CH_2_Cl_2_–EtOAc (1:1), and EtOAc to give 12 fractions (A → L). Fraction F (CH_2_Cl_2_ 100%, 232.2 mg) was further separated by CC on Sephadex LH-20 (MeOH) to furnish six subfractions (F.1 → F.6). Compounds **3** (3.2 mg) and **4** (4.5 mg) were obtained from subfraction F.4 (22.0 mg), after reversed-phase semipreparative HPLC (MeO–H_2_O 40:60, 4.0 mL/min., 254 nm), while **2** (33.1 mg) was obtained from subfraction F.5. Fraction H (CH_2_Cl_2_–EtOAc 1:1, 1159.0 g) was chromatographed on RP-18 silica gel 230–400 mesh column by elution with a MeOH–H_2_O gradient (4:6, 6:4, 8:2) and MeOH, to afford four subfractions (H.1 → H.4). Compounds **1** (3.1 mg), **5** (14.2 mg), **6** (6.9 mg), **7** (6.9 mg), and further amounts of **4** (4.5 mg) were isolated from subfraction H.1 (MeOH–H_2_O 4:6, 152.3 mg), after reversed-phase semipreparative HPLC (MeCN–H_2_O 28:72, 14 mL/min., 254 nm). Reversed-phase semipreparative HPLC (MeCN–H_2_O 48:52, 14 mL/min., 254 nm) of subfraction H.3 (MeOH–H_2_O 8:2, 358.4 mg) yielded **13** (40.8 mg). Compounds **14** (8.5 mg) and **15** (5.3 mg) were obtained from subfraction H.4 (MeOH, 350.3 mg), after column chromatography on RP-18 silica gel 230–400 mesh, using step gradient elution with MeCN–H_2_O 6:4, 8:2, and MeOH 100%, followed by reversed-phase semipreparative HPLC (MeCN–H_2_O 60:40, 14 mL/min., 210 nm).

Air-dried and powdered stems of *C. laxum* (2760 g) were extracted at room temperature with EtOH. After concentration in vacuo, the residue obtained from the EtOH extract was subsequently partitioned between MeOH/H_2_O 9:1 and hexane, and between MeOH/H_2_O 1:1 and CH_2_Cl_2_ to give the corresponding hexane (1.50 g) and CH_2_Cl_2_ (1.10 g) phases. The CH_2_Cl_2_ phase was chromatographed on a silica gel 70–230 mesh column, using hexane, hexane–CH_2_Cl_2_ (3:1, 1:1, 1:3), CH_2_Cl_2_, CH_2_Cl_2_-EtOAc (3:1, 1:1, 1:3), EtOAc, and EtOAc–MeOH (9:1, 3:1, 1:1) as eluents, to furnish 12 fractions (A → L). Fraction D (hexane–CH_2_Cl_2_ 1:3) gave **10** (8.1 mg), while fraction H (CH_2_Cl_2_ 1:3, 104.9 mg) yielded **8** (3.0 mg), **11** (6.5 mg), and **12** (2.3 mg), after reversed-phase semipreparative HPLC (MeCN–H_2_O 28:72, 12 mL/min., 210 nm). Fraction J (EtOAc–MeOH 9:1, 150.6 mg) was re-chromatographed on Sephadex LH-20 (MeOH) to afford five subfractions (J.1 → J.5). Subfraction J.2 gave compound **13** (33.8 mg), while compound **9** (2.5 mg) was obtained from subfraction J.4 (11.3 mg), after separation by reversed-phase semipreparative HPLC (MeOH–H_2_O 62:38, 12 mL/min., 254 nm).

4′-Hydroxy-3,3′,4-trimethoxy-5-(3,4,5-trimethoxyphenoxy)-bibenzyl (**1**): amorphous solid; HRESIMS *m/z* 509.1588 [M + K]^+^ (calcd for C_26_H_30_O_8_K, 509.1572); ^1^H and ^13^C NMR data (Table 1).

2,7-Dihydroxy-4,6-dimethoxyphenanthrene (**2**): amorphous solid; HRESIMS *m/z* 271.0963 [M + H]^+^ (calcd for C_16_H_15_O_4_, 271.0970); ^1^H and ^13^C NMR data (Table 2).

2,6-Dihydroxy-3,4,7-trimethoxyphenanthrene (**3**): amorphous solid; HRESIMS *m/z* 301.1076 [M + H]^+^ (calcd for C_17_H_17_O_5_, 301.1076); ^1^H and ^13^C NMR data (Table 2).

6-Methoxycoelonin (**4**): amorphous solid; HRESIMS *m/z* 273.1118 [M + H]^+^ (calcd for C_16_H_17_O_4_, 273.1126); ^1^H and ^13^C NMR data (Table 2).

2,6-Dihydroxy-3,4,7-trimethoxy-9,10-dihydrophenanthrene (**5**): amorphous solid; HRESIMS *m/z* 303.1235 [M + H]^+^ (calcd for C_17_H_19_O_5_, 303.1233); ^1^H and ^13^C NMR data (Table 2).

Callosin (**6**): amorphous solid; HRESIMS *m/z* 273.1121 [M + H]^+^ (calcd for C_16_H_17_O_4_, 273.1126); ^1^H and ^13^C NMR data (Table 2).

(+)-Syringaresinol (**7**): amorphous solid; [α]_D_^20^ + 11.1 (*c* 0.23, CH_3_OH); ^1^H-NMR (CD_3_OD): δ 3.13 (2H, brs, H-8, H-8′); 3.70-3.80 (2H, m; H-9a, H-9′a); 3.84 (12H, s, OCH_3_-3, 3′, 5, 5′); 4.18-4.24 (2H, m, H-9b, H-9′b); 4.70 (2H, d, *J* = 3.0 Hz, H-7, H-7′); 6.65 (4H, s, H-2, H-2′, H-6, H-6′). ^13^C-NMR (CD_3_OD): δ 133.1 (C-1, C-1′); 104.5 (C-2, C-2′, C-6, C-6′); 149.3 (C-3, C-3′, C-5, C-5′); 136.2 (C-4, C-4′); 87.6 (C-7, C-7′); 55.5 (C-8, C-8′); 72.5 (C-9a, C-9′a); 56.8 (OCH_3_-3, 3′, 5, 5′).

Sulfuretin (**8**): amorphous solid; ^1^H-NMR (acetone-d_6_): δ 6.58 (1H, s, H-10); 6.72 (1H, dd, *J* = 8.5 and 1.5 Hz, H-5); 6.79 (1H, d, *J* = 1.5 Hz, H-7); 6.89 (1H, d, *J* = 8.3 Hz, H-5′); 7.27 (1H, dd, *J* = 8.3 and 2.2 Hz, H-6′); 7.56 (1H, d, *J* = 8.5 Hz, H-4). ^13^C-NMR (acetone-d_6_): δ 147.2 (C-2), 182.5 (C-3); 126.3 (C-4); 113.8 (C-5); 167.7 (C-6); 99.4 (C-7); 169.0 (C-8); 114.4 (C-9); 112.4 (C-10); 125.2 (C-1′); 118.7 (C-2′); 146.4 (C-3′); 148.6 (C-4′); 116.6 (C-5′); 125.4 (C-6′).

5-*O*-Methyl Apigenin (**9**): amorphous solid; ^1^H-NMR (CD_3_OD): δ 3.85 (3H, s, OCH_3_-5); 6.34 (1H, brs, H-6); 6.46 (1H, brs, H-8); 6.48 (1H, s, H-3); 6.89 (2H, d, *J* = 9.0 Hz, H-3, H-5′′); 7.78 (2H, d, *J* = 9.0 Hz, H-2′, H-6′). ^13^C-NMR (CD_3_OD): δ 161.6 (C-2); 106.2 (C-3); 180.2 (C-4); 162.4 (C-5); 98.7 (C-6); 163.5 (C-7); 97.2 (C-8); 123.4 (C-1′); 129.0 (C-2′, C-6′); 117.0 (C-3′, C-5′); 162.3 (C-4′); 56.3 (OCH_3_-5).

Lapachol (**10**): amorphous solid; ^1^H-NMR (CDCl_3_): δ 1.67 (3H, s, H-4′); 1.77 (3H, s, H-5′); 3.29 (2H, d, *J* = 6.0 Hz, H-1′); 5.19 (1H, brt, *J* = 6.0 Hz, H-2′); 7.66 (1H, td, *J* = 7.0 and 1.5 Hz, H-7); 7.73 (1H, td, *J* = 7.0 and 1.5 Hz, H-6); 8.05 (1H, d, *J* = 6.0 Hz, H-8); 8.10 (1H, d, *J* = 6.0 Hz, H-5). ^13^C-NMR (CD_3_OD): δ 181.7 (C-1); 152.7 (C-2); 123.5 (C-3); 184.5 (C-4); 126.8 (C-5); 134.8 (C-6); 132.8 (C-7); 126.0 (C-8); 129.4 (C-9); 132.9 (C-10); 22.6 (C-1′); 119.6 (C-2′); 133.8 (C-3′); 25.7 (C-4′); 17.9 (C-5′).

3,4-Dimethoxybenzoic Acid (**11**): amorphous solid; ^1^H-NMR (CD_3_OD): δ 3.85 * (3H, s, OCH_3_-3); 3.87 * (3H, s, OCH_3_-4); 6.98 (1H, d, *J* = 9.0 Hz, H-5); 7.54 (1H, brs, H-2); 7.64 (1H, brd, *J* = 9.0 Hz, H-6). ^13^C-NMR (CD_3_OD): δ 124.3 (C-1); 113.6 (C-2); 150.0 (C-3); 154.6 (C-4); 111.8 (C-5); 125.0 (C-6); 169.9 (C-7); 56.4 * (OCH_3_-3 and OCH_3_-4) * interchangeable signals.

3-Hydroxy-4-methoxybenzoic Acid (**12**): amorphous solid; ^1^H-NMR (CD_3_OD): δ 3.89 (3H, s, OCH_3_-4); 6.83 (1H, brd, *J* = 9.0 Hz, H-5); 7.55 (1H, d, *J* = 9.0 Hz, H-6); 7.56 (1H, brs, H-2). ^13^C-NMR (CD_3_OD): δ 123.6 (C-1); 113.8 (C-2); 152.4 (C-3); 148.6 (C-4); 115.8 (C-5); 125.2 (C-6); 169.8 (C-7); 56.4 (OCH_3_-4).

Arjunolic Acid (**13**): amorphous solid; ^1^H-NMR (CD_3_OD): δ 0.68 (3H, s, H-24); 0.80 (3H, s, H-26); 0.91 (3H, s, H-29); 0.93 (3H, s, H-30); 1.01 (3H, s, H-25); 1.15 (3H, s, H-27); 2.84 (1H, dd, *J* = 12.8 and 3.4 Hz, H-18); 3.30 (1H, d, *J* = 9.0 Hz, H-3); 3.33 (1H, d, *J* = 12.0 Hz, H-23a); 3.49 (1H, d, *J* = 12.0 Hz, H-23b); 3.68 (1H, m, H-2); 5.24 (1H, brs, H-5); ^13^C-NMR (CD_3_OD): δ 47.6 (C-1); 69.7 (C-2); 78.2 (C-3); 44.1 (C-4); 48.2 (C-5); 19.1 (C-6); 33.8 (C-7); 40.5 (C-8); 47.9 (C-9); 39.0 (C-10); 24.0 * (C-11); 123.4 (C-12); 145.4 (C-13); 43.0 (C-14); 28.8 (C-15); 24.6 * (C-16); 47.6 (C-17); 42.7 (C-18); 47.2 (C-19); 31.6 (C-20); 34.9 (C-21); 33.3 (C-22); 66.4 (C-23); 13.9 (C-24); 17.8 (C-25); 17.6 (C-26); 26.5 (C-27); 181.9 (C-28); 33.6 (C-29); 24.0 (C-30) * interchangeable signals.

Betulinic Acid (**14**): amorphous powder; ^1^H- and ^13^C-NMR data in accordance with those of an authentic sample and with literature [8].

Maslinic Acid (**15**): amorphous solid; ^1^H-NMR (pyridine-d_5_): δ 0.93 (3H, s, H-30); 0.97 (3H, s, H-29); 0.98 (3H, s, H-25); 1.00 (3H, s, H-23); 1.06 (3H, s, H-26); 1.25 (3H, s, H-27); 1.26 (3H, s, H-24); 3.30 (1H, m, H-18); 3.38 (1H, d, *J* = 10.0 Hz, H-3); 4.11 (1H, ddd, *J* = 13.0, 9.0 and 3.0 Hz); 5.45 (1H, brs; H-12). ^13^C-NMR (pyridine-d_5_): δ 46.4 (C-1); 68.6 (C-2); 83.8 (C-3); 39.8 (C-4); 55.9 (C-5); 18.8 (C-6); 33.2 (C-7); 39.8 (C-8); 48.1 (C-9); 38.5 (C-10); 23.7 (C-11); 122.4 (C-12); 144.8 (C-13); 42.2 (C-14); 28.2 (C-15); 23.7 (C-16); 46.6 (C-17); 42.0 (C-18); 46.4 (C-19); 30.9 (C-20); 34.2 (C-21); 33.2 (C-22); 29.3 (C-23); 17.6 (C-24); 16.8 (C-25); 17.4 (C-26); 26.1 (C-27); 180.2 (C-28); 33.3 (C-29); 23.7 (C-30).

### 3.4. In Vitro Cytotoxic Assay

Cytotoxicity of compounds **1**–**6** was evaluated against five human neoplastic cell lines—namely, MCF-7 (breast), 786-0 (kidney), UACC-62 (melanoma), NCI/ADR-RES (ovary, multidrug-resistant phenotype), and Hep2 (larynx), all of which were kindly provided by Prof. João Ernesto de Carvalho, of the School of Pharmaceutical Sciences, CPQBA, Universidade Estadual de Campinas, Campinas, Brazil. VERO (monkey kidney) nonneoplastic cells were obtained from the Rio de Janeiro cell bank. To this end, a sulforhodamine B (SRB; purity ≥ 97%; Sigma, St. Louis, MO, USA) assay was performed, as described elsewhere [53,54]. Cisplatin (purity ≥ 99.9%; Sigma, St. Louis, MO, USA) was used as the positive control. Each sample was tested in triplicate at four different concentrations (0.25, 2.5, 25, and 250 μg mL^−1^). IC_50_ values were calculated from the differences in absorbance readings at 540 nm in untreated (negative control) and treated cells on a SpectraMax 190 microplate reader (Molecular Devices, San Jose, CA, USA) [53] through nonlinear regression analysis, using Origin 6.0 software (OriginLab, Northampton, MA, USA), and growth percentages were calculated as described elsewhere [53]. The data presented are the mean ± standard deviation of at least three independent cell preparations made in triplicate. Statistical analysis was performed with OriginPro 9.55 (OriginLab, Northampton, MA, USA) applying t-test for pairwise comparison (threshold value *p* < 0.05).

### 3.5. DPPH-Radical-Scavenging Assay

The radical-scavenging activities of compounds **1**–**6** were determined using DPPH (a stable free radical), employing the method of microdilution in 96-well microplates described by Zhang et al. and Yamaguchi et al. [49,55], with some modifications. The assays were performed in triplicate, using caffeic acid as a standard compound and a DPPH solution in EtOH (200 µM) as a negative control. Solutions of samples in EtOH at 200 µM were serially diluted to 100, 50, 25, 12.5, and 6.25 µM. Each solution (100 µL) was mixed with 100 µL of DPPH solution. The samples were allowed to stand at room temperature in the dark for 30 min, after which their absorbances were recorded at 515 nm. The ability of test materials to scavenge DPPH radicals was calculated as follows: DPPH scavenging effect (%) = 100 (A_control_ − A_sample_)/A_control_. Radical-scavenging activities were assessed on the basis of their IC_50_ values determined by linear regression.

## 4. Conclusions

The foregoing results constitute new information on the chemical composition of a specimen of *C. laxum* from the Brazilian Pantanal. Among the one new and 14 known compounds comprising nine different classes of secondary metabolites, the isolation of dihydrostilbenoid, phenanthrene and dihydrophenanthrene derivatives is unprecedented in a *Combretum* species native to the American continent. Also remarkable is the presence of aurone and naphthoquinone representatives, since these chemical classes are being reported for the first time in the Combretaceae, as are the isolation of phenanthrene **2**, dihydrophenanthrene **6**, and flavone **9**, and the first reported occurrence of lignan **7** in the genus *Combretum*. The results of the present study also revealed that, at least with respect to the effects of compounds **2**–**6** against melanoma (UACC-62) cells, the presence of methoxy and hydroxy groups at C-6 and C-7, respectively, as well as the lack of aromaticity of ring B can be considered as important structural features for cytotoxicity. On the other hand, when compared with their radical-scavenging ability against DPPH, cytotoxicity of **1**–**6** is unrelated to their antioxidant potential, at least for the five cancer cell lines tested.

The anticarcinogenic, antimetastatic, and chemopreventive potentialities of plant-derived compounds either isolated or in combination with chemotherapy drugs have been the subject of an increasing number of recent preclinical and clinical studies aiming at the development of new antineoplastic agents. These studies reveal that combination of synthetic chemotherapy drugs with selected plant constituents not only may improve pharmacological activity and simultaneously minimize toxic side effects of synthetic chemical drugs, but also delay or even overcome the development of drug resistance [56,57,58,59,60,61,62]. The IC_50_ and selectivity index values presented by **4** (6-methoxycoelonin) against melanoma (UACC-62) cells thus indicate that this dihydrophenanthrene derivative can be considered as a promising candidate for further investigation of its mechanism of action. Future research on association of 6-methoxycoelonin with current anticancer drugs aiming at the development of potential new drug combination therapies within clinical oncology is also strongly encouraged.

## Figures and Tables

**Table 1:** ^1^H (300 MHz) and ^13^C (75 MHz) nuclear magnetic resonance (NMR) data for compound **1** (CD_3_OD).

Position	δ_H_	δ_C_	HMBC (H → C)	
			^2^ *J*	^3^ *J*
1	**-**	139.3		
2	6.25 (d, 3.0)	105.5	C-3	C-4, C-6, C-1a
3	-	154.2		
4	-	135.8		
5	-	151.2		
6	6.30 (d, 3.0)	110.3	C-5	C-2, C-4, C-1a
1a	2.74 (m)	39.4	C-1, C-1b	C-2, C-6, C-1′
1b	2.76 (m)	38.6	C-1a, C-1′	C-1, C-2′, C-6′
1′	-	134.7		
2′	6.64 (d, 2.0)	113.5	C-1′, C-3′	C-1b, C-4′, C-6′
3′	-	148.7		
4′	-	145.6		
5′	6.68 (d, 9.0)	116.0	C-4′	C-1′, C-3′
6′	6.60 (dd, 9.0, 2.0)	122.0	-	C-1b, C-2′, C-4′
OCH_3_-4	3.74 (s)	61.0	-	C-4
OCH_3_-3	3.75 (s)	56.3	-	C-3
OCH_3_-3′	3.77 (s)	56.3	-	C-3′
1″	-	155.4		-
2″, 6″	6.09 (s)	94.0	C-1″, C-3″,5″	C-4″
3″, 5″	-	155.0		
4″	-	132.2	-	
OCH_3_-3″, 5″	3.77 (s)	56.3	-	C-3″,5″
OCH_3_-4″	3.67 (s)	61.3	-	C-4″

**Table 2:** ^1^H (300 MHz) and ^13^C (75 MHz) NMR data for compounds **2** (acetone-*d*_6_) and **3**–**6** (CD_3_OD).

Position	2		3		4		5		6	
	δ_H_	δ_C_	δ_H_	δ_C_	δ_H_	δ_C_	δ_H_	δ_C_	δ_H_	δ_C_
1	6.89 (d, 3.0)	105.4	7.04 (s)	109.8	6.30 (d, 3.0)	108.6	6.50 (s)	112.2	6.30 (d, 3.0)	108.5
2	-	156.1	-	150.4	-	157.6	-	150.2	-	157.8
3	6.79 (d, 3.0)	100.0	-	142.9	6.40 (d, 3.0)	99.4	-	141.3	6.39 (d, 3.0)	99.3
4	-	160.2	-	152.8	-	158.9	-	152.7	-	159.2
4a	-	115.5	-	118.9	-	116.9	-	120.9	-	116.5
4b	-	125.5	-	126.1	-	125.5	-	126.9	-	127.3
5	9.11 (s)	109.6	8.90 (s)	112.4	7.83 (s)	113.7	7.78 (s)	115.6	7.75 (s)	116.5
6	-	148.3	-	147.4	-	146.6	-	145.5	-	144.9
7	-	145.8	-	148.4	-	145.2	-	147.2	-	146.6
8	7.24 (s)	112.2	7.27 (s)	109.6	6.62 (s)	115.3	6.76 (s)	112.3	6.75 (s)	112.0
8a	-	128.2	-	128.1	-	132.2	-	130.9	-	130.8
9	7.56 (d, 9.0)	127.9	7.50 (d, 9.0)	127.3	2.58 (m)	32.1	2.60 (s)	30.4	2.62 (s)	32.2
10	7.44 (d, 9.0)	125.4	7.33 (d, 9.0)	124.9	2.60 (m)	30.2	2.60 (s)	31.5	2.62 (s)	30.4
10a	-	135.7	-	131.5	-	141.9	-	136.1	-	142.2
OCH_3_-3	-	-	4.00 (s)	61.5	-	-	3.85 (s)	61.3	-	-
OCH_3_-4	4.12 (s)	56.0	3.98 (s)	60.5	3.84 (s)	56.1	3.70 (s)	60.6	3.84 (s)	56.4
OCH_3_-6	4.02 (s)	56.2	-	-	3.84 (s)	56.7	-	-	-	-
OCH_3_-7	-	-	3.99 (s)	56.2	-	-	3.84 (s)	56.4	3.83 (s)	55.9

**Table 3:** Cytotoxicity of compounds **1**–**6** against human cancer cell lines (IC_50_, µM).

Compound	786-0	MCF-7	Hep2	UACC-62	NCI/ADR-RES
**1**	112.86 ± 2.89	72.69 ± 4.87	218.27 ± 2.52	NT	32.09 ± 4.31
**2**	73.26 ± 7.70	118.40 ± 9.29	> 250	> 250	83.99 ± 5.40
**3**	64.27 ± 9.62	226.10 ± 5.09	NT	246.75 ± 10.32	116.88 ± 2.66
**4**	56.98 ± 9.29	46.99 ± 5.55	207.93 ± 17.09	2.59 ± 0.11	58.83 ± 2.33
**5**	199.46 ± 6.75	42.01 ± 9.33	222.61 ± 2.81	221.62 ± 3.04	212.03 ± 14.06
**6**	257.14 ± 6.51	160.20 ± 8.21	547.58 ± 0.11	268.24 ± 13.8	303.02 ± 12.58
Cisplatin *	20.66 ± 2.67	22.00 ± 2.93	5.00 ± 0.23	18.66 ± 3.73	25.32 ± 1.43

Values represent means ± SD from three independent experiments. *—Positive control. NT: not tested. 786-0: kidney carcinoma; MCF-7: breast carcinoma; HEP-2: larynx carcinoma; UACC-62: human melanoma; NCI/ADR-RES: ovary carcinoma, multidrug-resistant phenotype.

**Table 4:** Radical-scavenging activity (assessed against DPPH) of compounds **1**–**6**.

Compound	IC_50_ (µM)
**1**	56.5 ± 0.3
**2**	20.4 ± 0.3
**3**	45.6 ± 0.3
**4**	55.6 ± 0.4
**5**	32.9 ± 0.3
**6**	17.7 ± 0.3
Caffeic acid (positive control)	10.9 ± 0.1

Values represent means ± SD from three independent experiments.

## Supplementary Materials

Supplementary Materials (Figures S1–S82) are available online.
