# Supplementary material for: Cytotoxic Phenanthrene, Dihydrophenanthrene, and Dihydrostilbene Derivatives and Other Aromatic Compounds from Combretum laxum
Source: Molecules. 2020 Jul 10;25(14):3154. doi: 10.3390/molecules25143154 (PMC7397156; doi:10.3390/molecules25143154)
Supplement: Supplementary file 1 [file molecules-25-03154-s001.pdf]

Supplementary Material

Cytotoxic phenanthrene, dihydrophenanthrene, and dihydrostilbene derivatives and other aromatic compounds from *Combretum laxum*

Eder Bisoli[1], Talita Vilalva Freire[1], Nídia Cristiane Yoshida[1], Walmir Silva Garcez[1], Lyara Meira Marinho Queiróz[2], Maria de Fátima Cepa Matos[2], Renata Trentin Perdomo[2] and Fernanda Rodrigues Garcez[1,*]

[1]Institute of Chemistry, Universidade Federal de Mato Grosso do Sul, Campo Grande, MS, Brazil; [2]Laboratory of Molecular Biology and Cell Culture, School of Pharmaceutical Sciences, Food Technology, and Nutrition, Universidade Federal de Mato Grosso do Sul, Campo Grande, MS, Brazil; *Correspondence: fernanda.garcez@ufms.br.

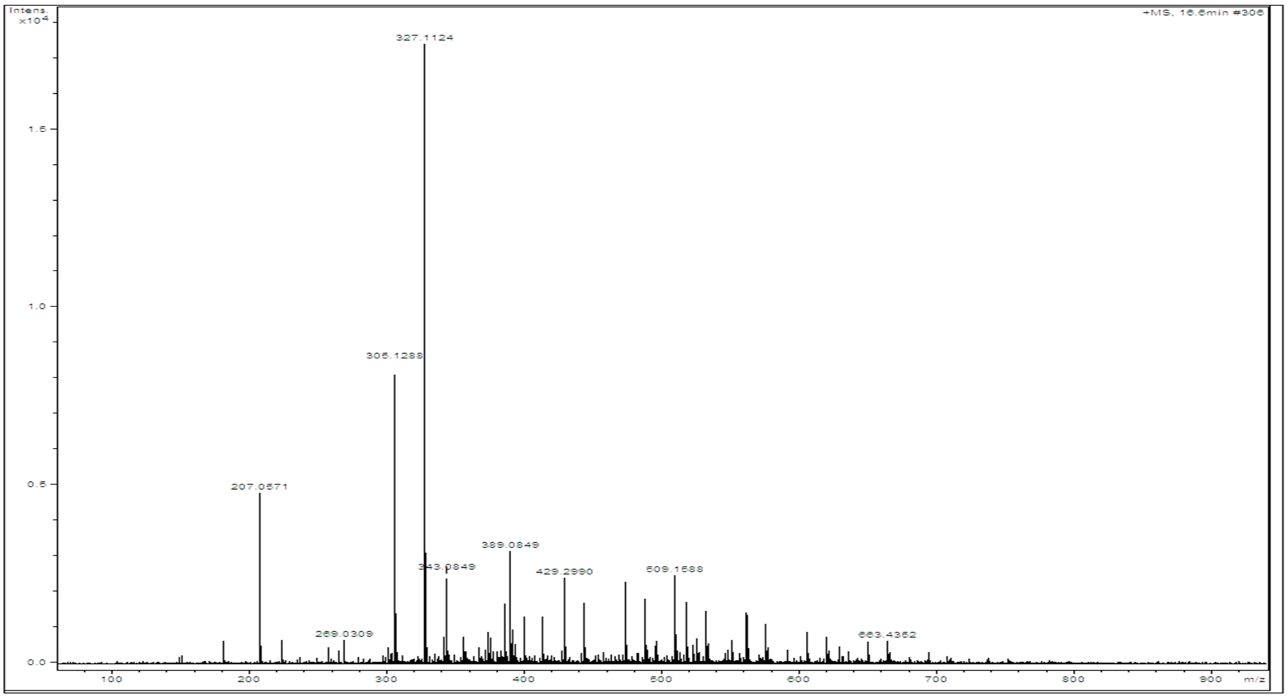

Figure S1. HRESIMS (positive mode) of compound **1**.

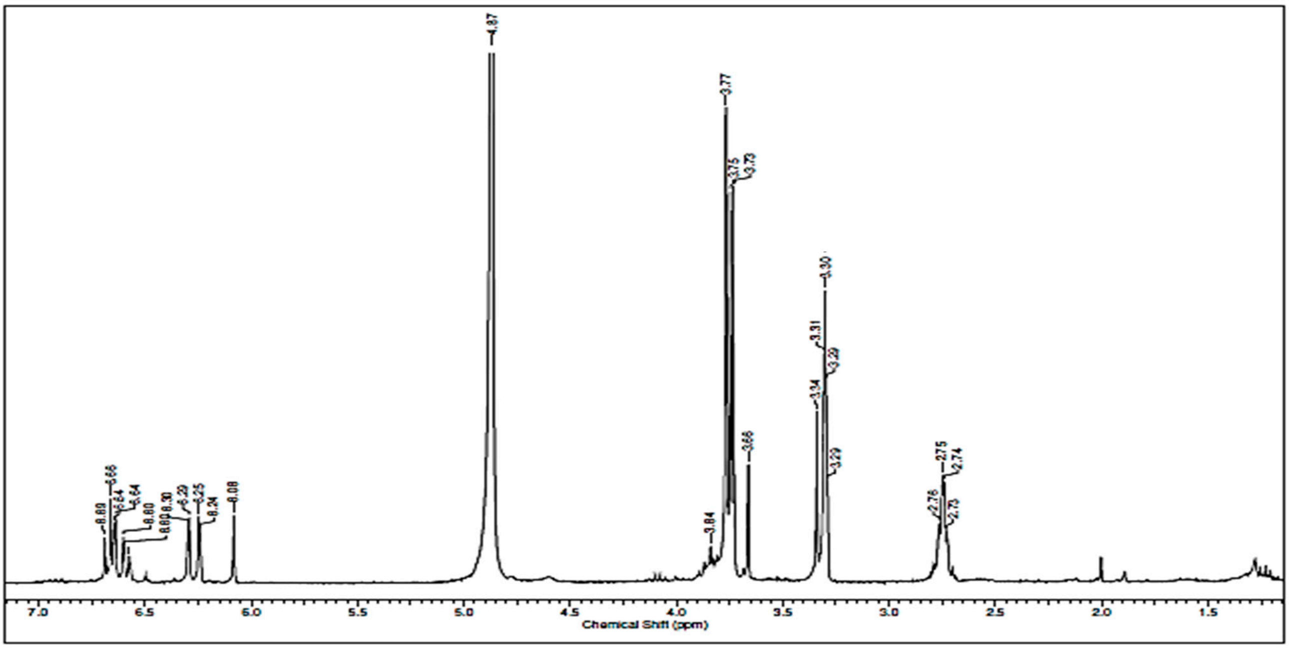

Figure S2. ^1H NMR spectrum (300 MHz, CD_3OD) of compound **1**.

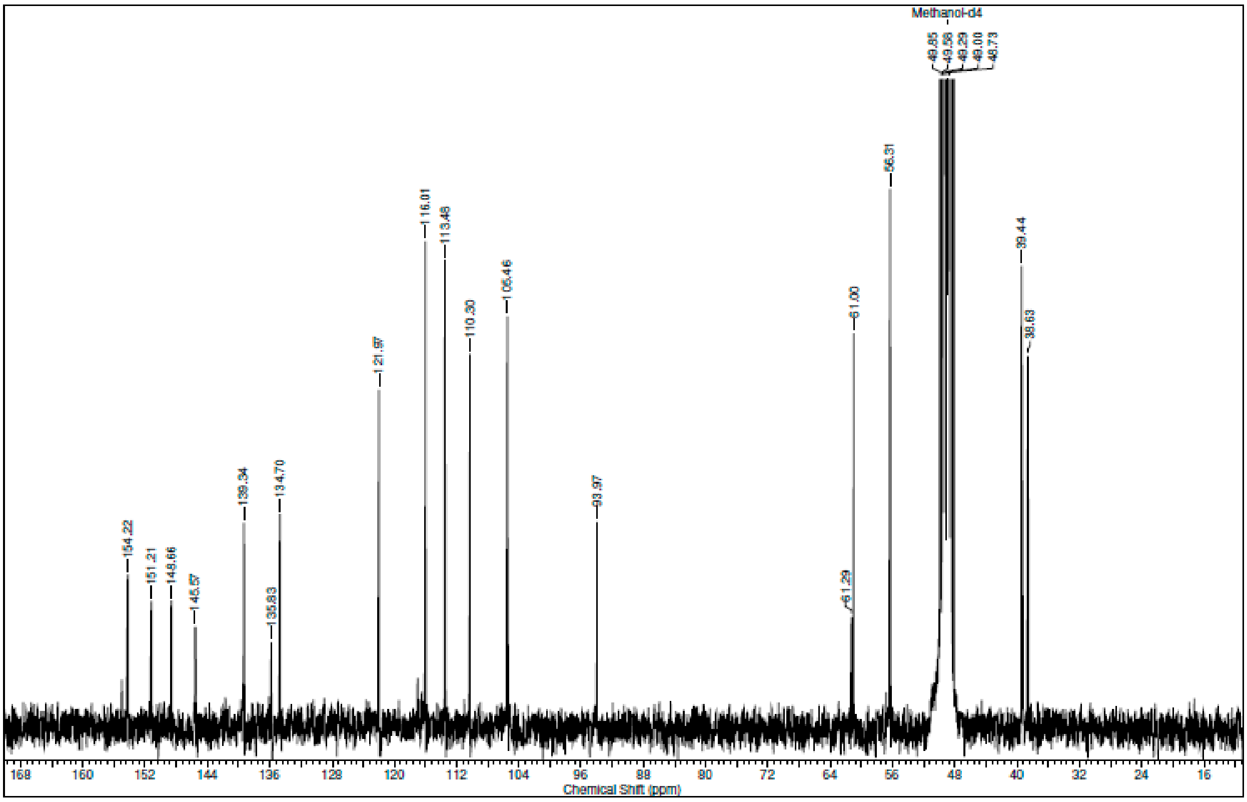

Figure S3. ^{13}C NMR spectrum (75 MHz, CD_3OD) of compound **1**.

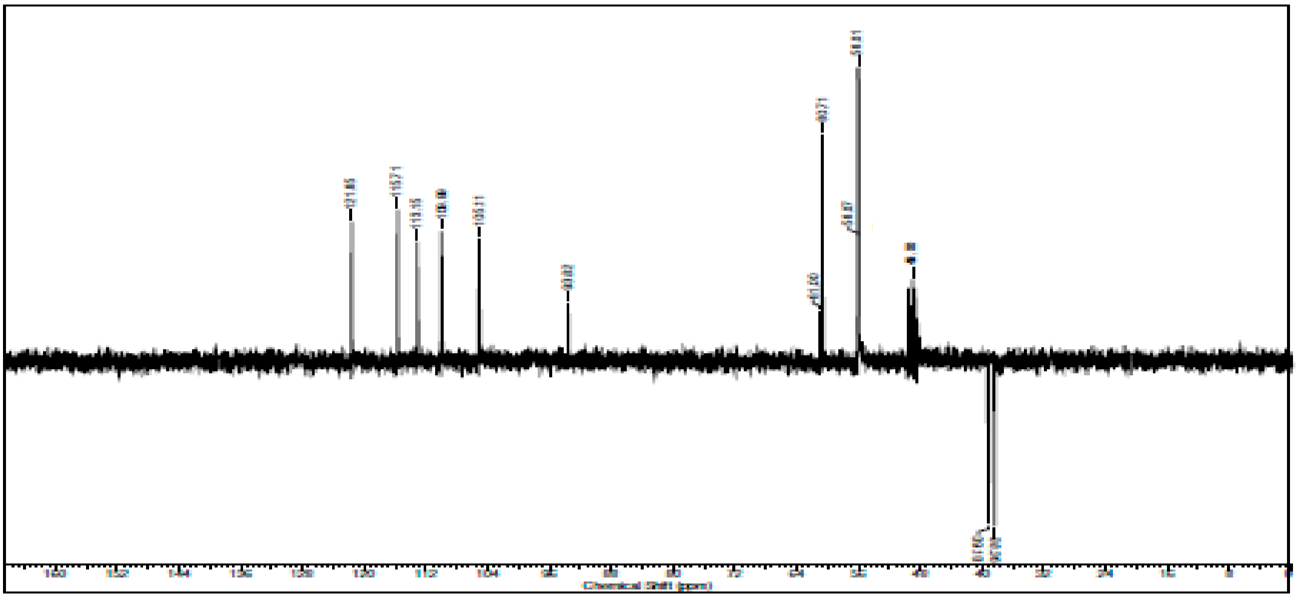

Figure S4. DEPT-135 spectrum (CD_3OD) of compound **1**.

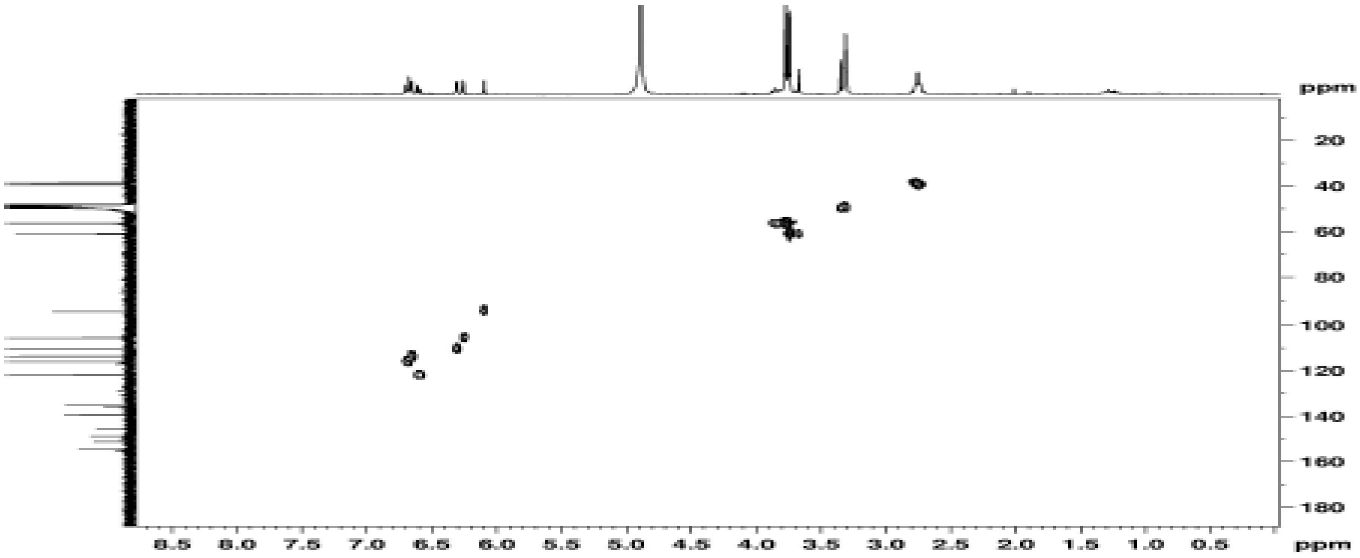

Figure S5. HSQC spectrum (CD_3OD) of compound **1**.

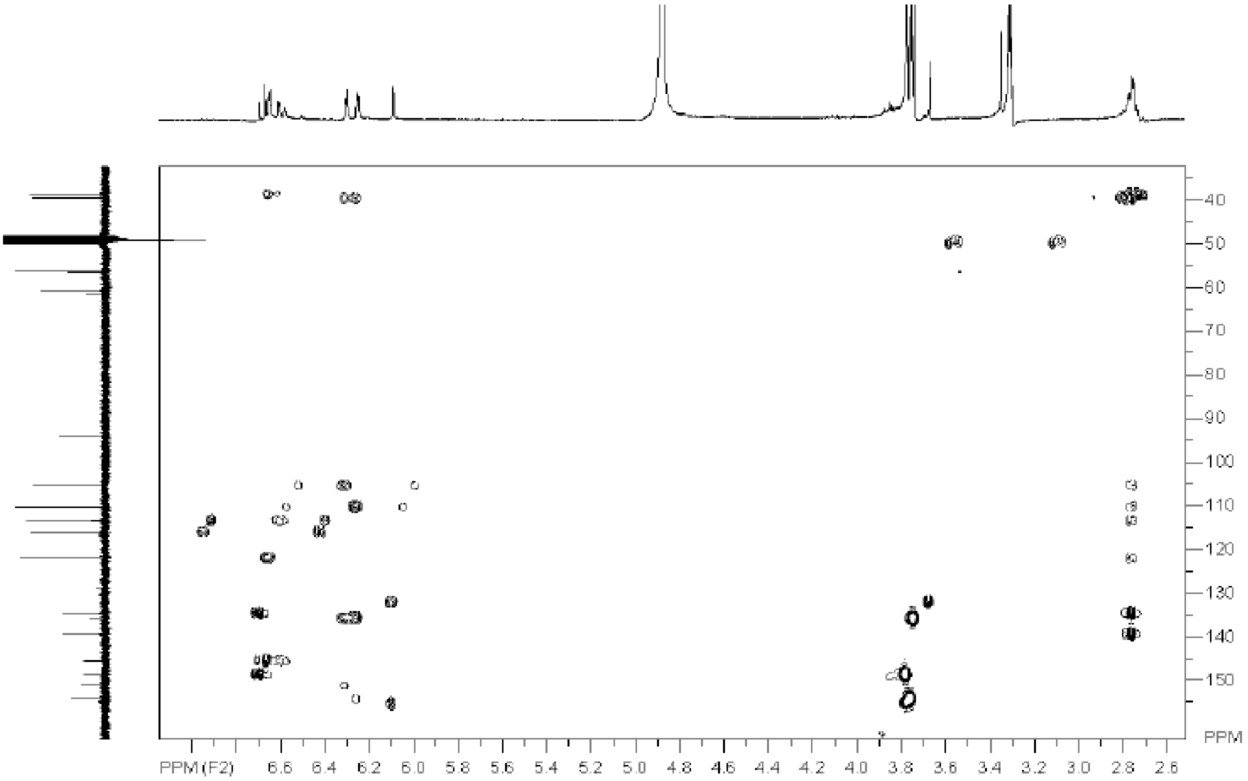

Figure S6. HMBC spectrum (CD_3OD) of compound **1**.

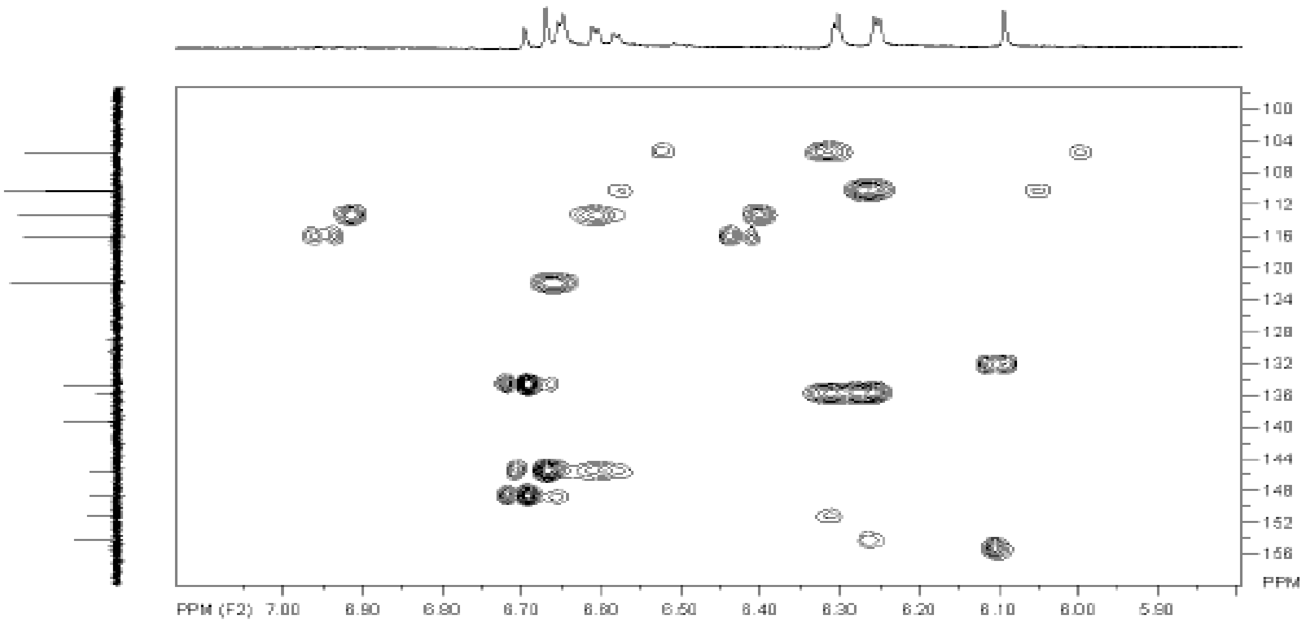

Figure S7. HMBC spectrum (expansion) of compound **1**.

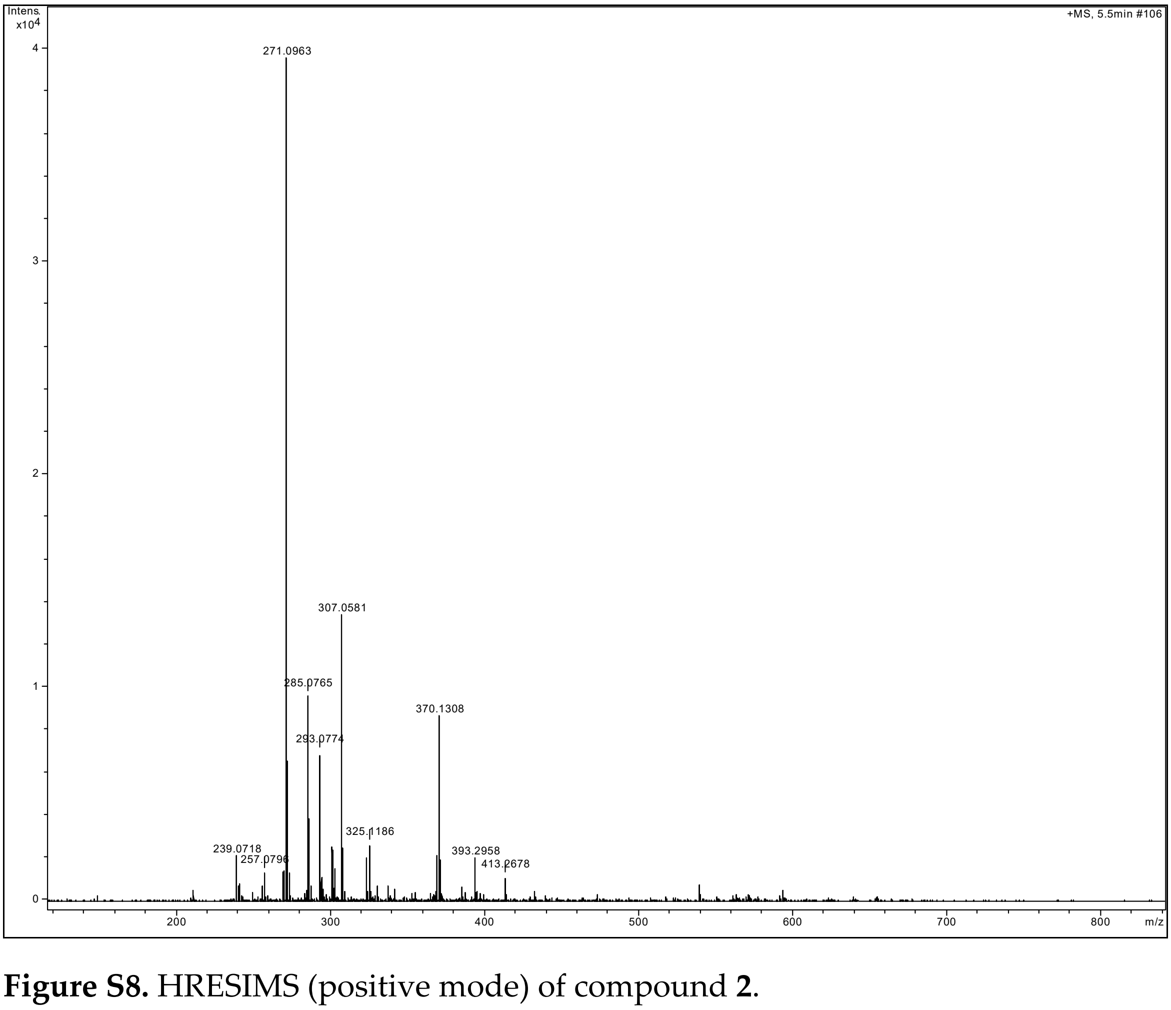

Figure S8. HRESIMS (positive mode) of compound **2**.

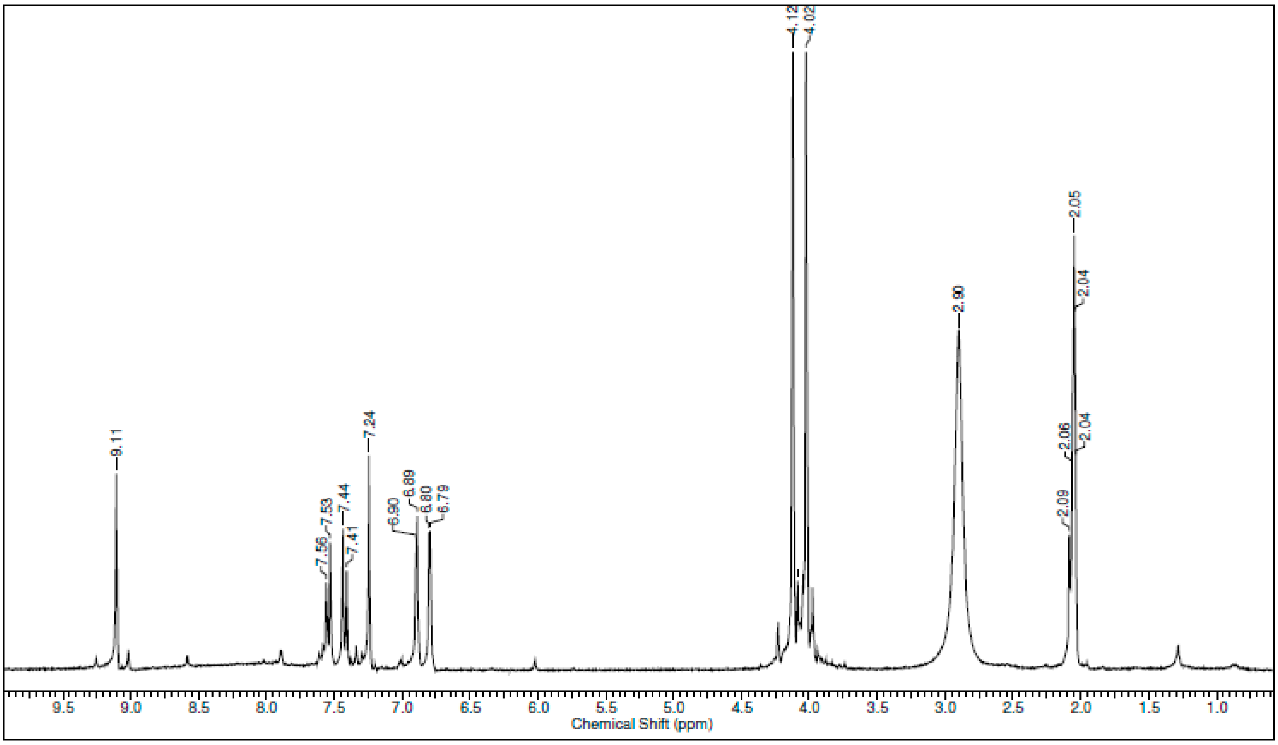

Figure S9. ^{1}H NMR spectrum (300 MHz, acetone-d$_6$) of compound **2**.

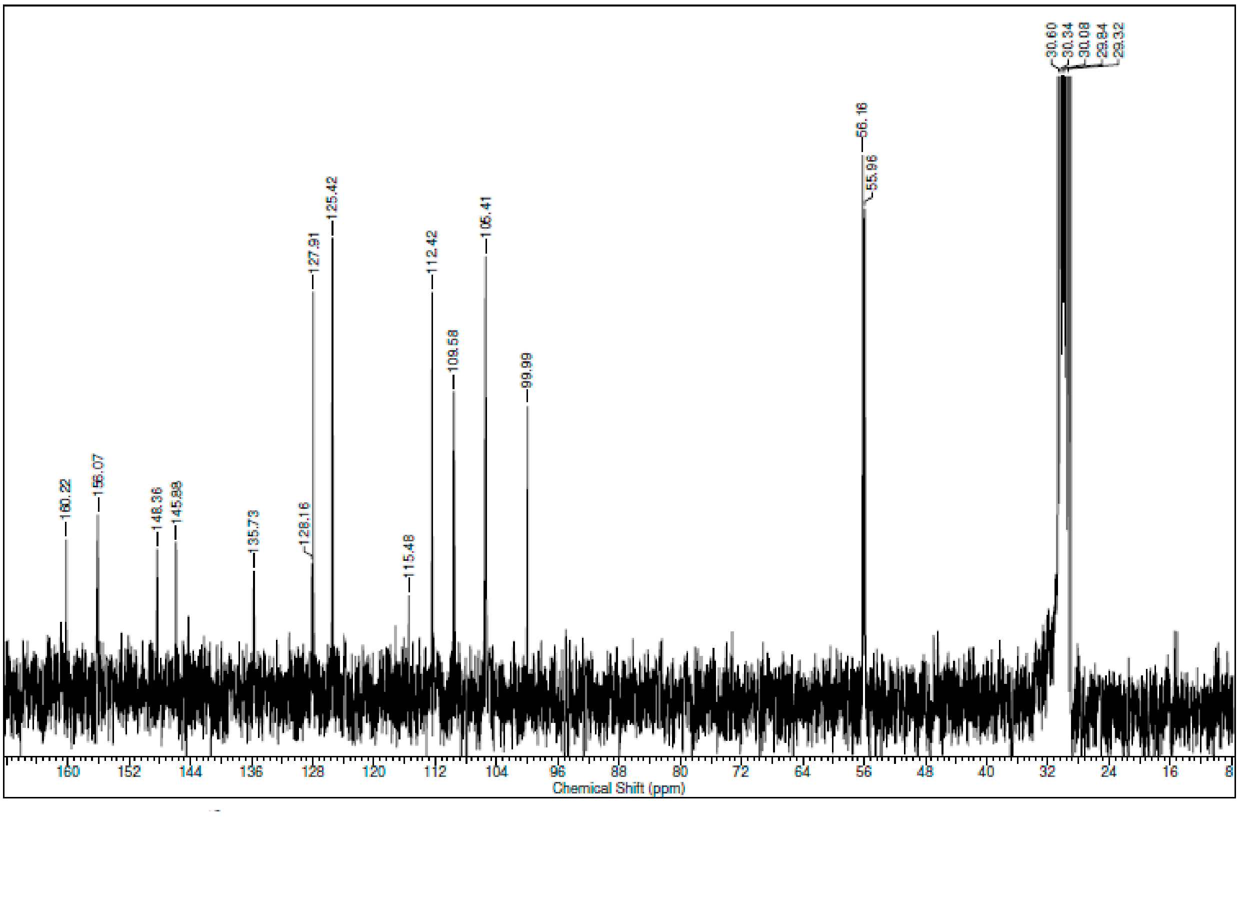

Figure S10. ^{13}C NMR spectrum (75 MHz, acetone-d_6) of compound **2**.

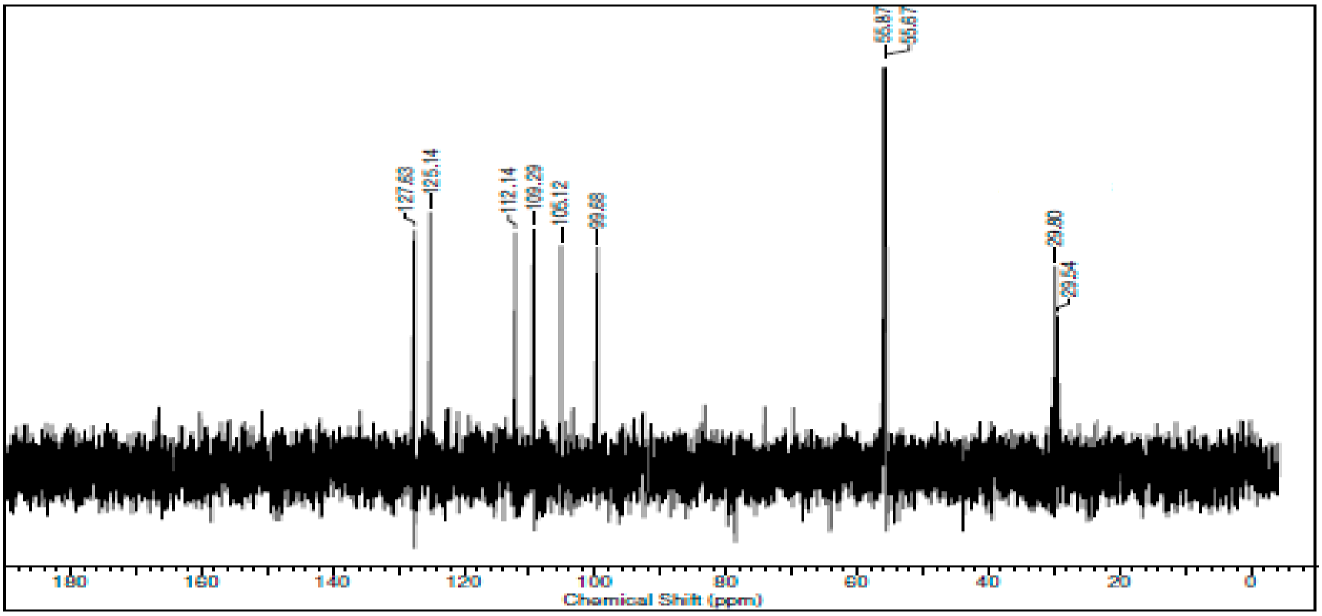

Figure S11. DEPT-135 spectrum (acetone-d_6) of compound **2**.

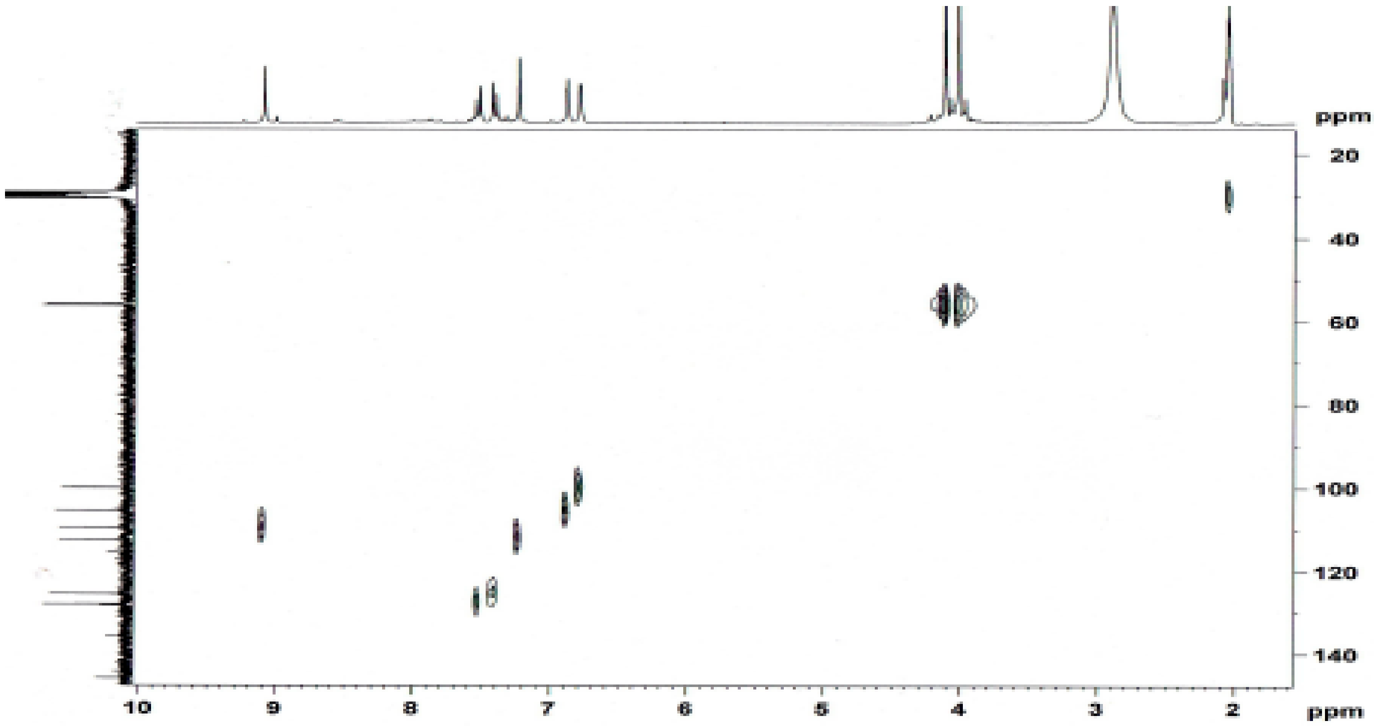

Figure S12. HSQC spectrum (acetone-d_6) of compound **2**.

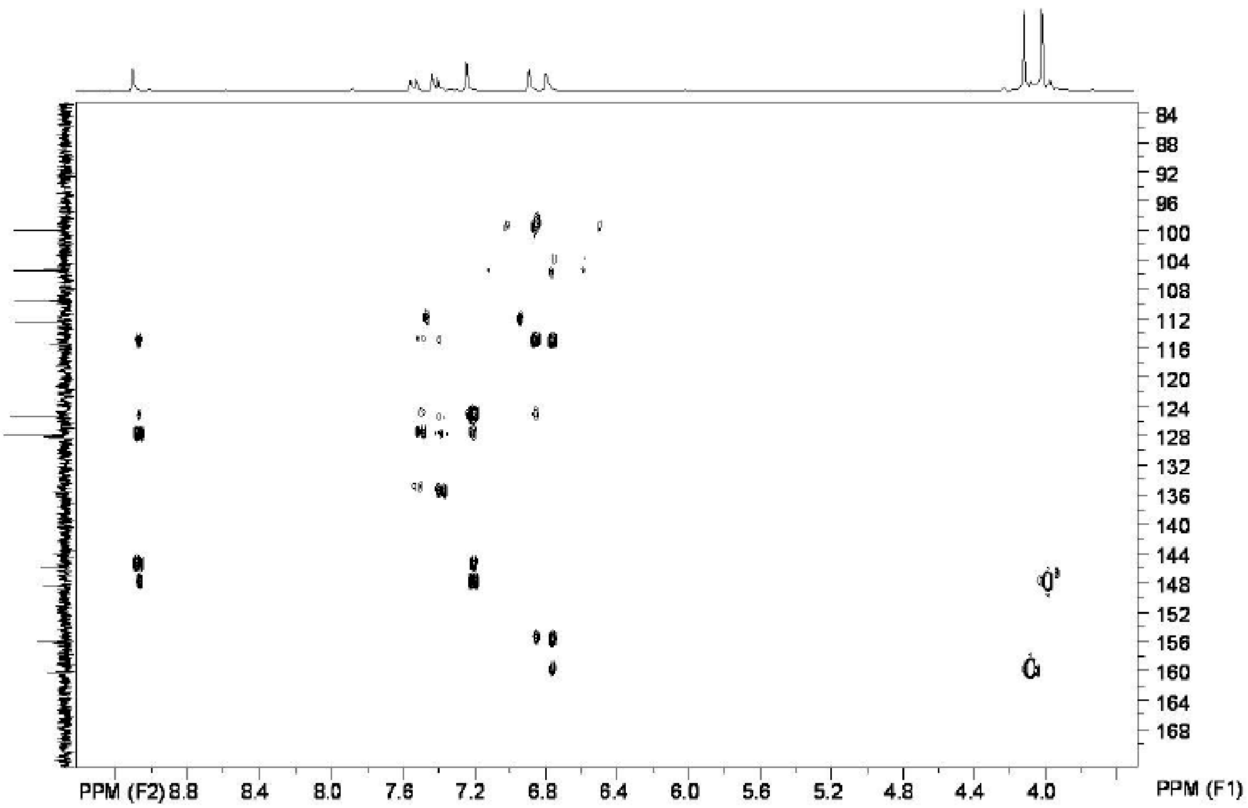

Figure S13. HMBC spectrum (acetone-d₆) of compound **2**.

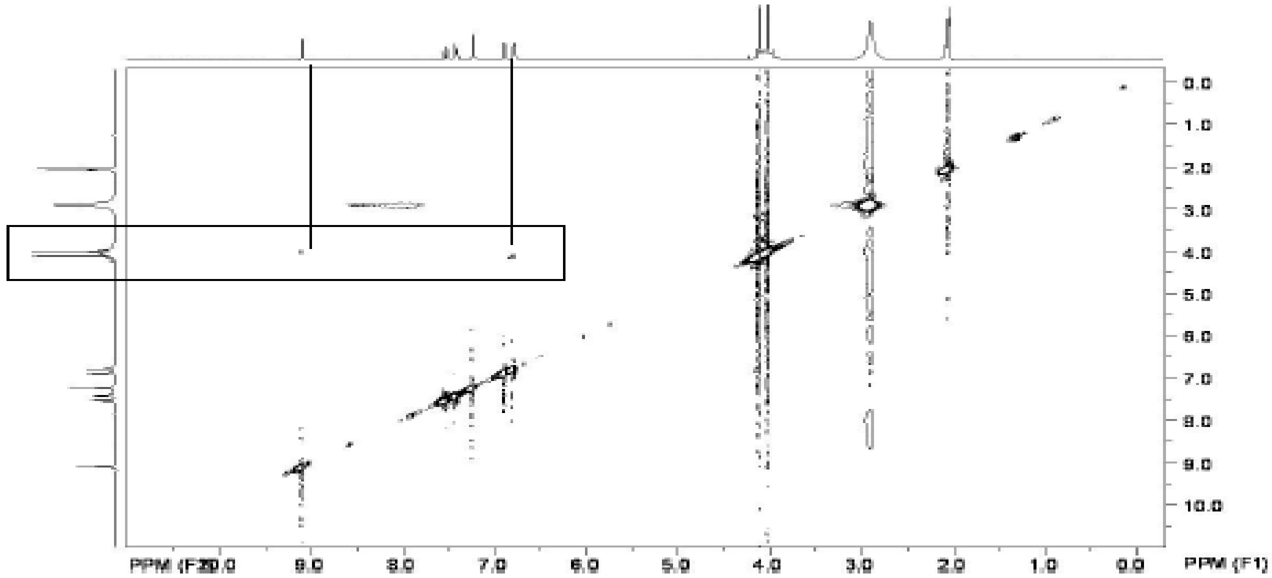

Figure S14. NOESY spectrum (300 MHz, acetone-d₆) of compound **2**.

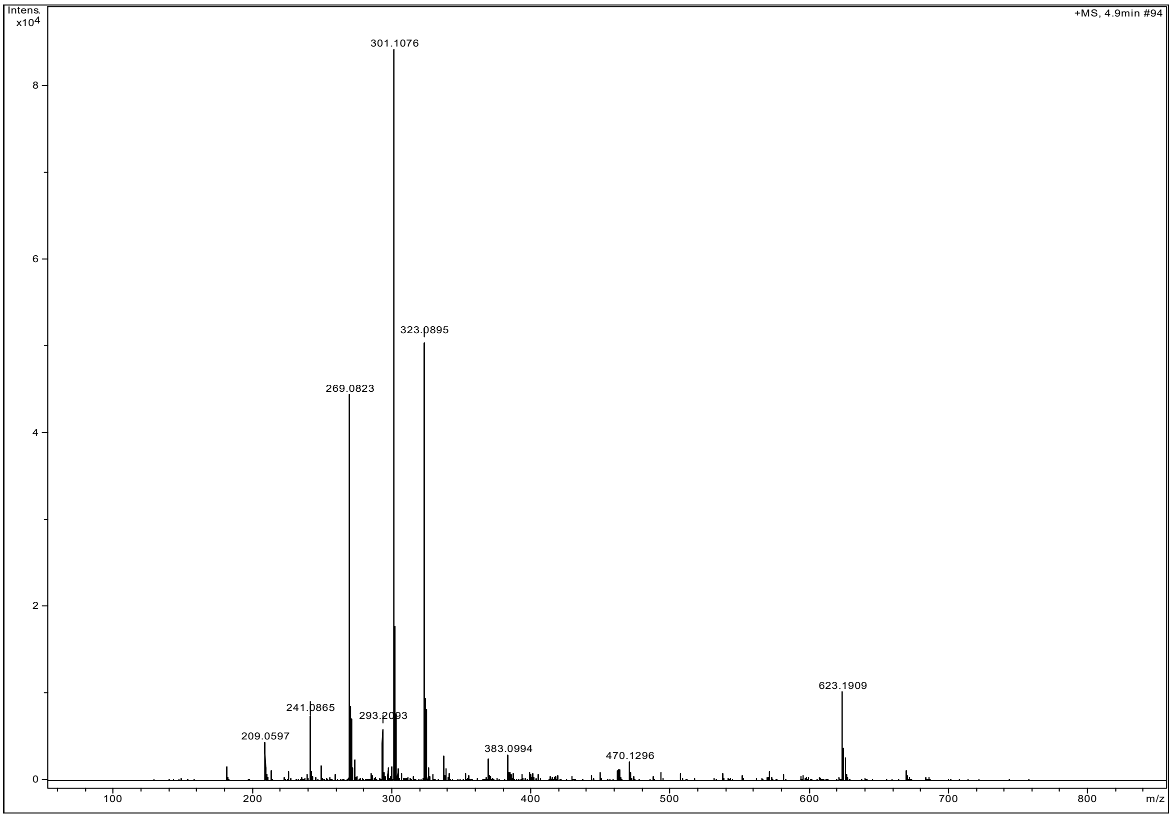

Figure S15. HRESIMS (positive mode) of compound **3**.

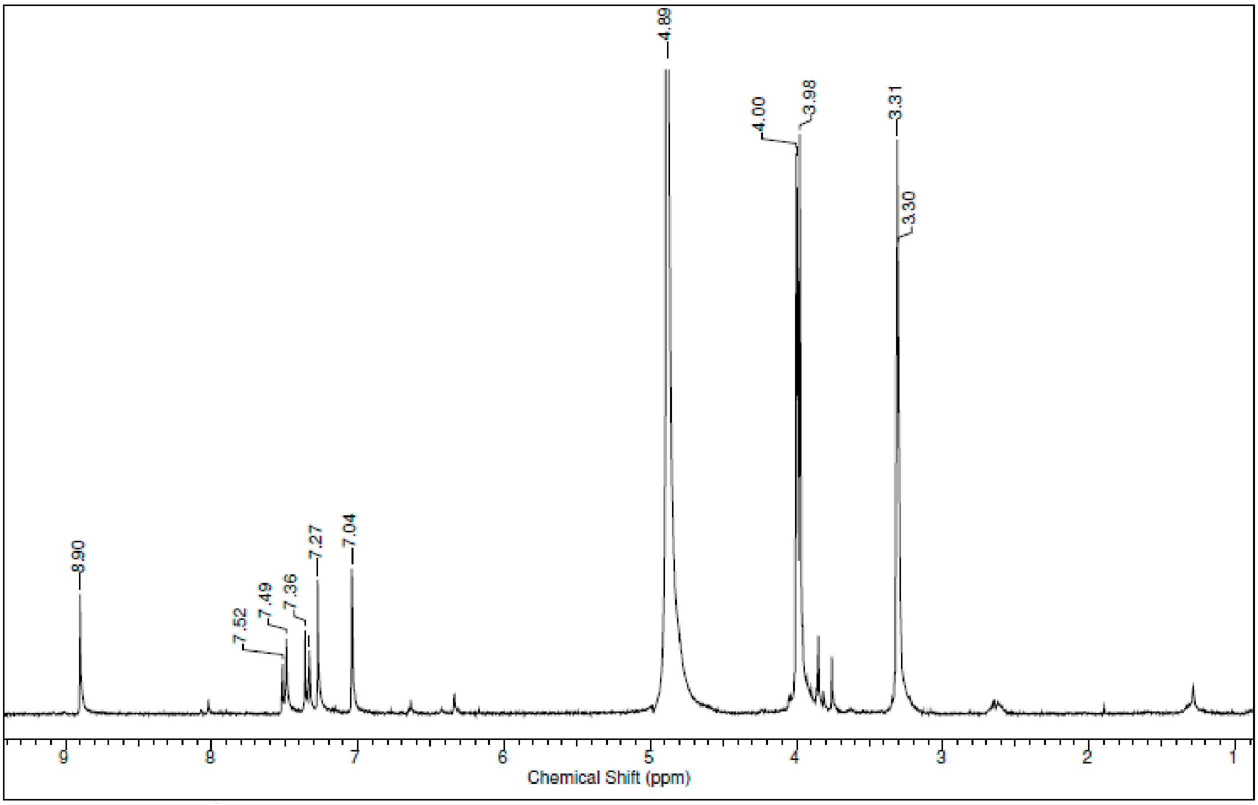

Figure S16. ^{1}H NMR spectrum (300 MHz, CD_3OD) of compound **3**.

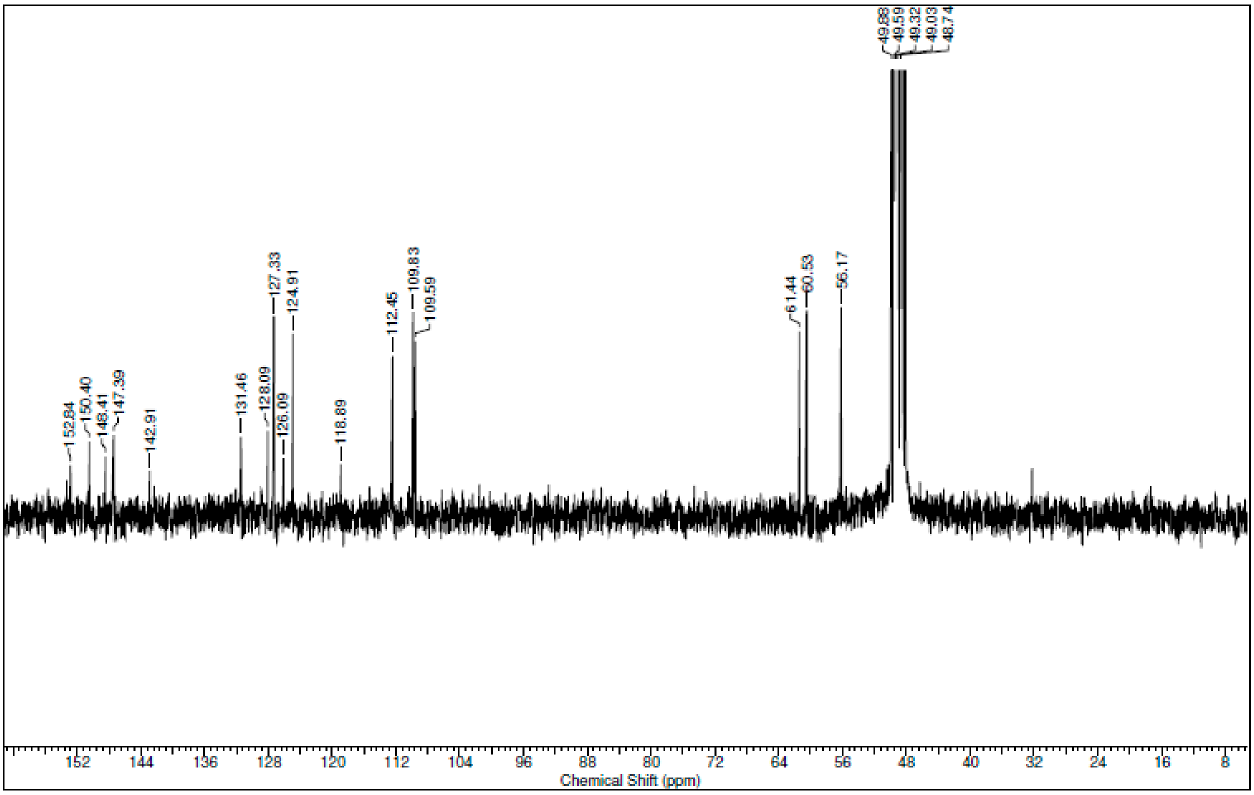

Figure S17. ^{13}C NMR spectrum (75 MHz, CD_3OD) of compound **3**.

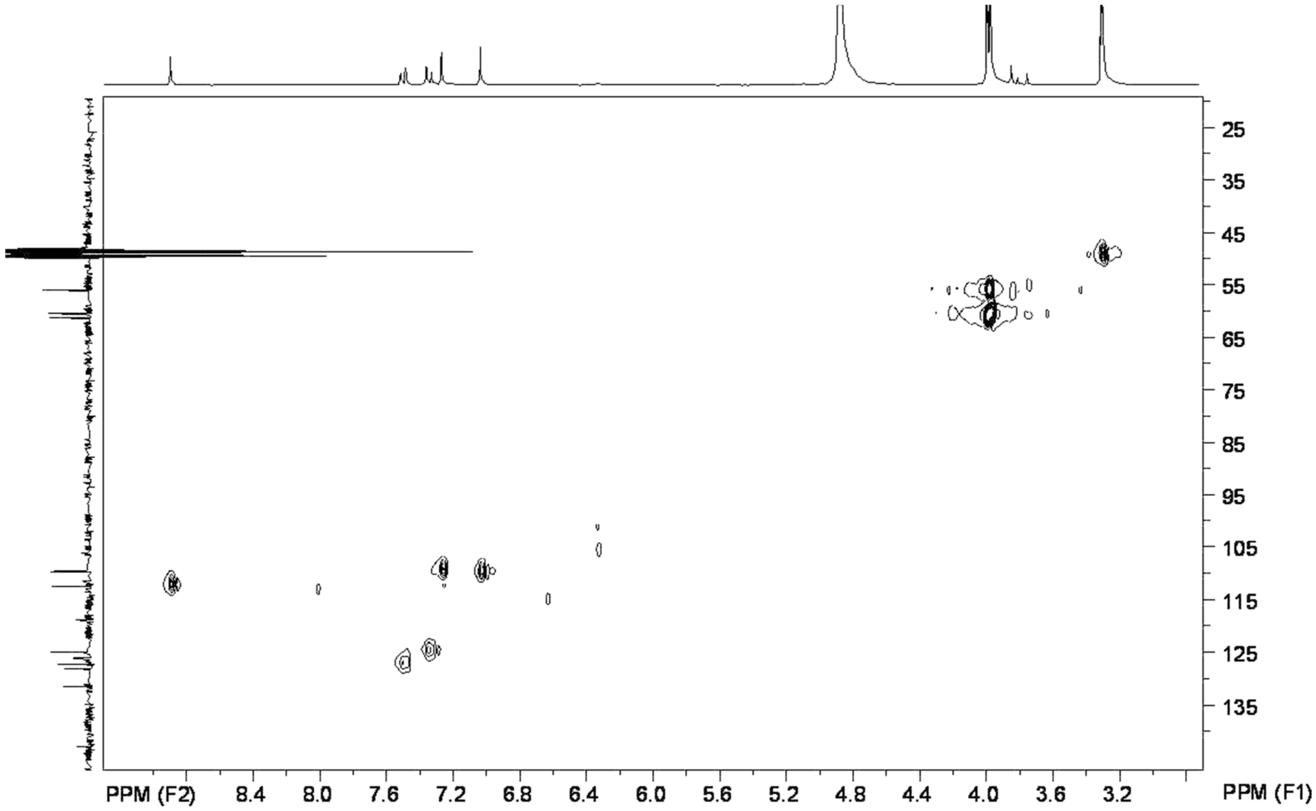

Figure S18. HSQC spectrum (CD_3OD) of compound **3**.

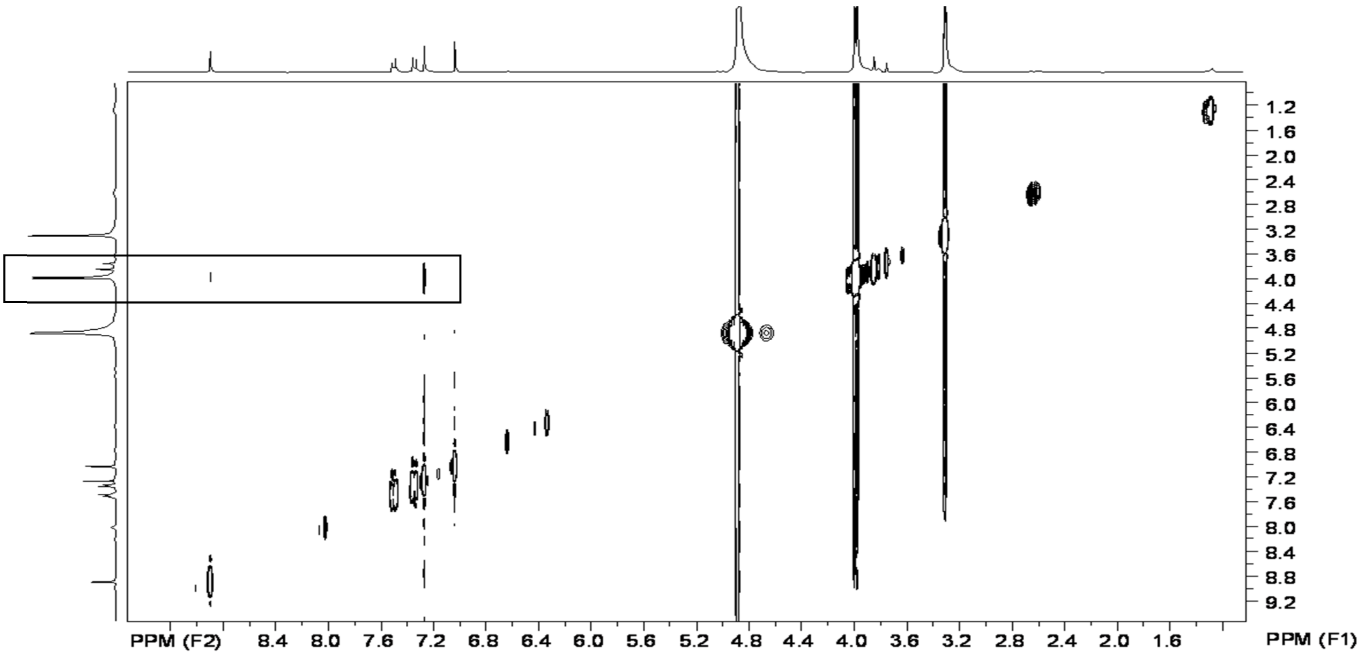

Figure S19. NOESY spectrum (300 MHz, CD_3OD) of compound **3**.

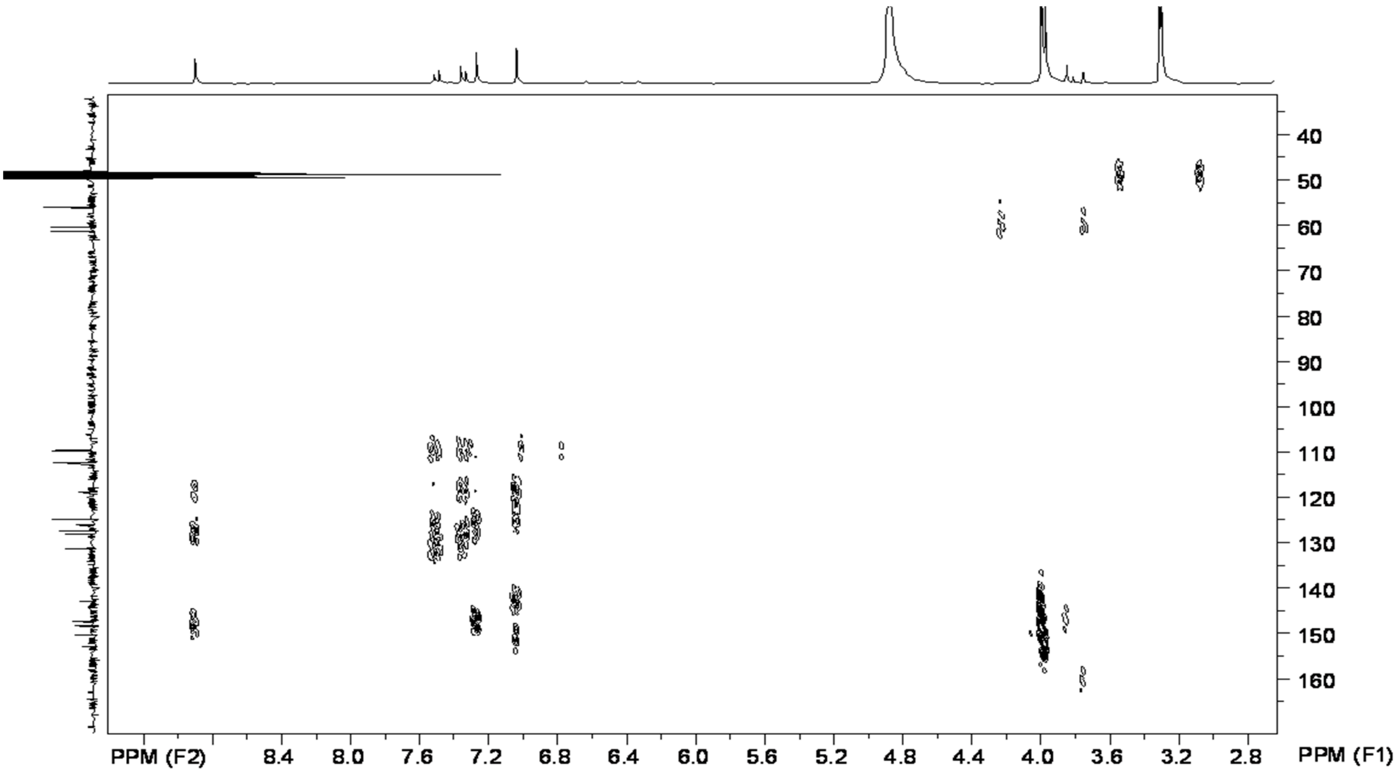

Figure S20. HMBC spectrum (CD_3OD) of compound **3**.

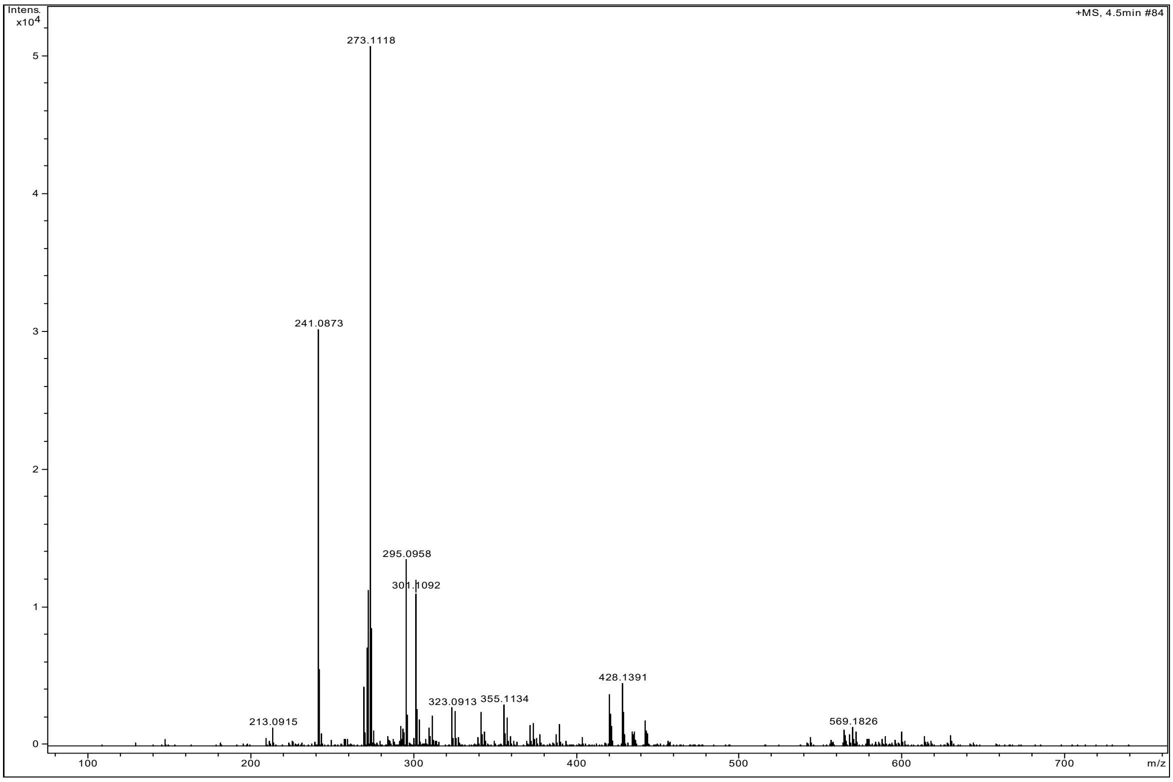

Figure S21. HRESIMS (positive mode) of compound **4**.

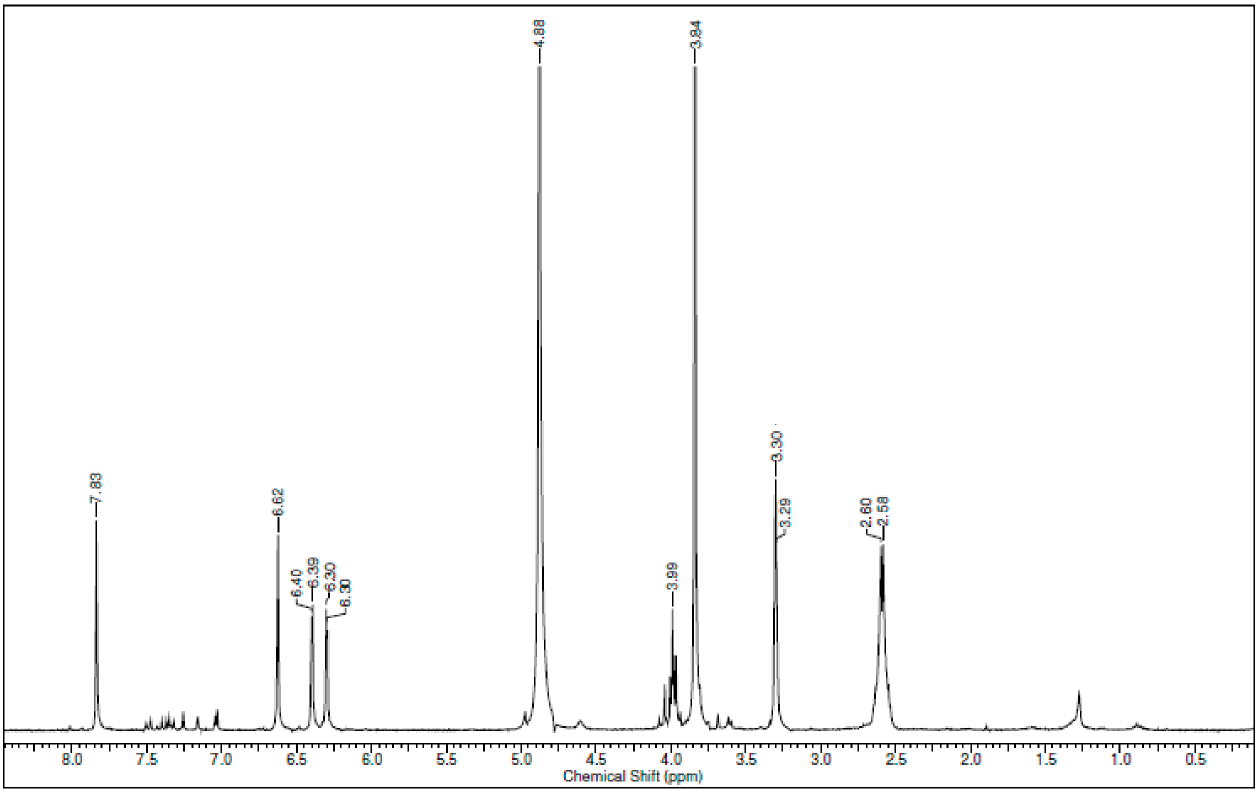

Figure S22. ^{1}H NMR spectrum (300 MHz, CD_3OD) of compound **4**.

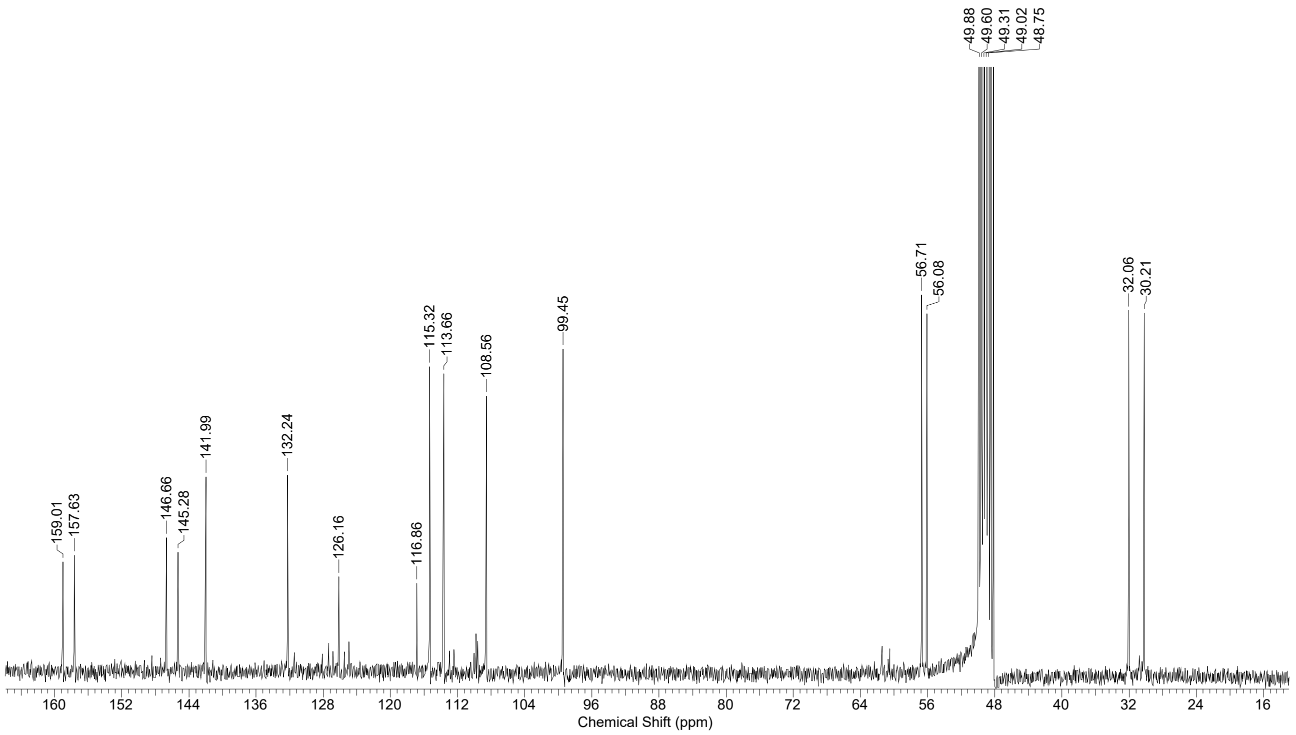

Figure S23 ^{13}C NMR spectrum (75 MHz, CD_3OD) of compound **4**.

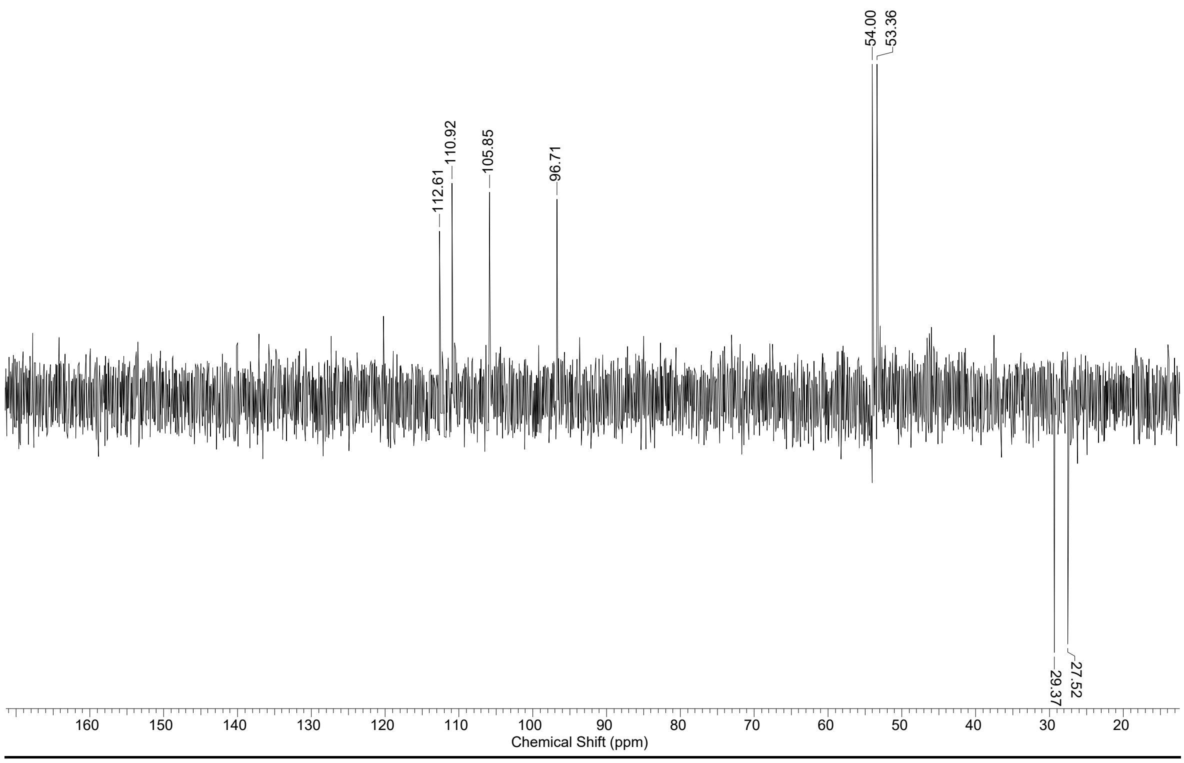

Figure S24. DEPT-135 spectrum (CD_3OD) of compound **4**.

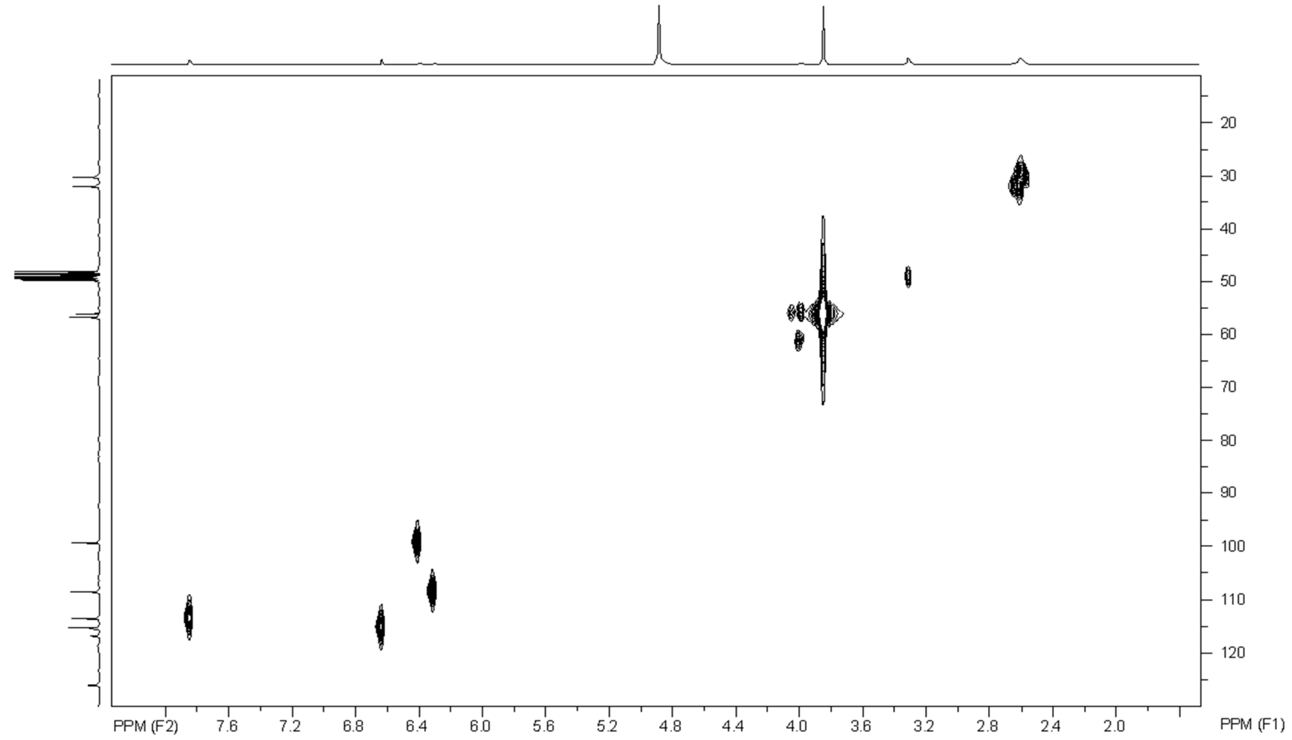

Figure S25. HSQC spectrum (CD_3OD) of compound **4**.

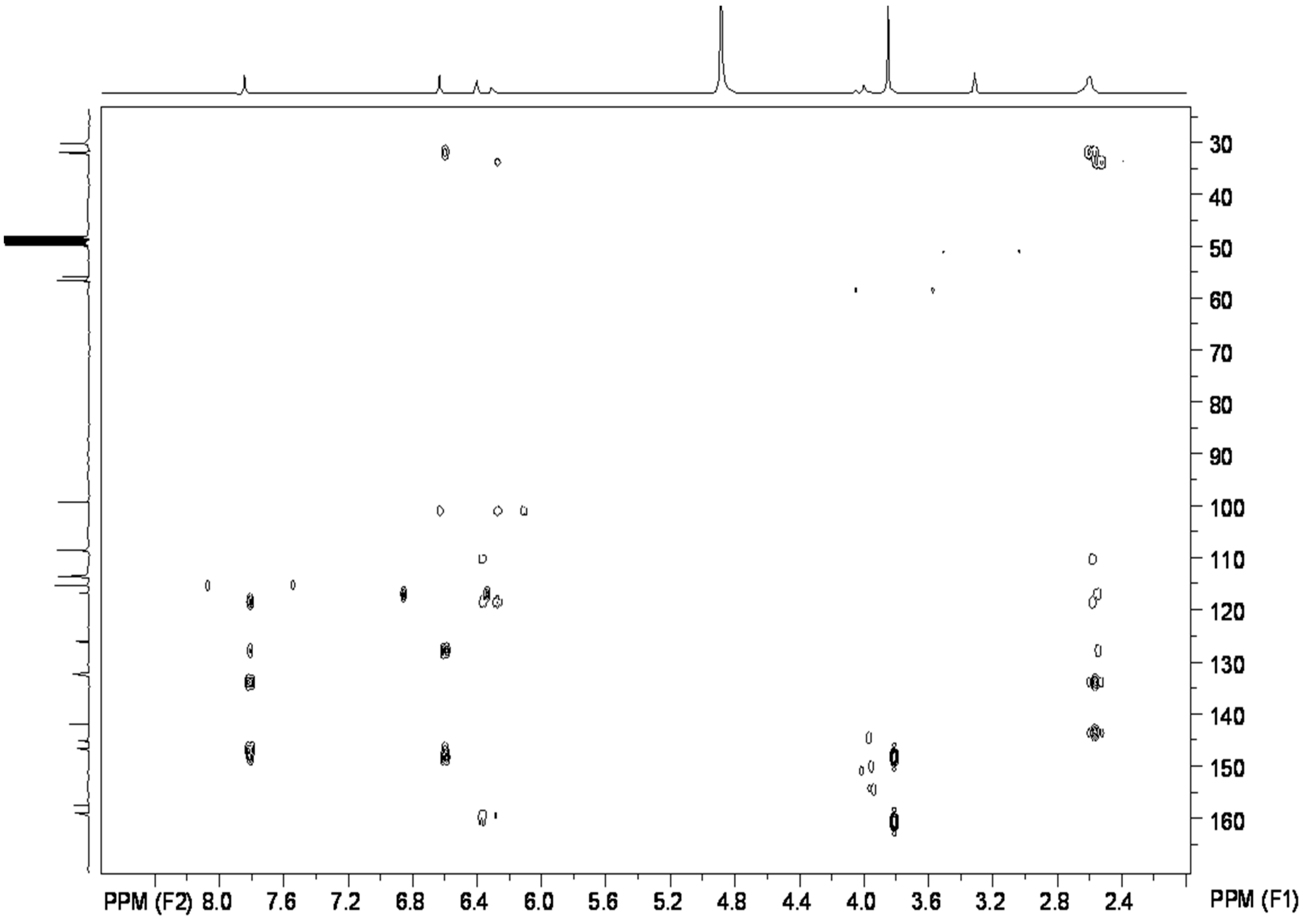

Figure S26. HMBC spectrum (CD_3OD) of compound **4**.

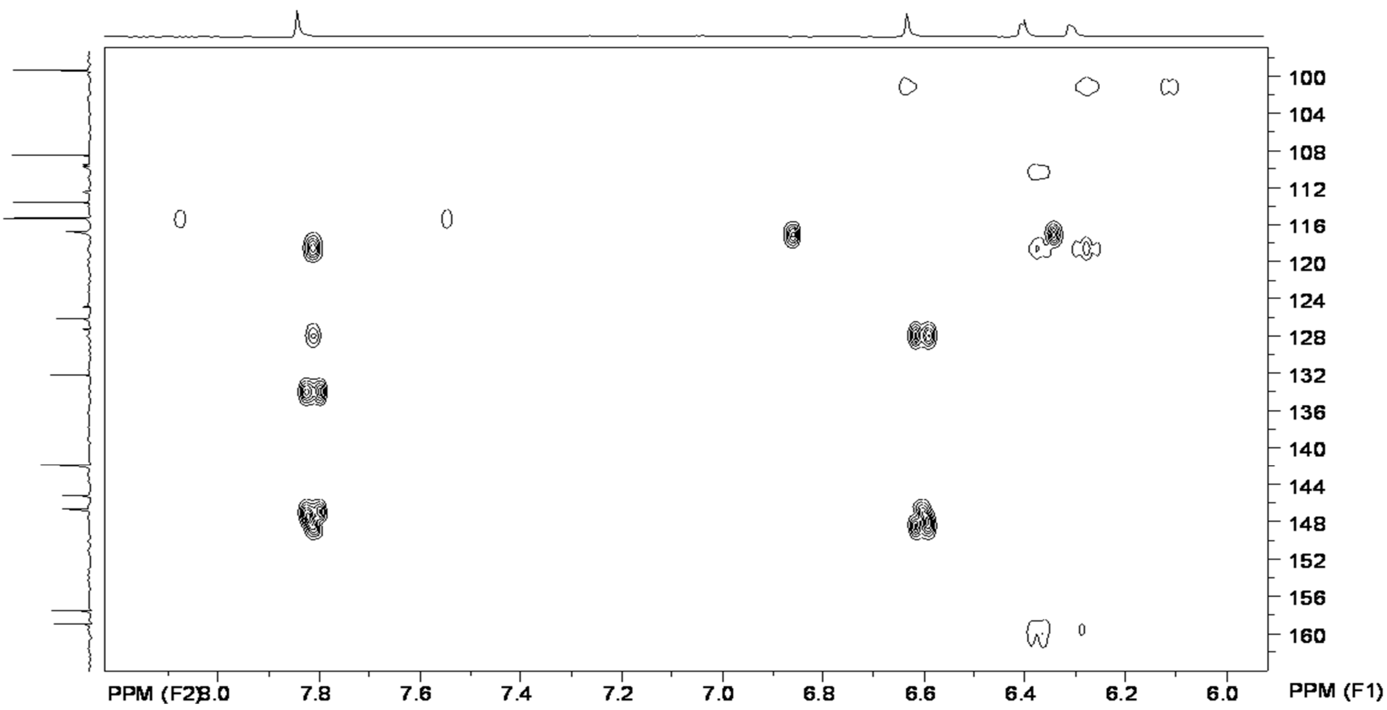

Figure S27. HMBC spectrum (expansion) of compound **4**.

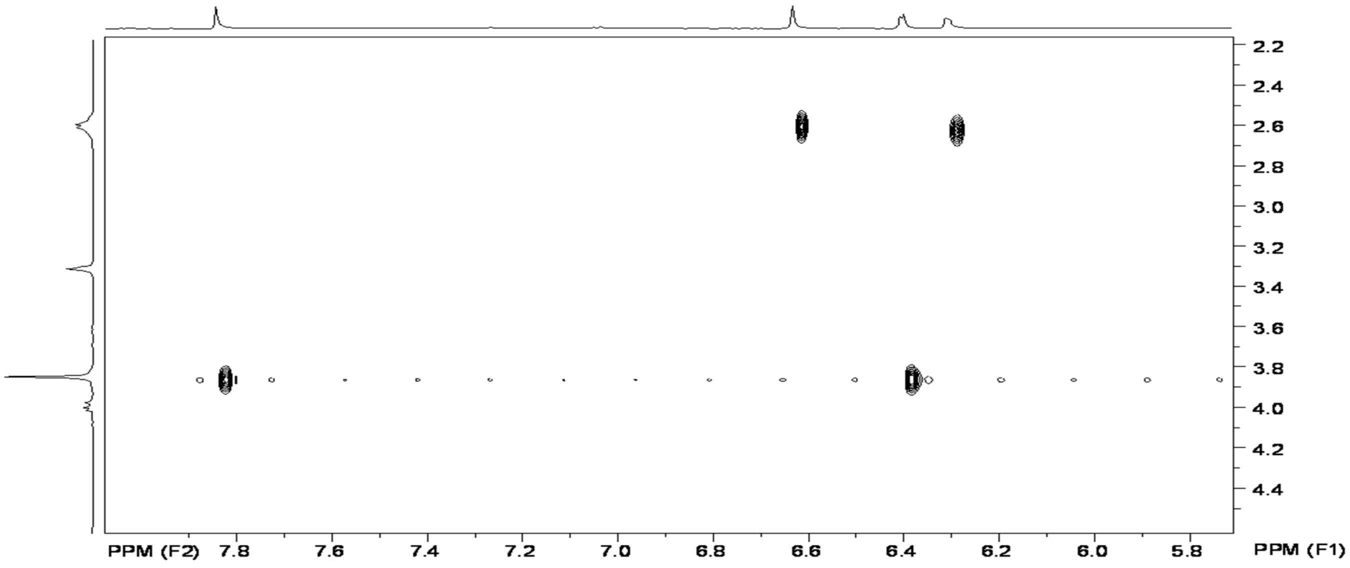

Figure S28. NOESY spectrum (300 MHz, CD_3OD) of compound **4**.

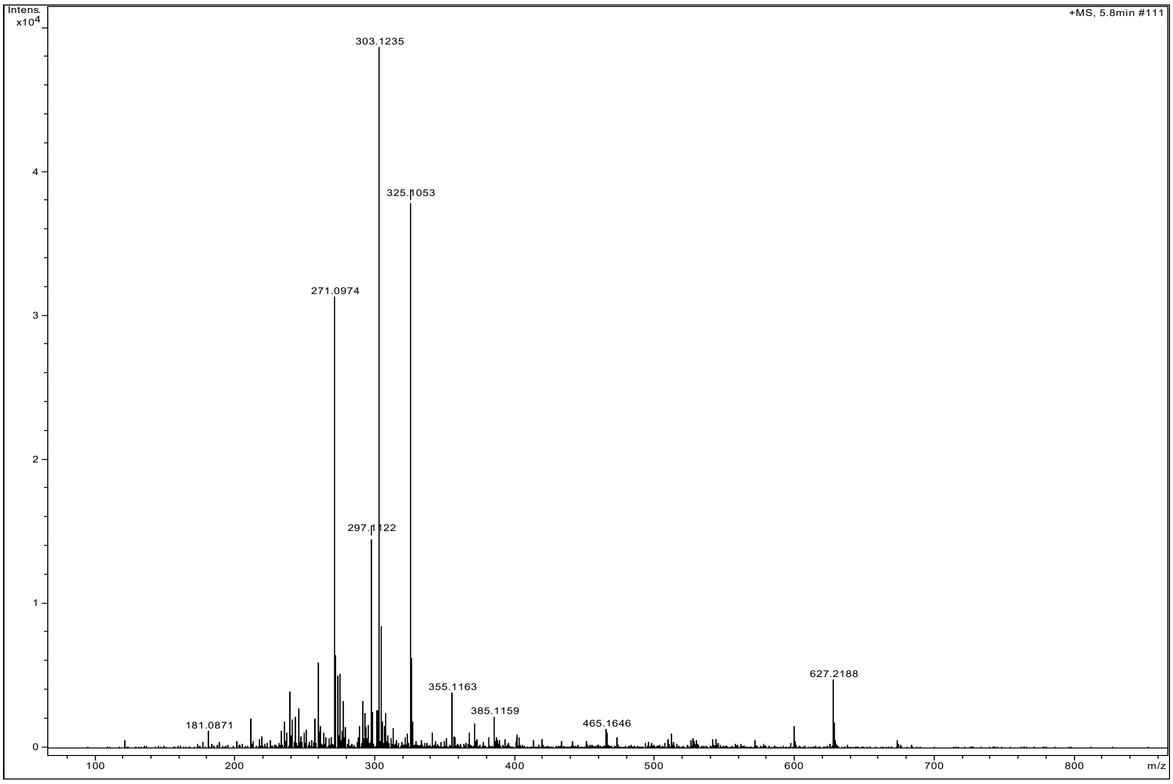

Figure S29. HRESIMS (positive mode) of compound **5**.

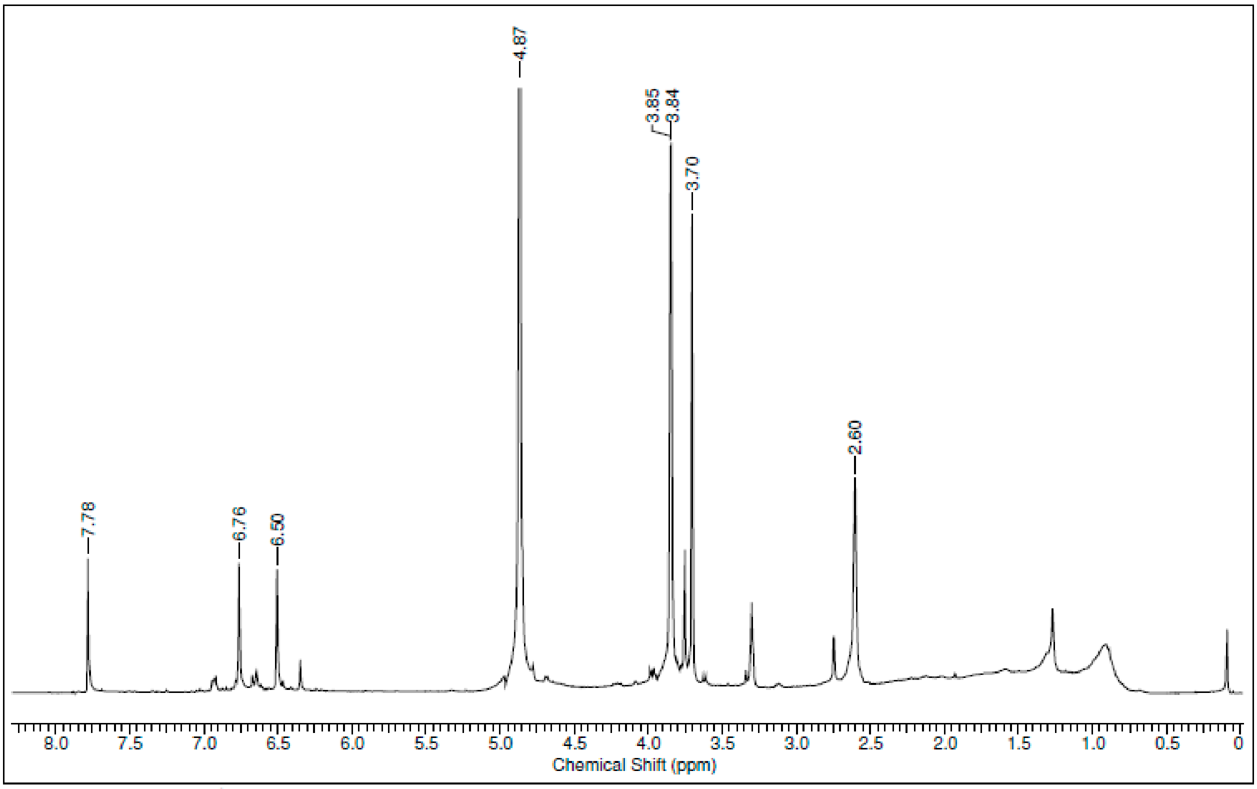

Figure S30. ^{1}H NMR spectrum (300 MHz, CD_3OD) of compound **5**.

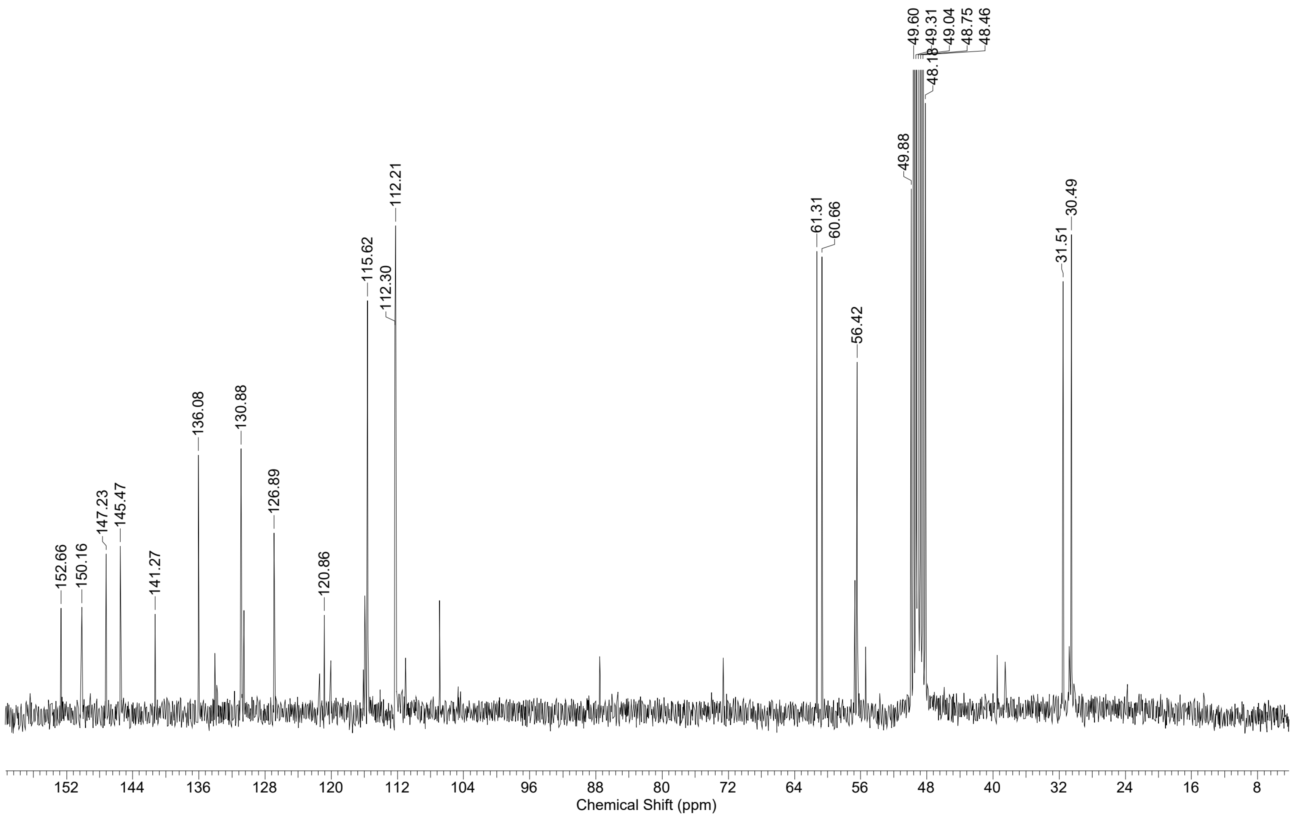

Figure S31. ^{13}C NMR spectrum (75 MHz, CD_3OD) of compound **5**.

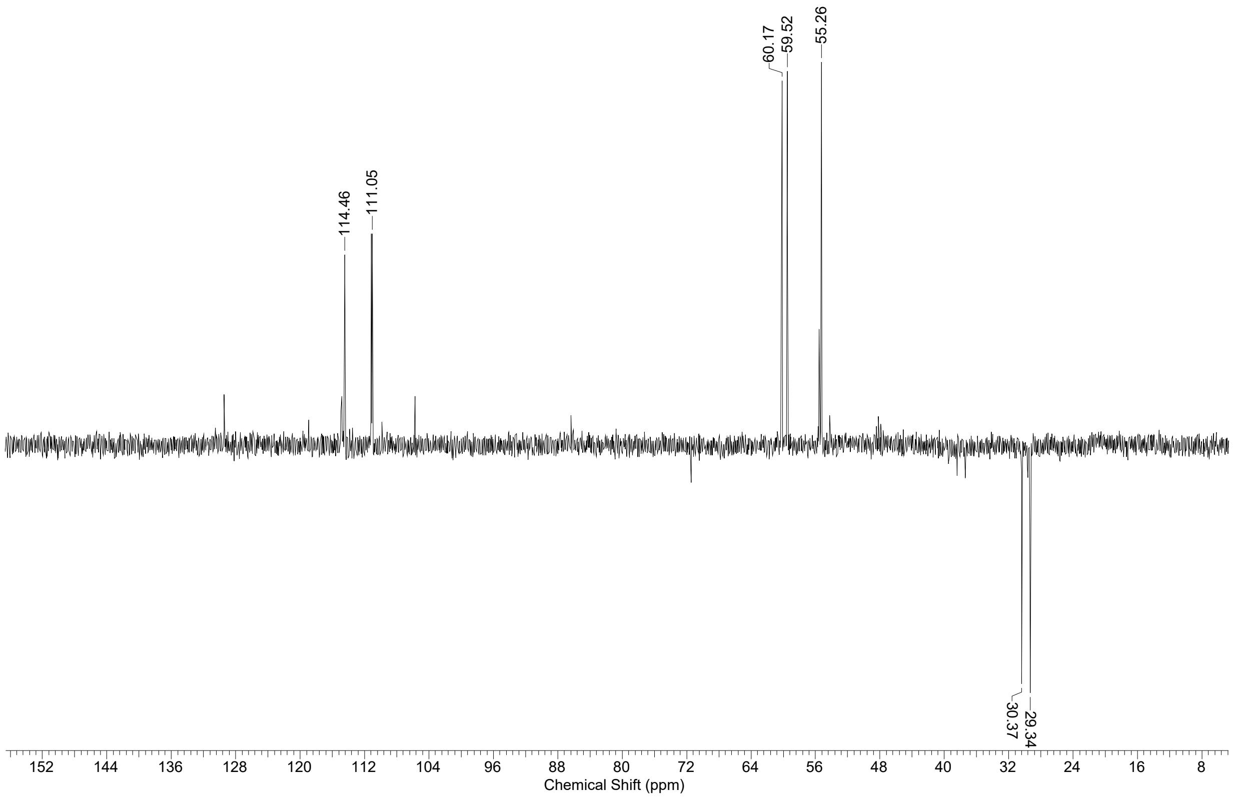

Figure S32. DEPT-135 spectrum (CD_3OD) of compound **5**.

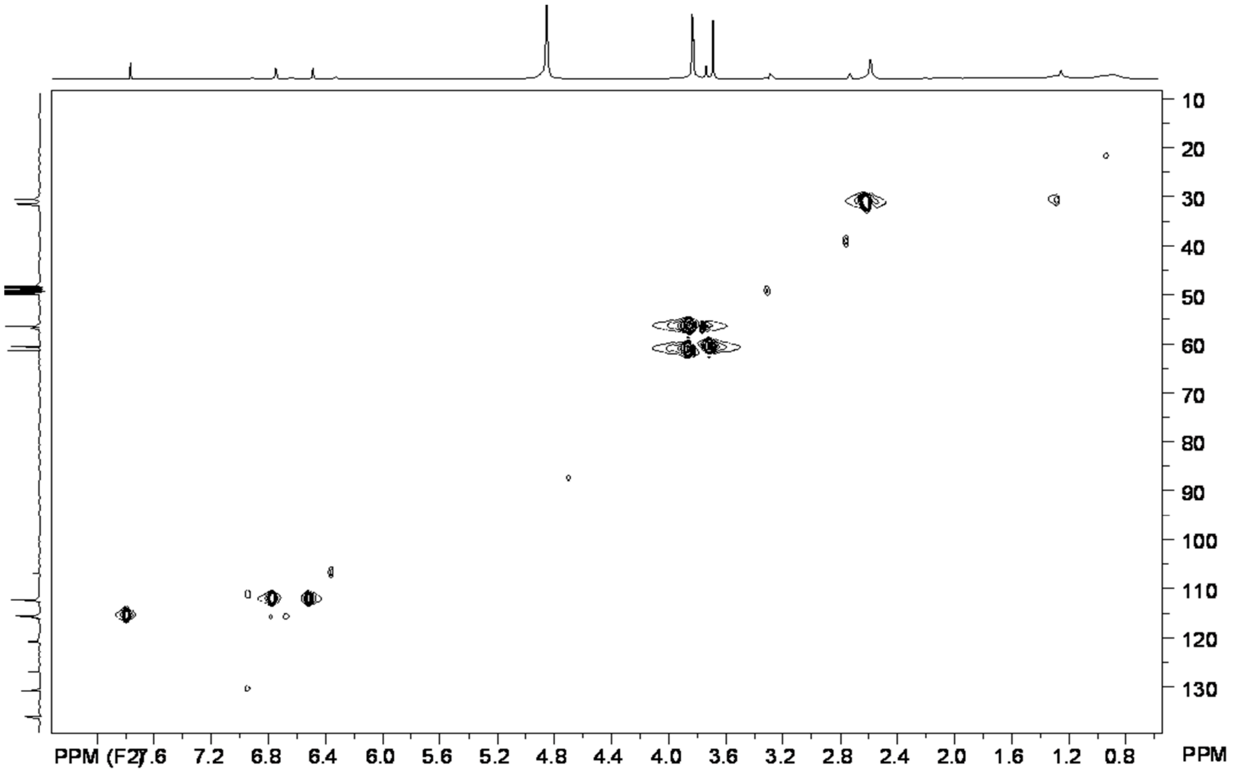

Figure S33. HSQC spectrum (CD_3OD) of compound **5**.

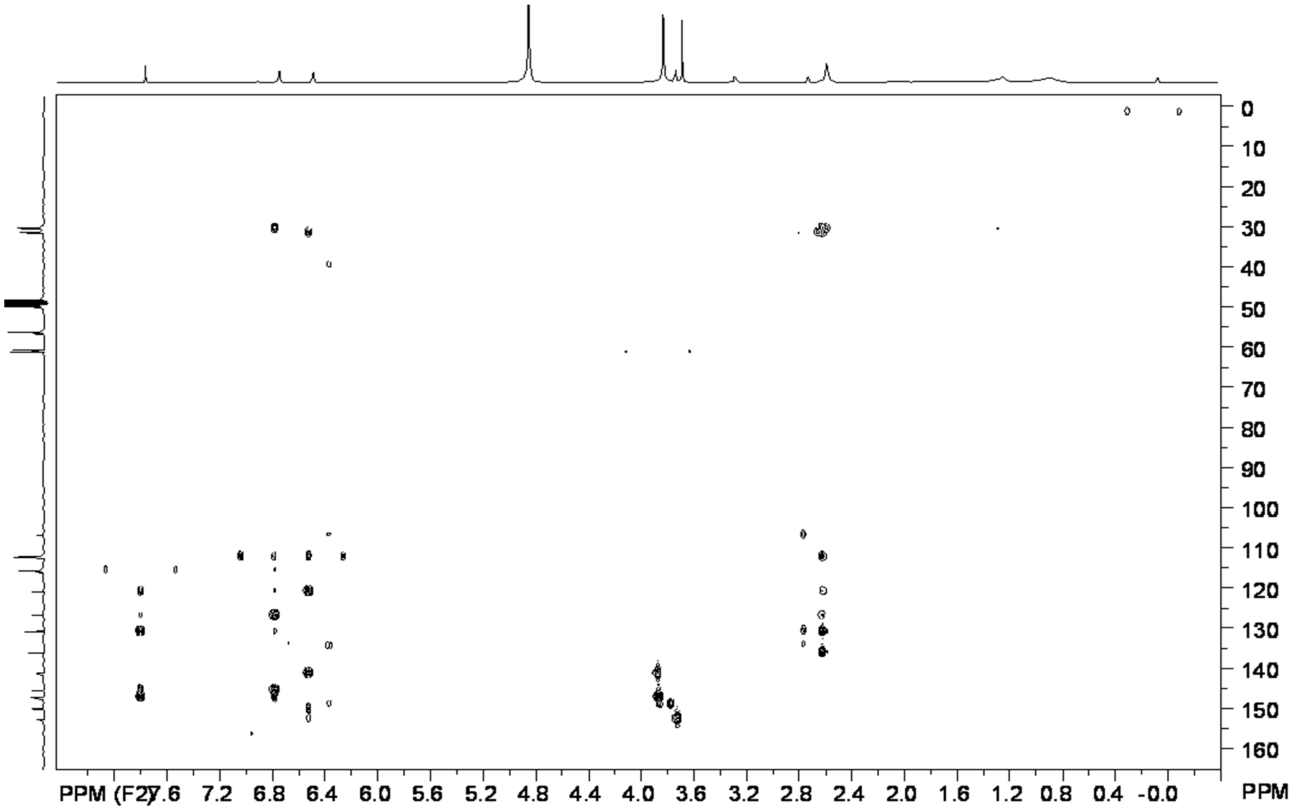

Figure S34. HMBC spectrum (CD_3OD) of compound **5**.

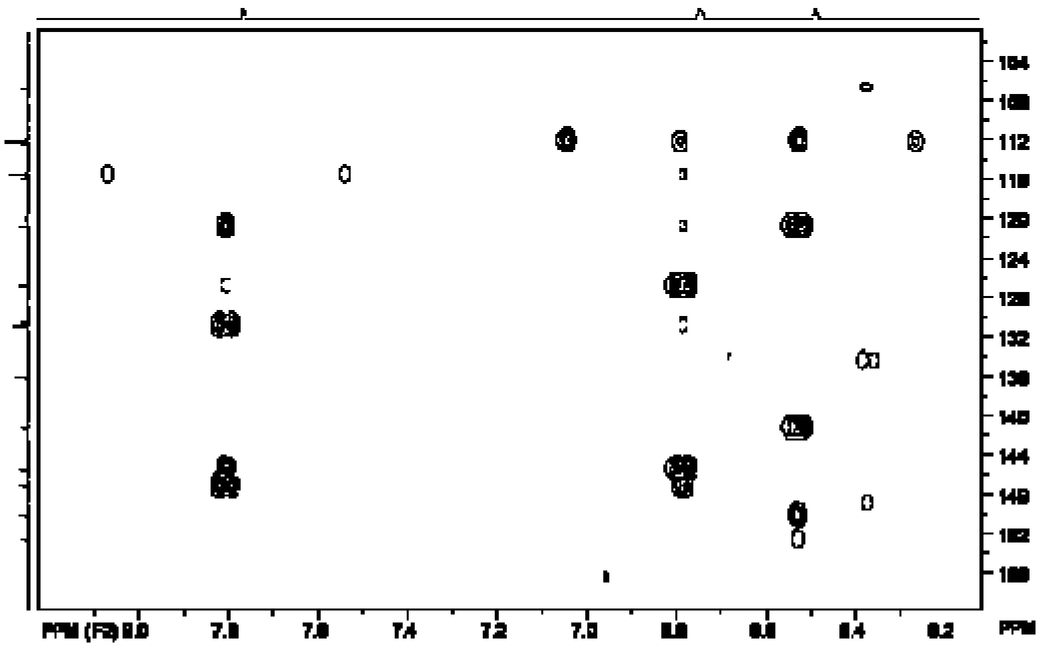

Figure S35. HMBC spectrum (expansion) of compound **5**.

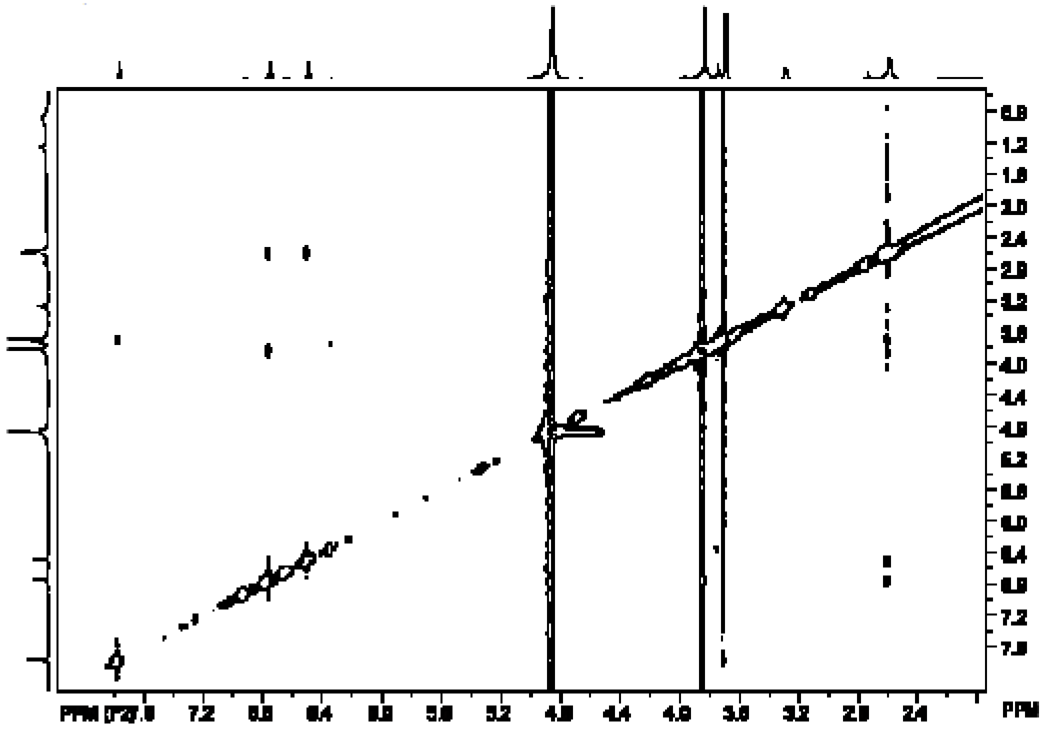

Figure S36. NOESY spectrum (300 MHz, CD_3OD) of compound **5**.

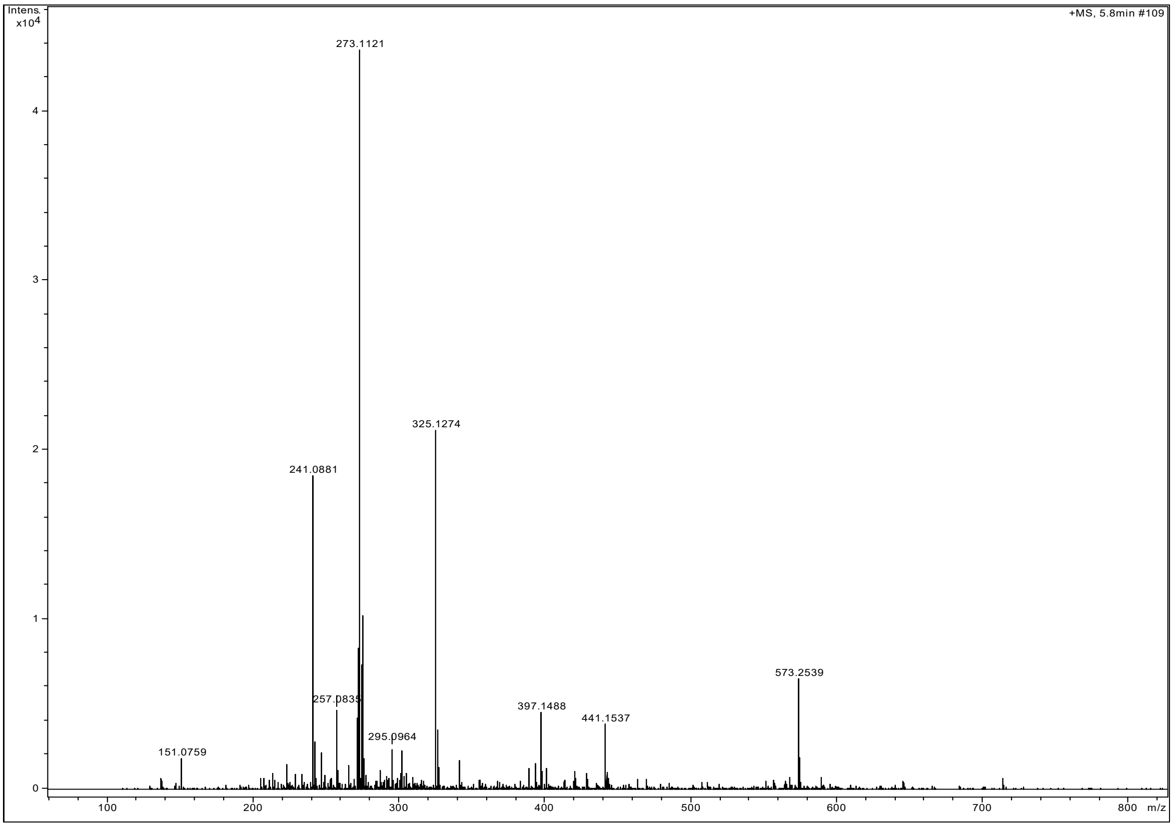

Figure S37. HRESIMS (positive mode) of compound **6**.

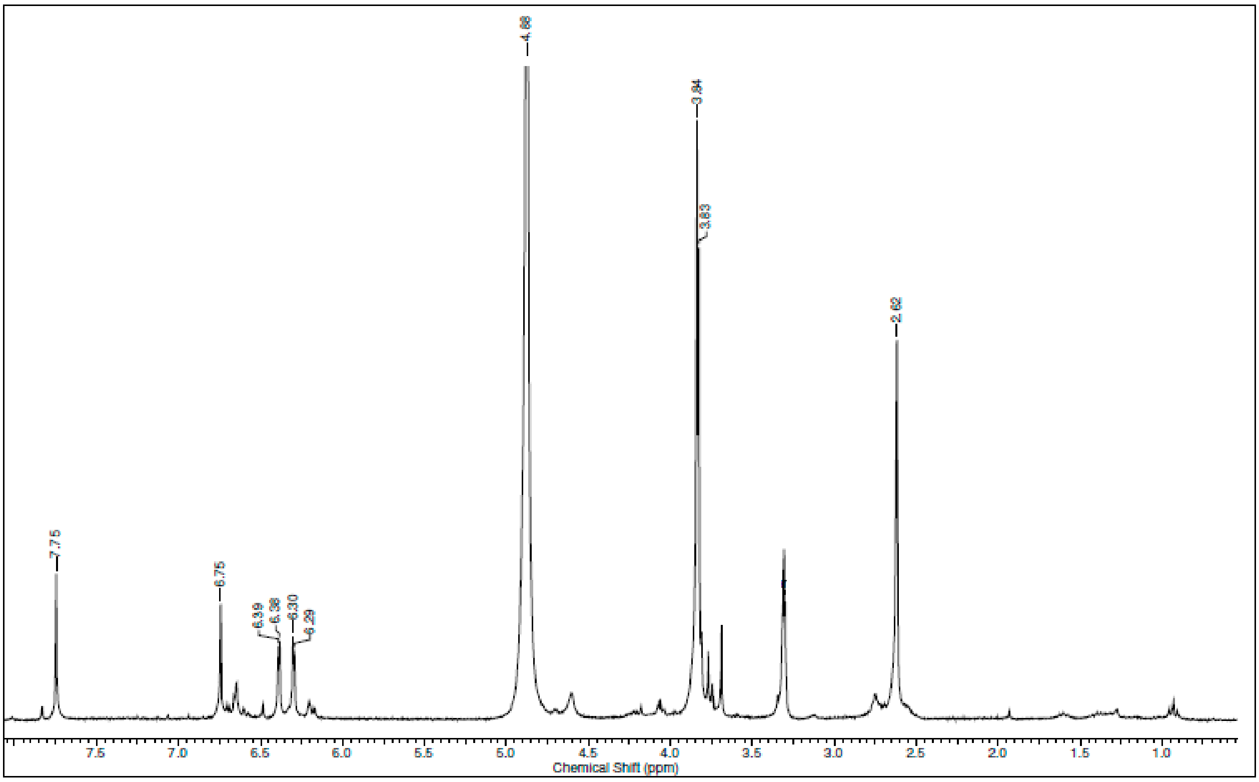

Figure S38. ^{1}H NMR spectrum (300 MHz, CD_3OD) of compound **6**.

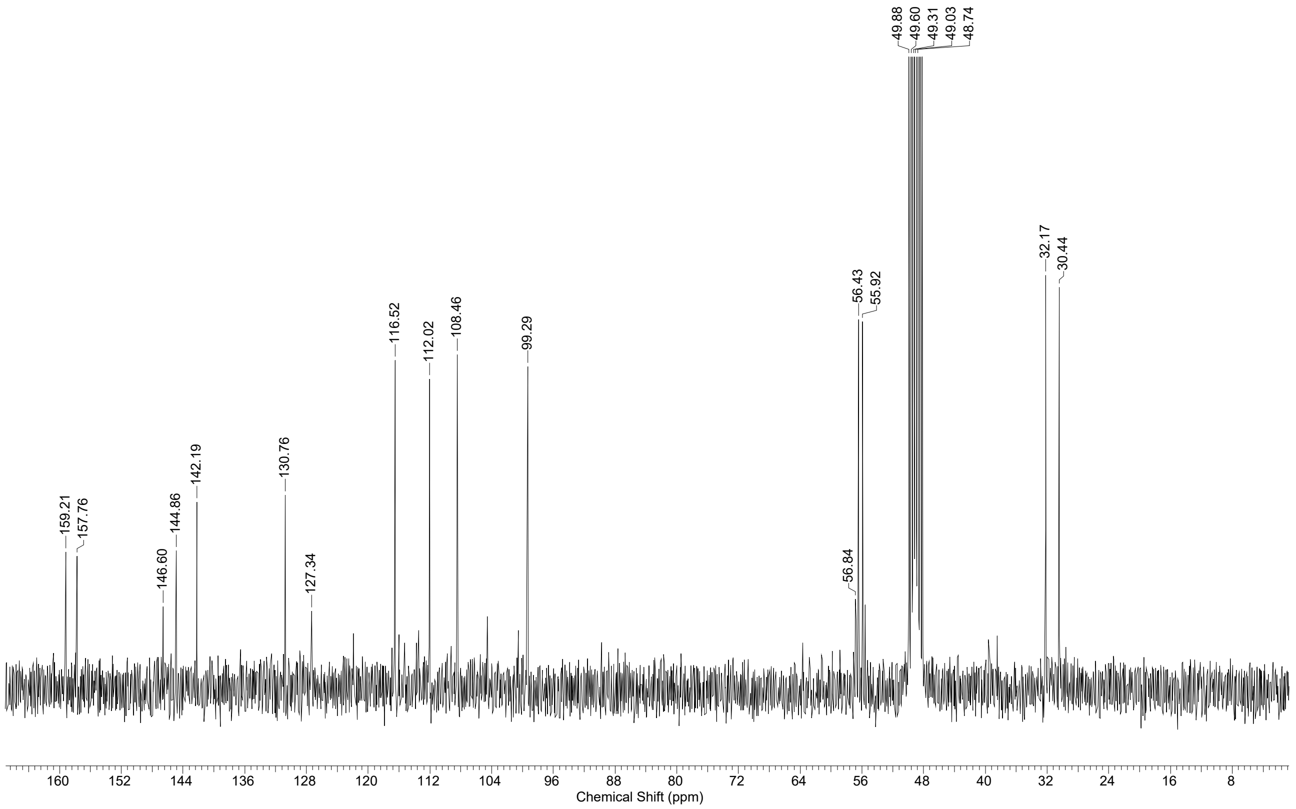

Figure S39. ^{13}C NMR spectrum (75 MHz, CD_3OD) of compound **6**.

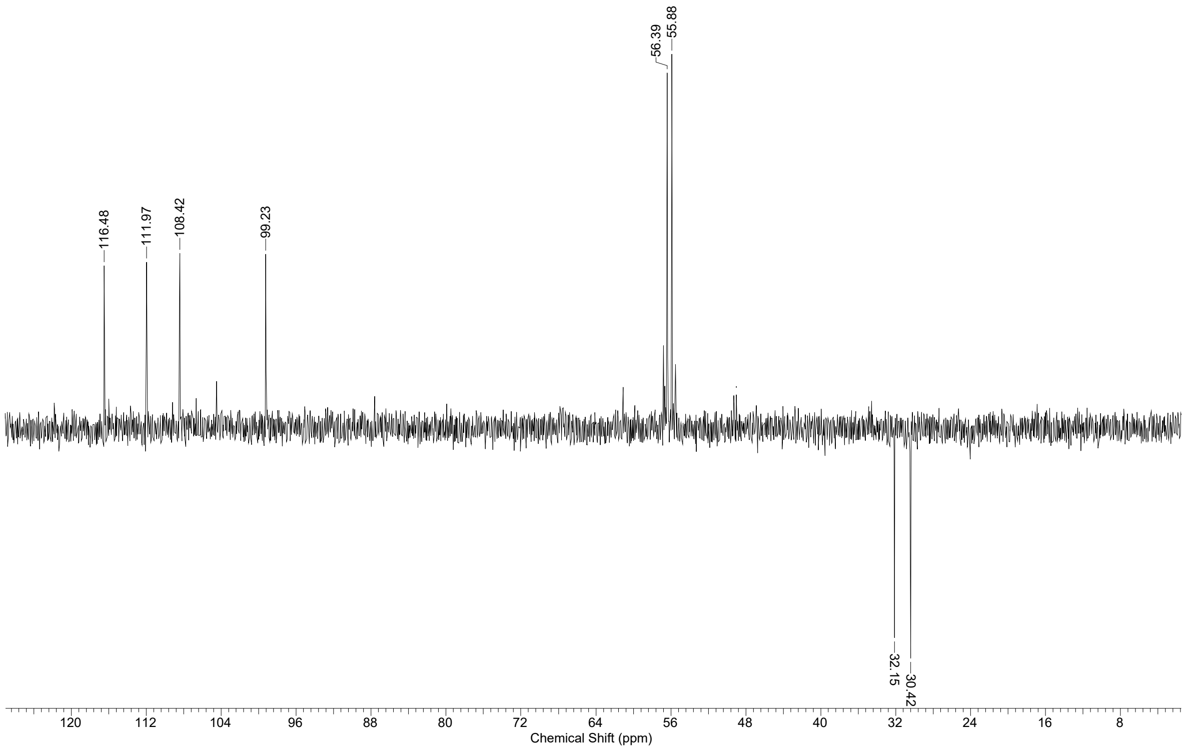

Figure S40. DEPT-135 spectrum (CD_3OD) of compound **6**.

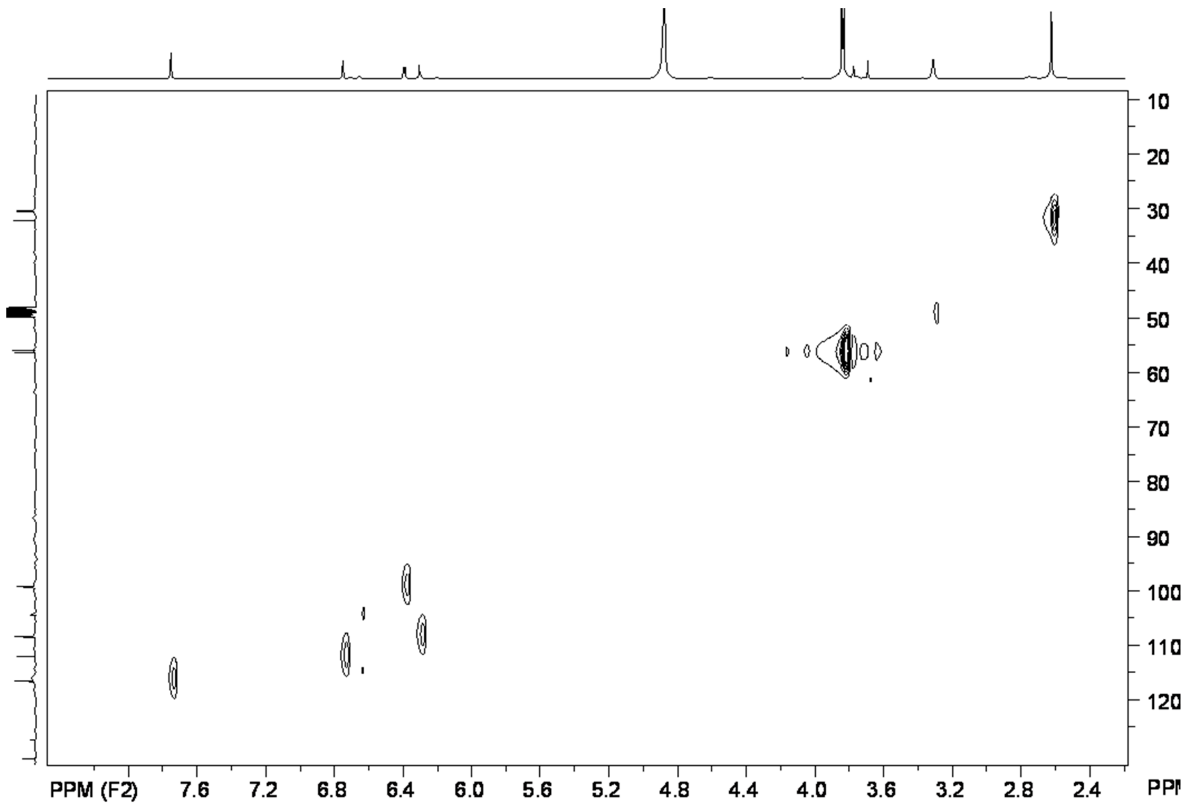

Figure S41. HSQC spectrum (CD_3OD) of compound **6**.

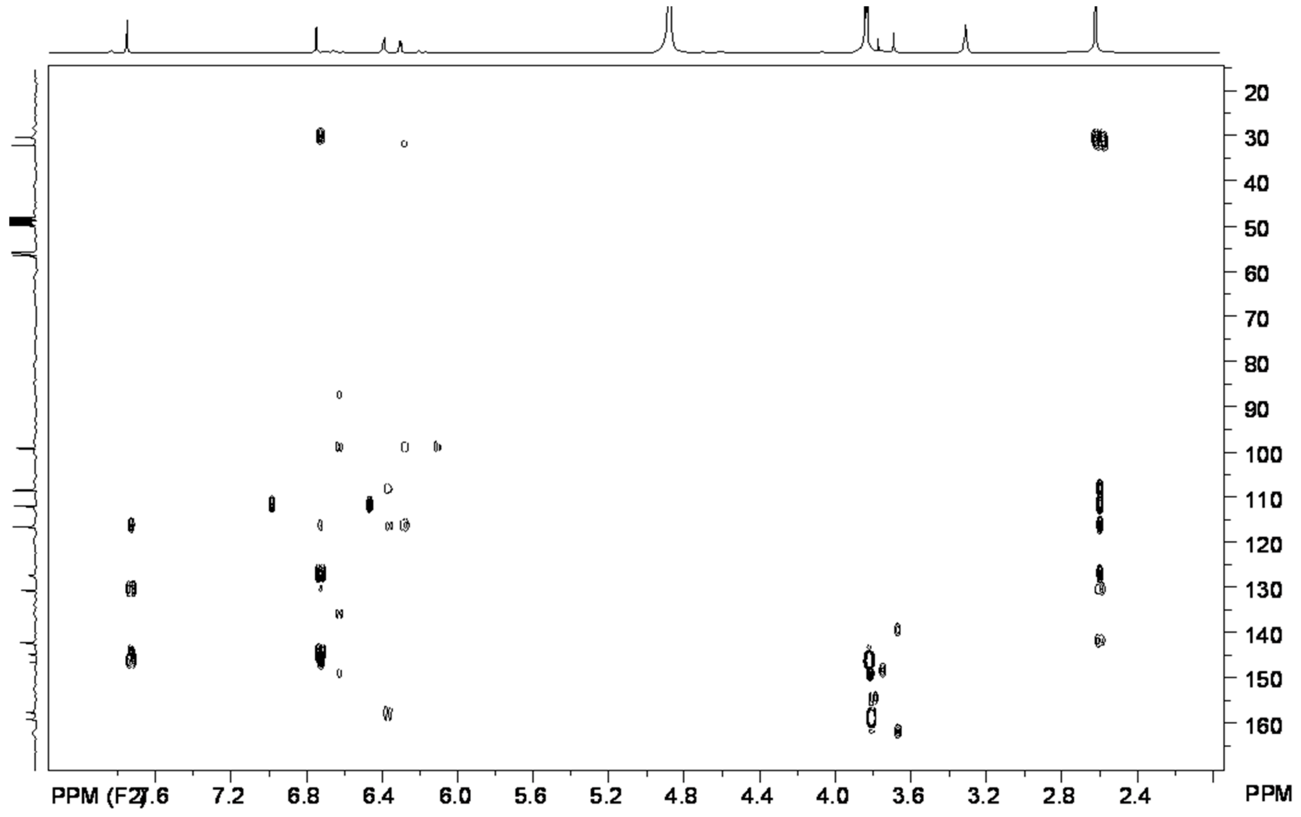

Figure S42. HMBC spectrum (CD_3OD) of compound **6**.

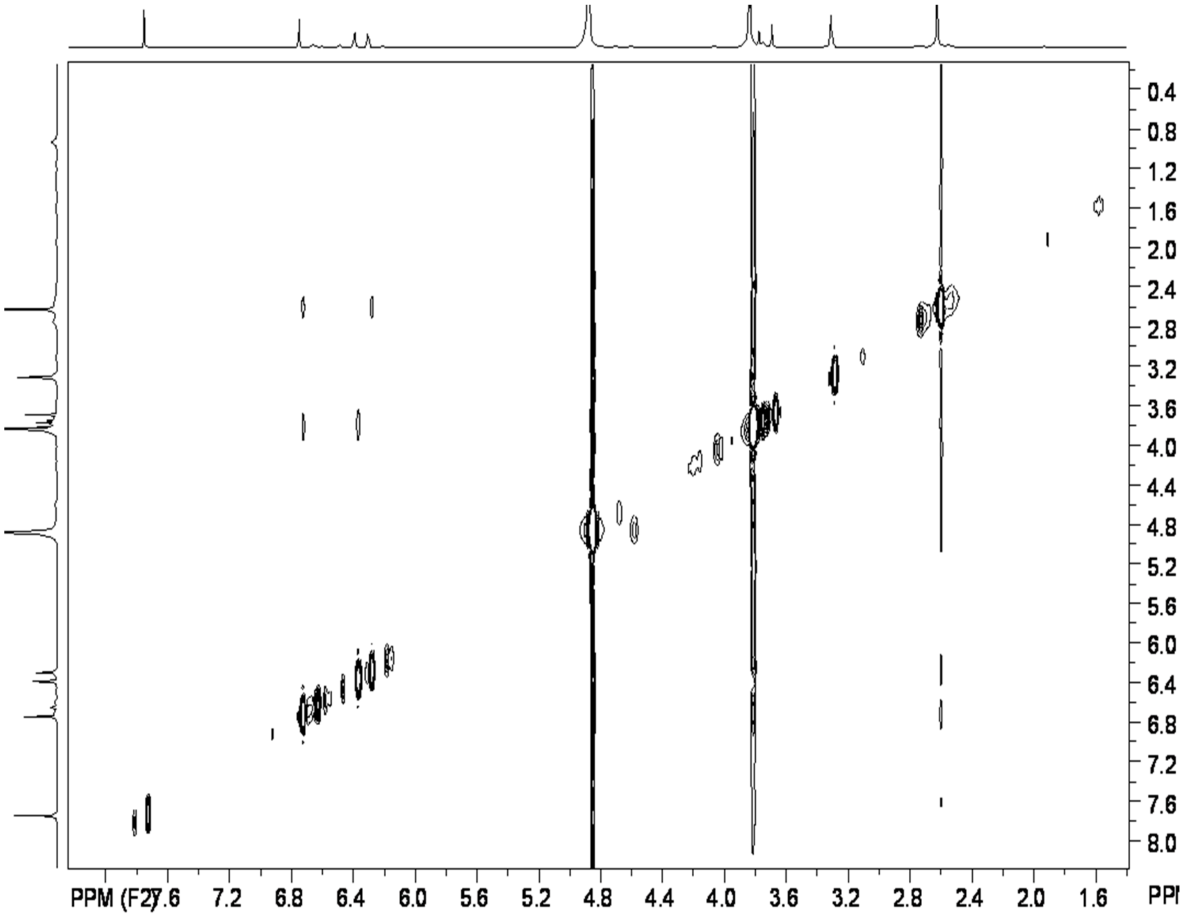

Figure S43. NOESY spectrum (CD_3OD) of compound **6**.

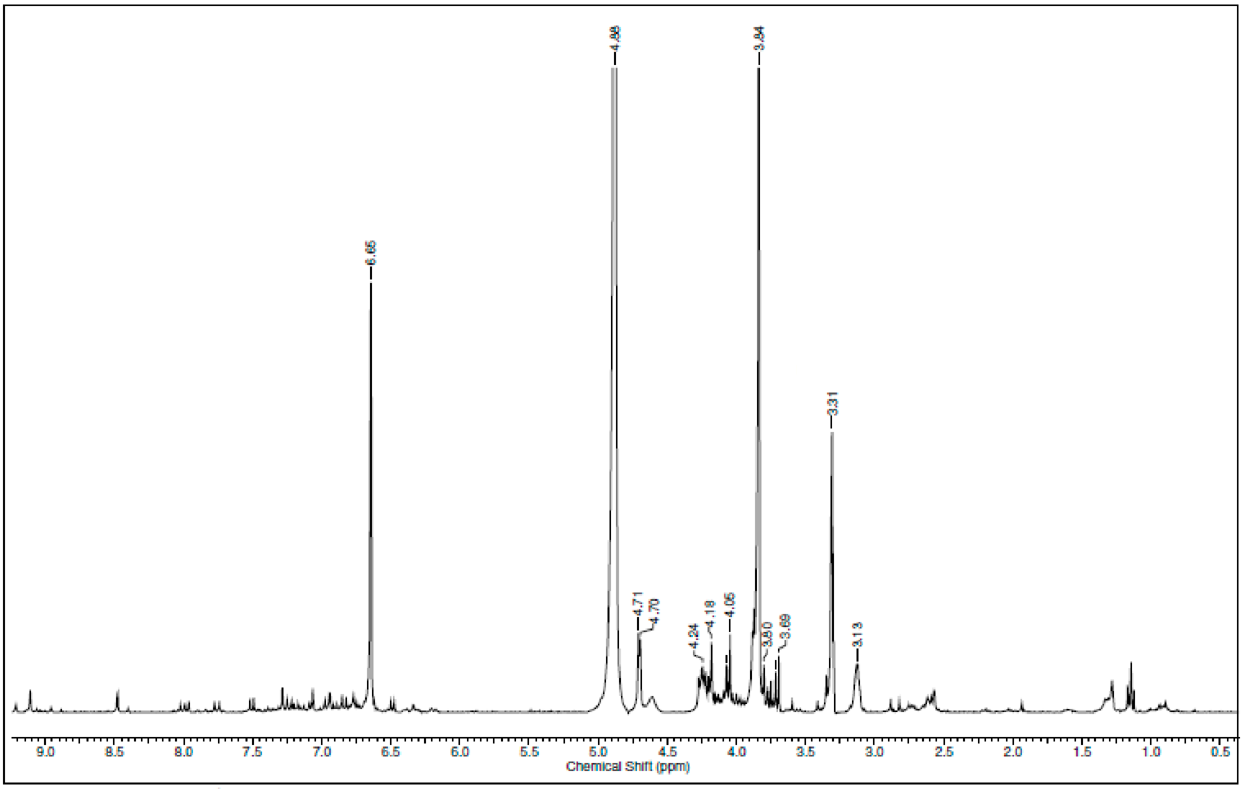

Figure S44. ^{1}H NMR spectrum (300 MHz, CD_3OD) of compound **7**.

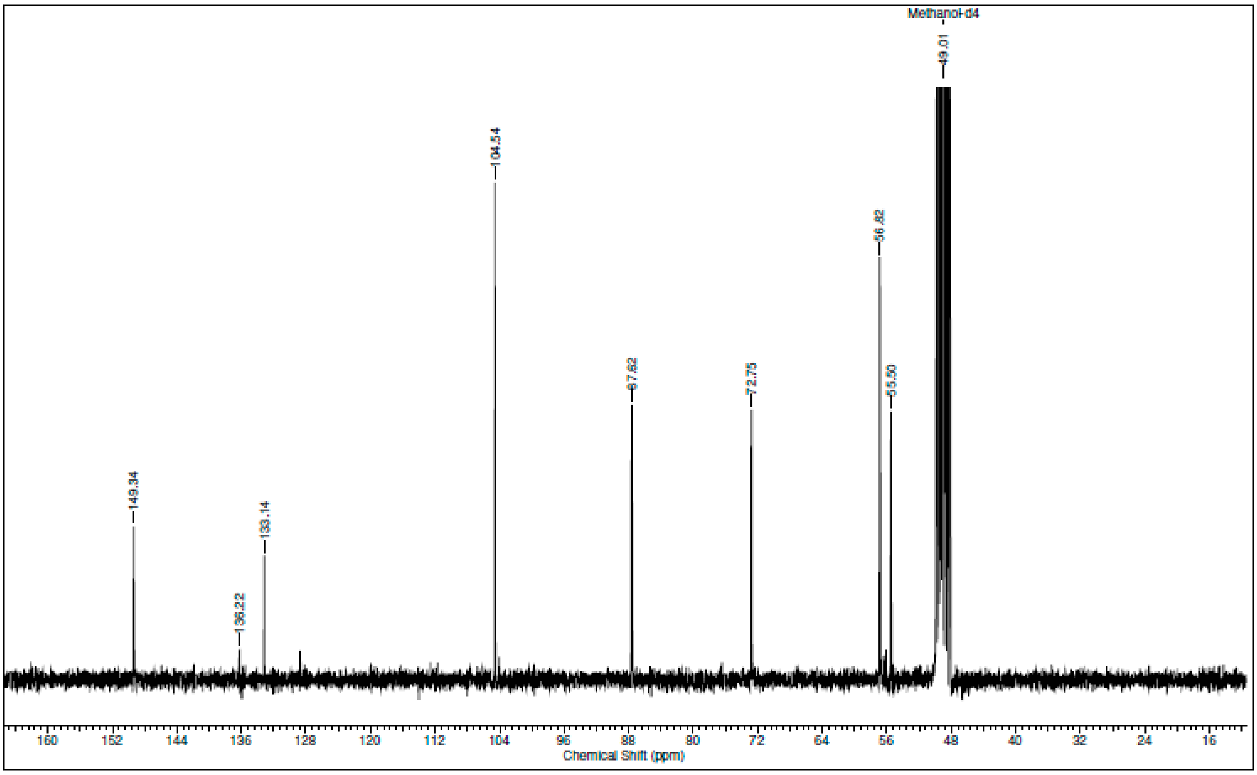

Figure S45. ^{13}C NMR spectrum (75 MHz, CD_3OD) of compound **7**.

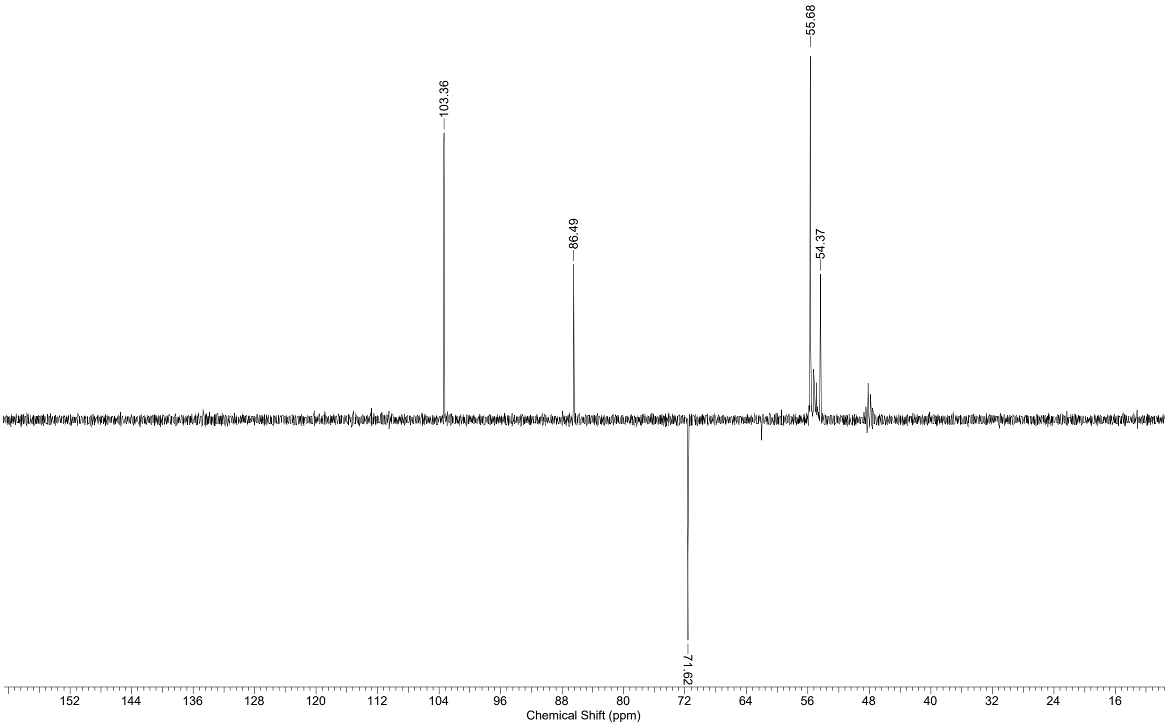

Figure S46. DEPT-135 spectrum (CD_3OD) of compound **7**.

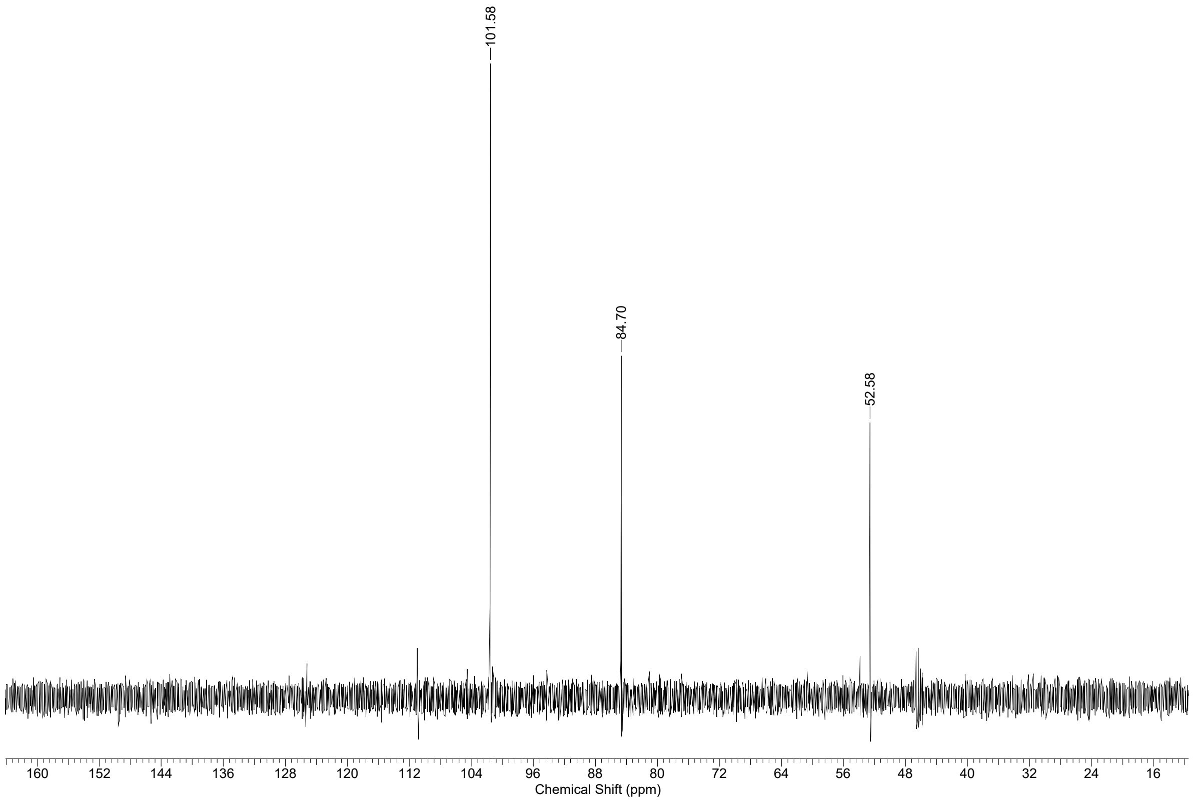

Figure S47. DEPT-90 spectrum (CD_3OD) of compound **7**.

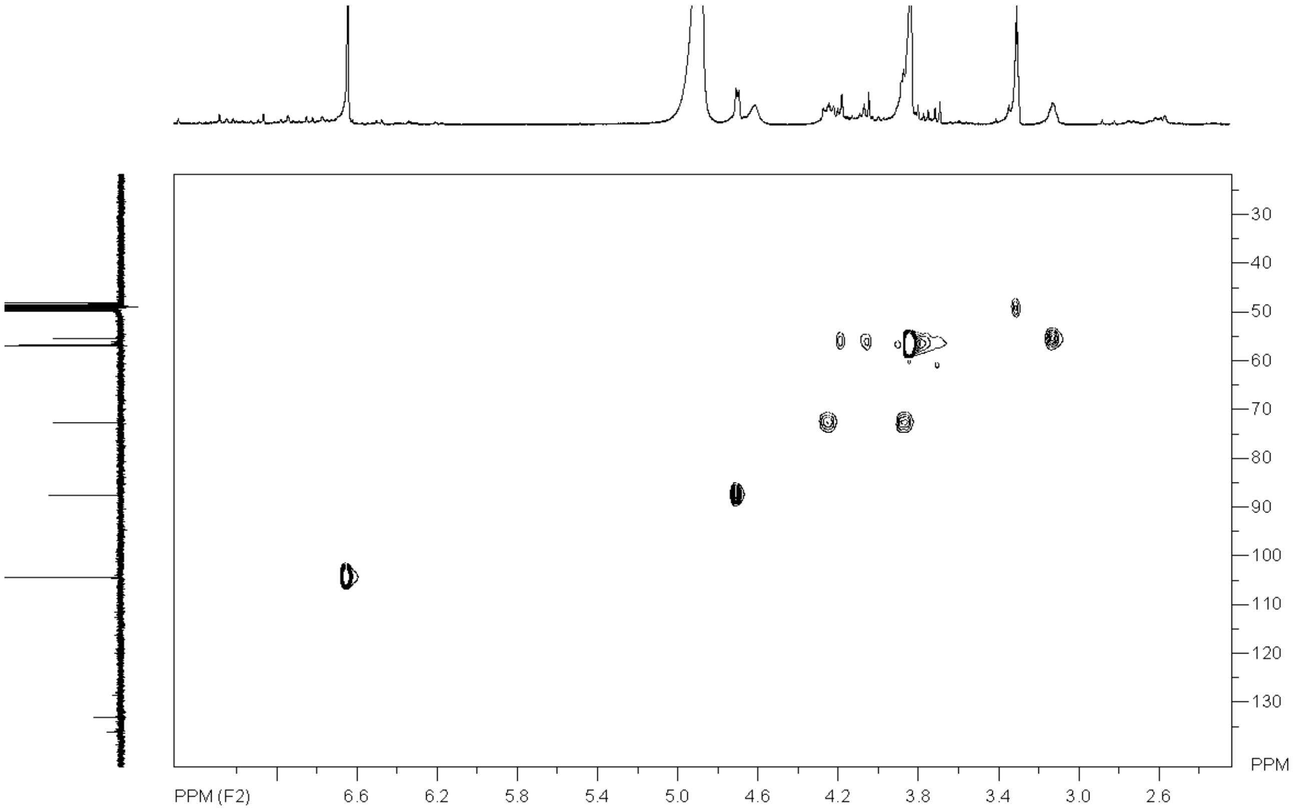

Figure S48. HSQC spectrum (CD_3OD) of compound **7**.

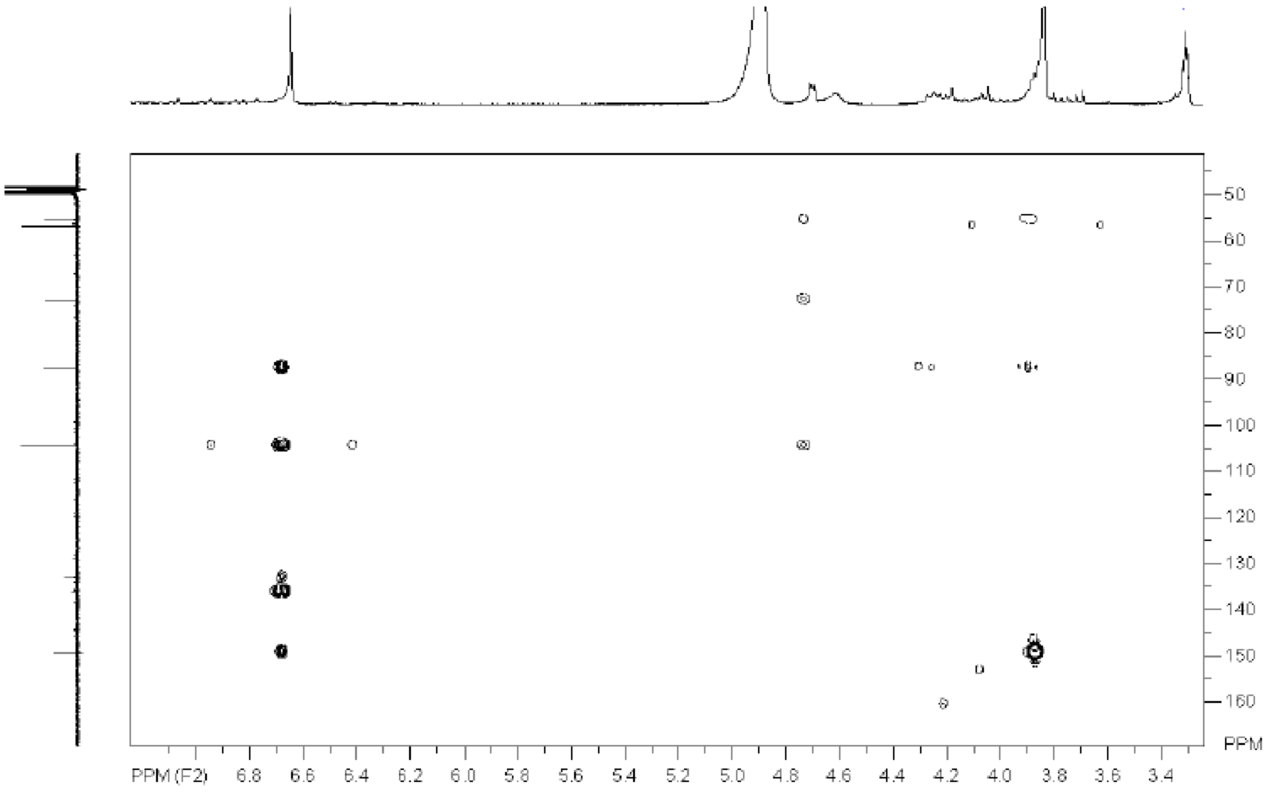

Figure S49. HMBC spectrum (CD3OD) of compound **7**.

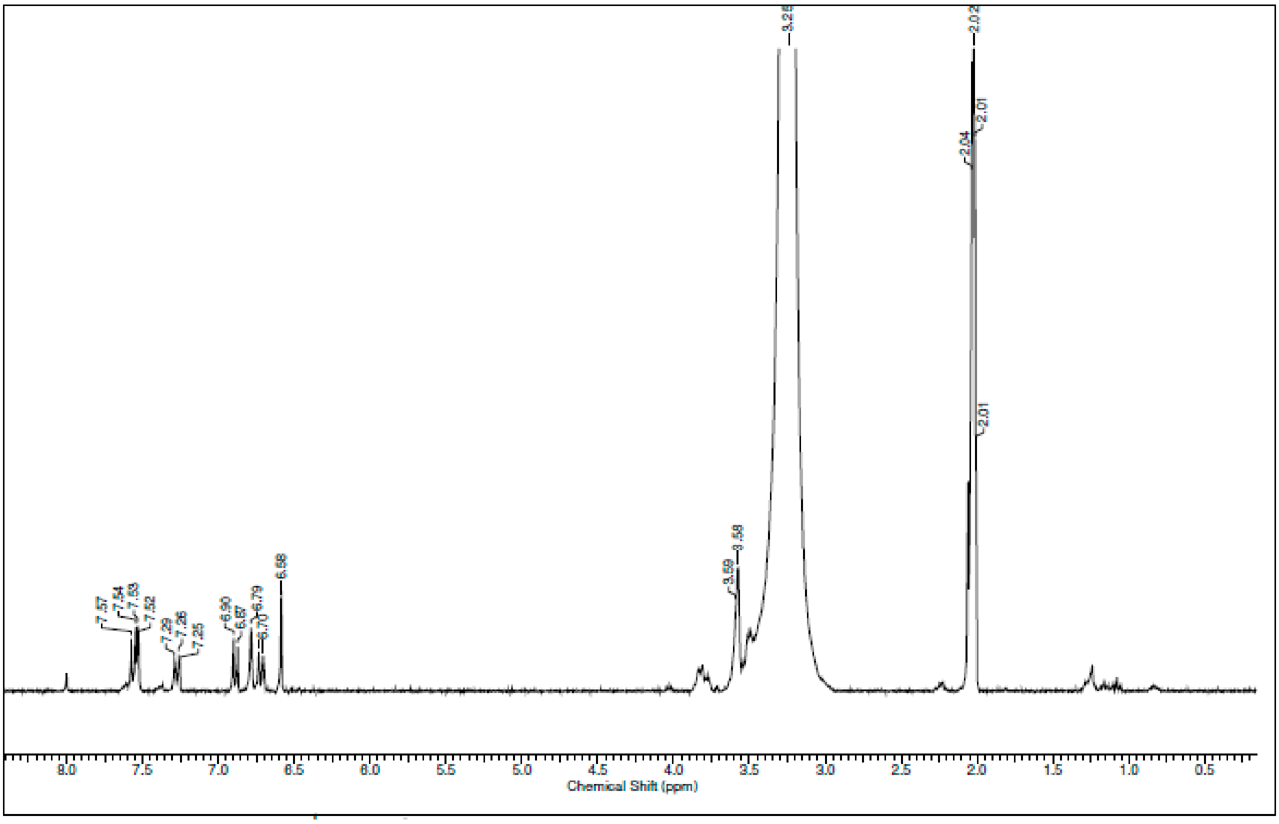

Figure S50. ¹H NMR spectrum (300 MHz, acetone-d6) of compound **8**.

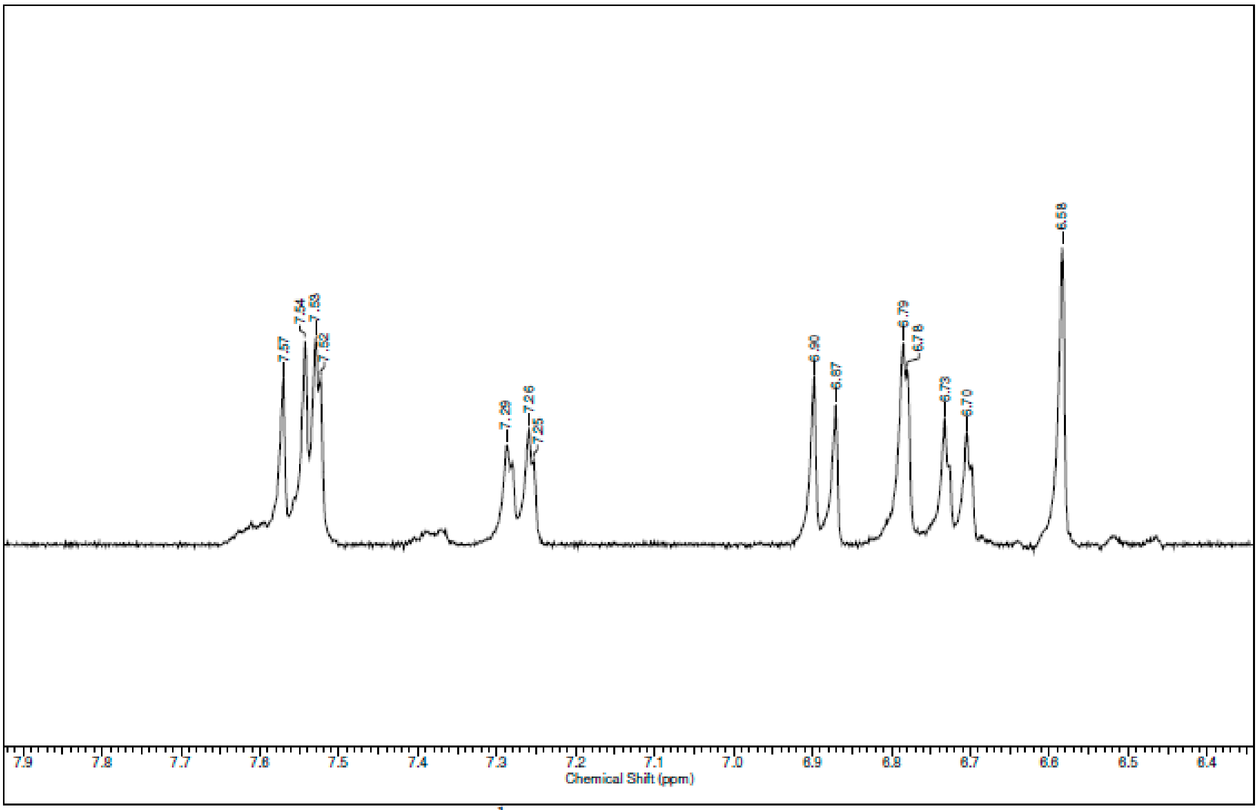

Figure S51. ^{1}H NMR spectrum (expansion) of compound **8**.

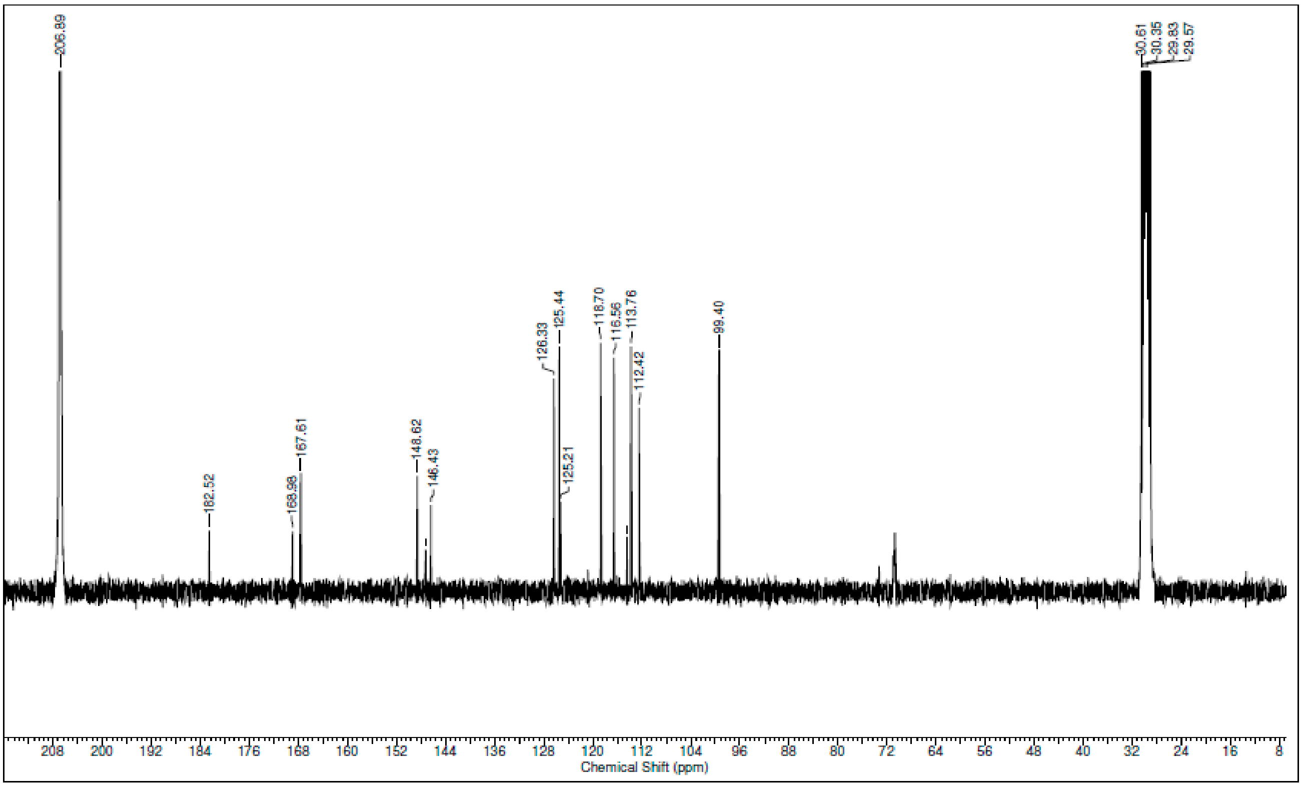

Figure S52. ^{13}C NMR spectrum (75 MHz, acetone-d_6) of compound **8**.

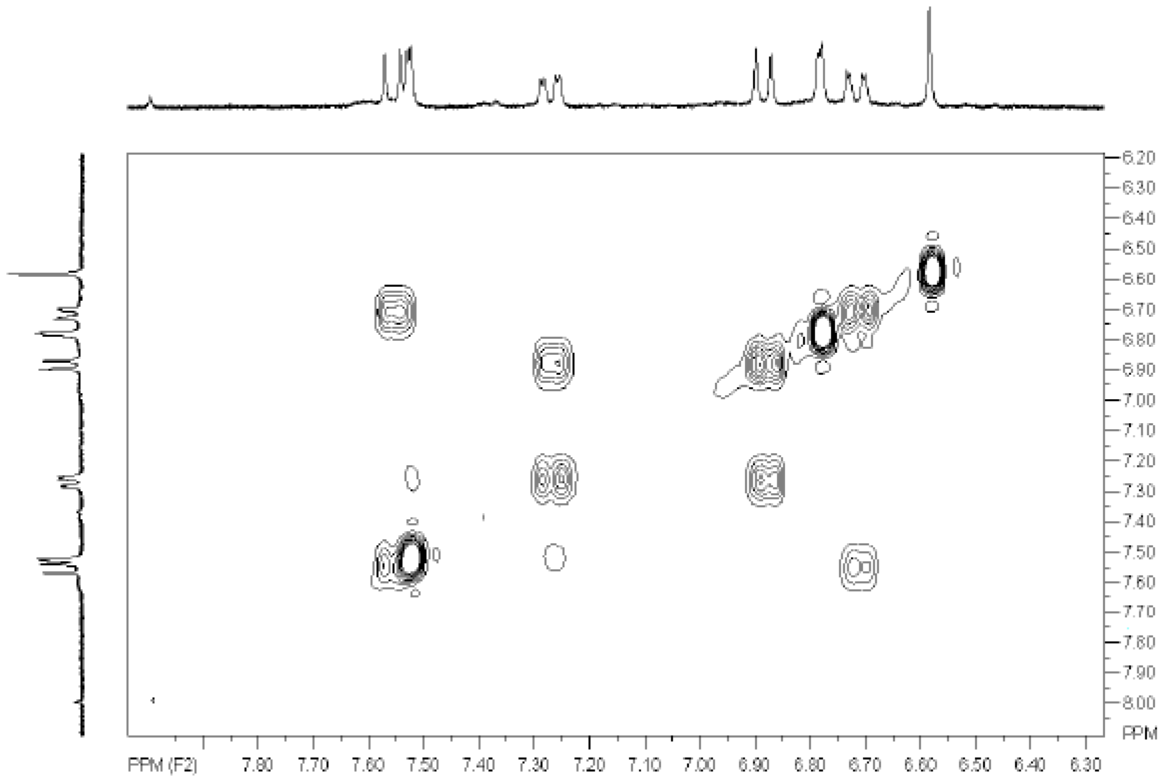

Figure S53. ^{1}H-^{1}H COSY spectrum (300 MHz, acetone-d_6) of compound **8**.

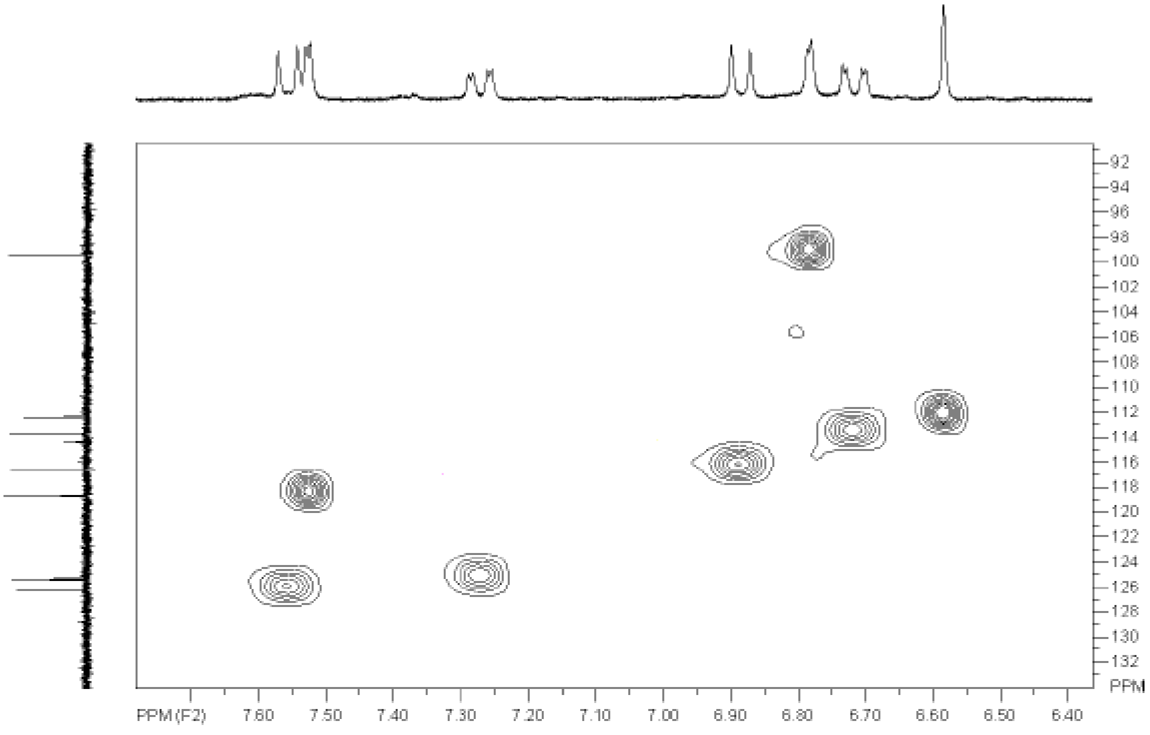

Figure S54. HSQC spectrum (acetone-d_6) of compound **8**.

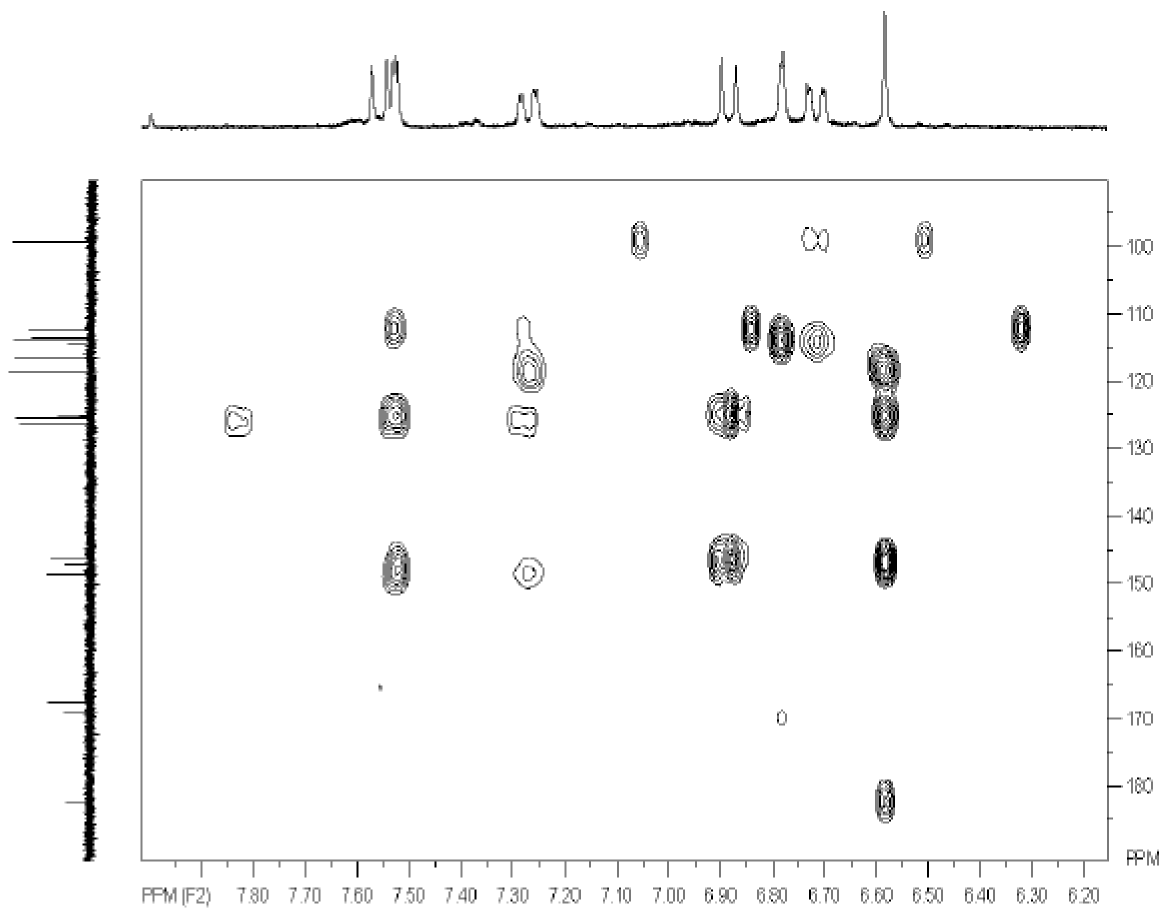

Figure S55. HMBC spectrum (acetone-d_6) of compound **8**.

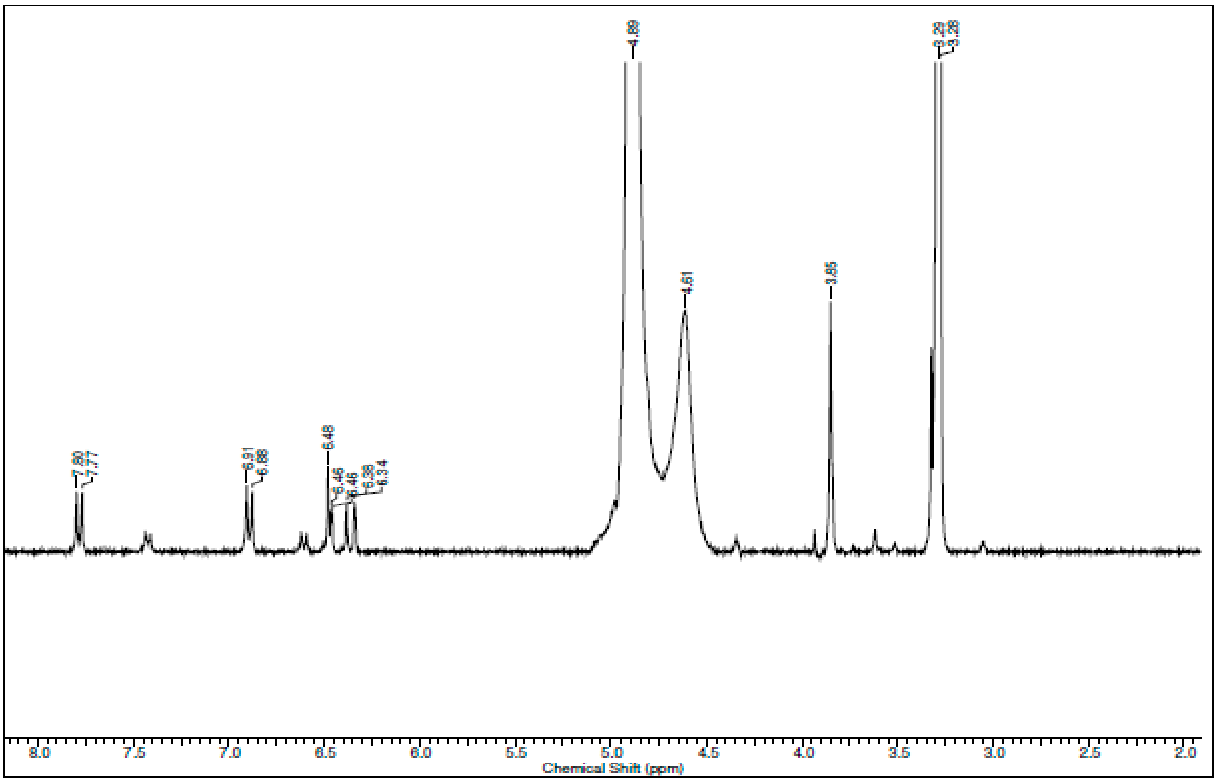

Figure S56. ^{1}H NMR spectrum (300 MHz, CD_3OD) of compound **9**.

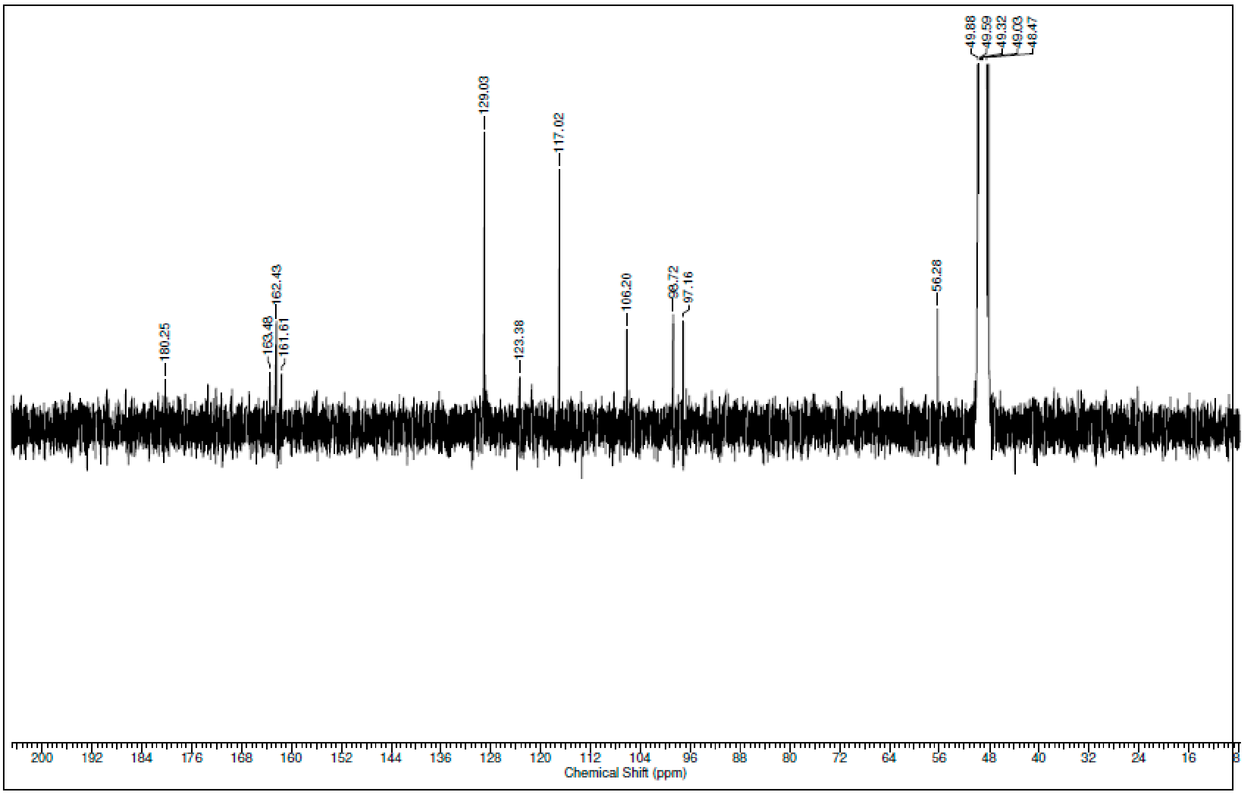

Figure S57. ^{13}C NMR spectrum (75 MHz, CD_3OD) of compound **9**.

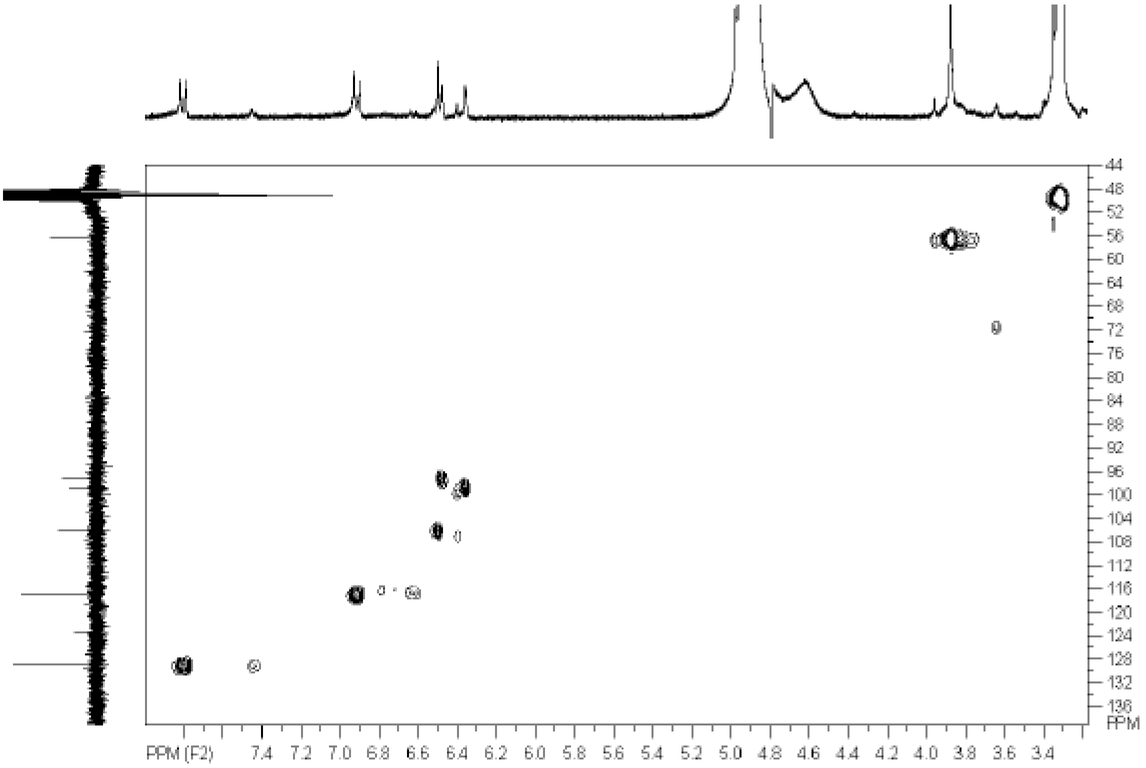

Figure S58. HSQC spectrum (CD_3OD) of compound **9**.

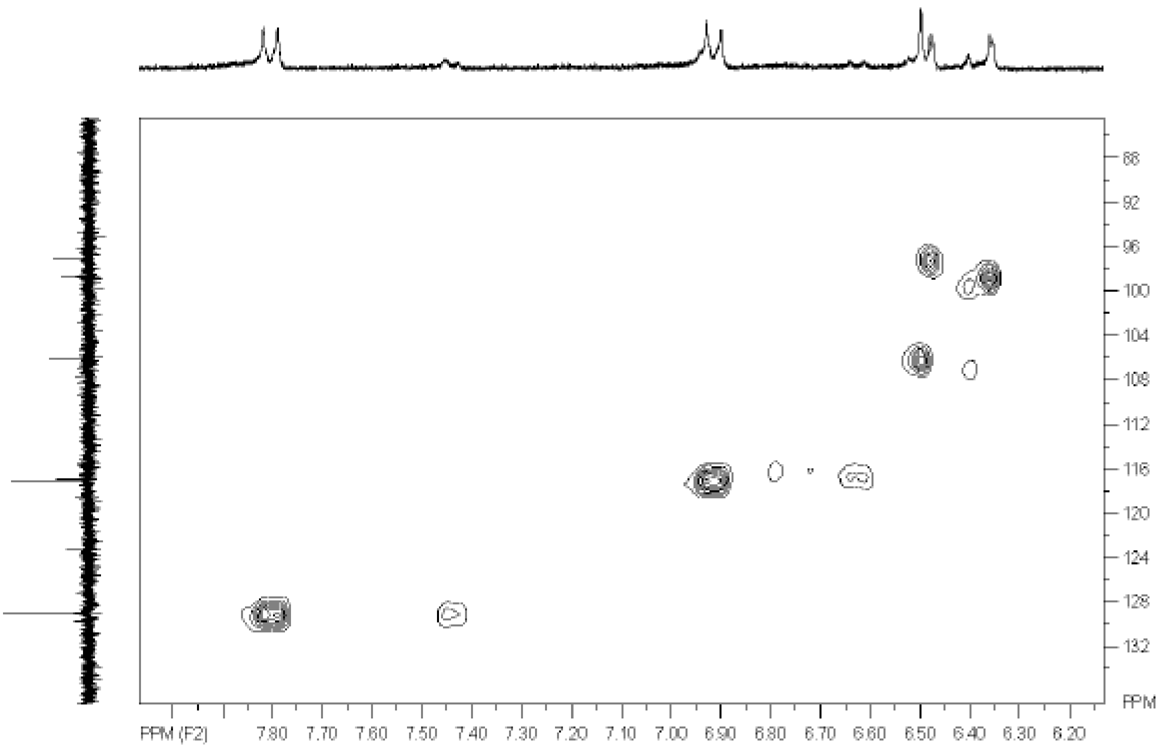

Figure S59. HSQC spectrum (expansion) of compound **9**.

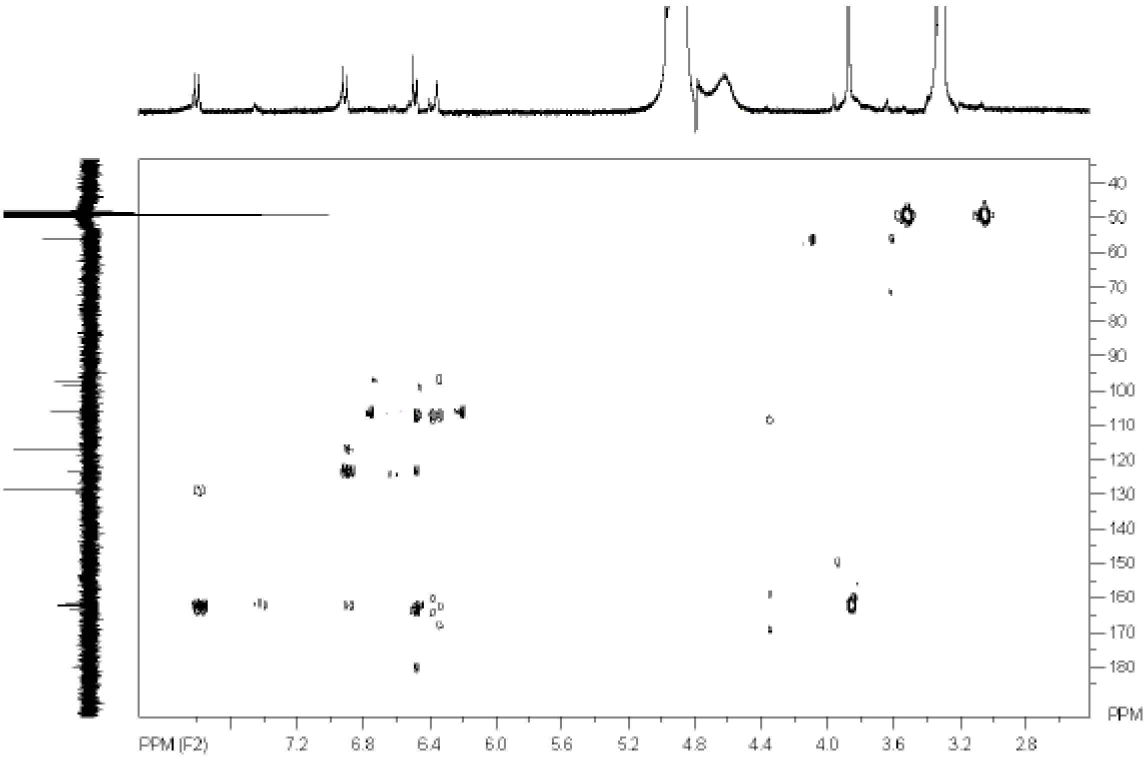

Figure S60. HMBC spectrum (CD_3OD) of compound **9**.

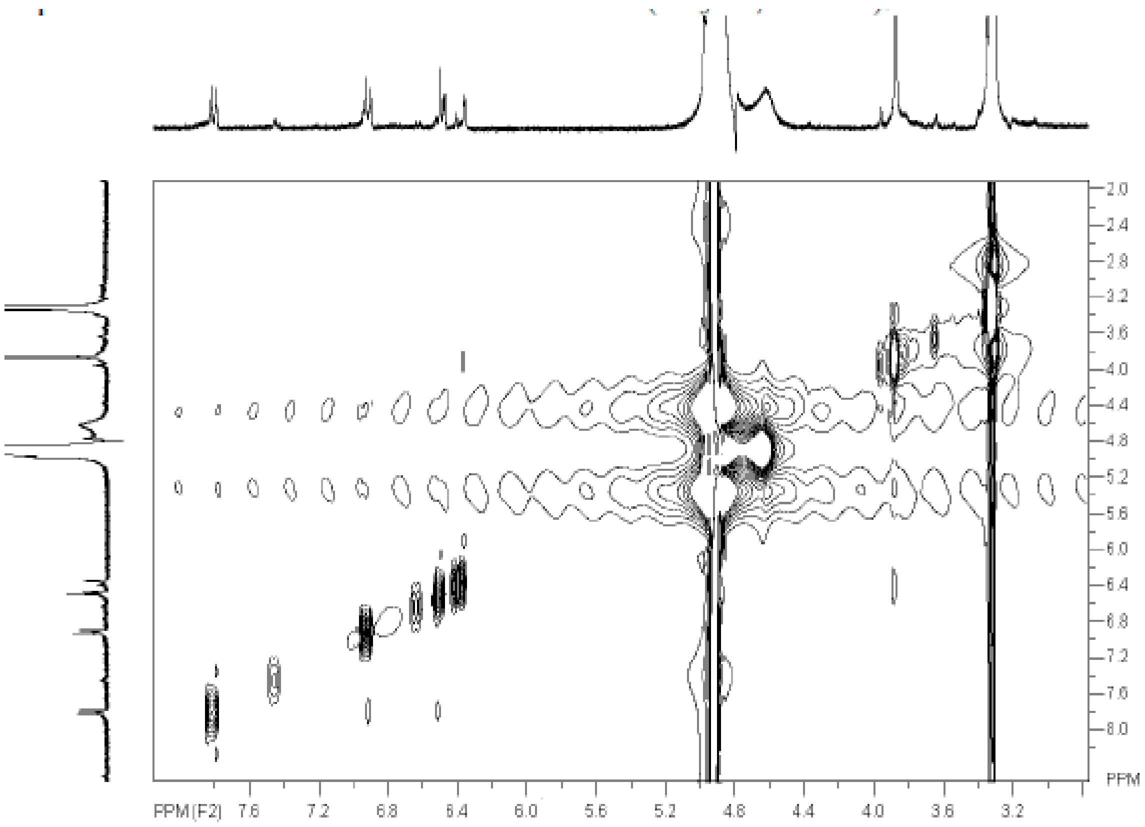

Figure S61. NOESY spectrum (300 MHz, CD_3OD) of compound **9**.

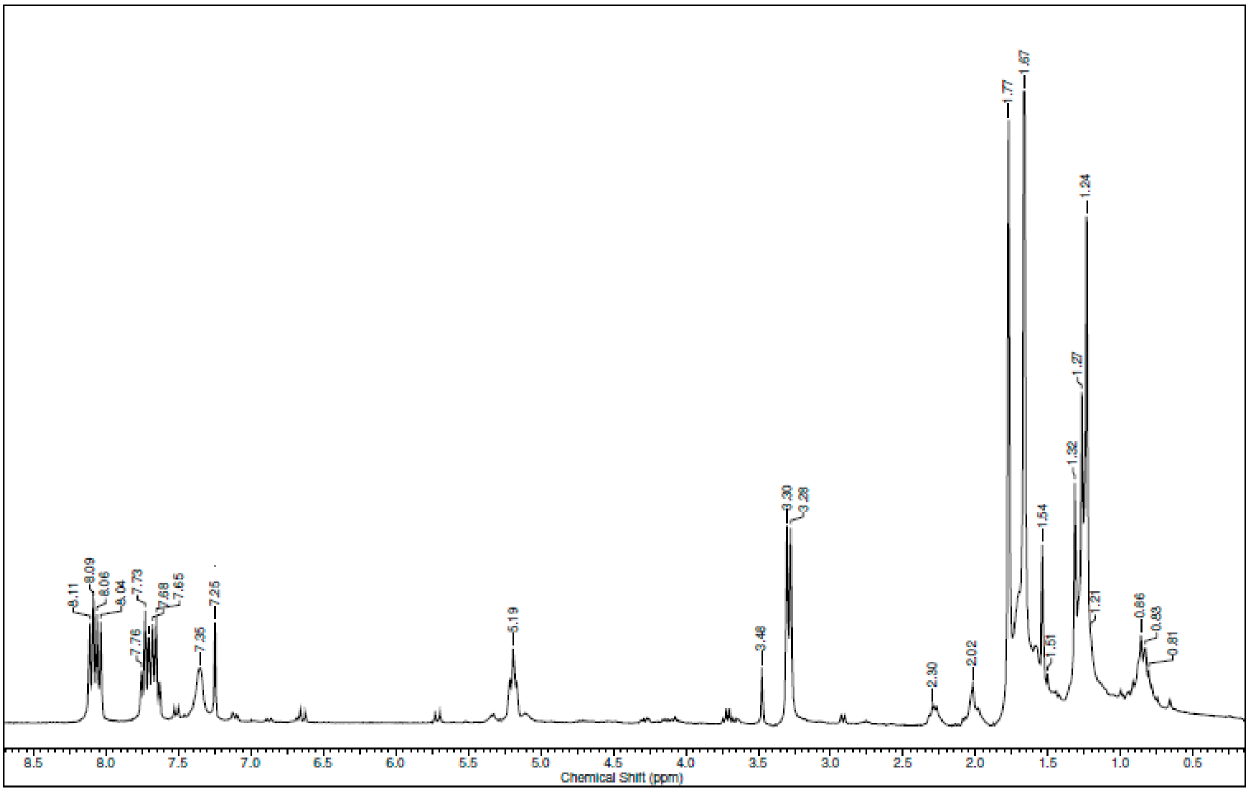

Figure S62. ^{1}H NMR spectrum (300 MHz, $CDCl_3$) of compound **10**.

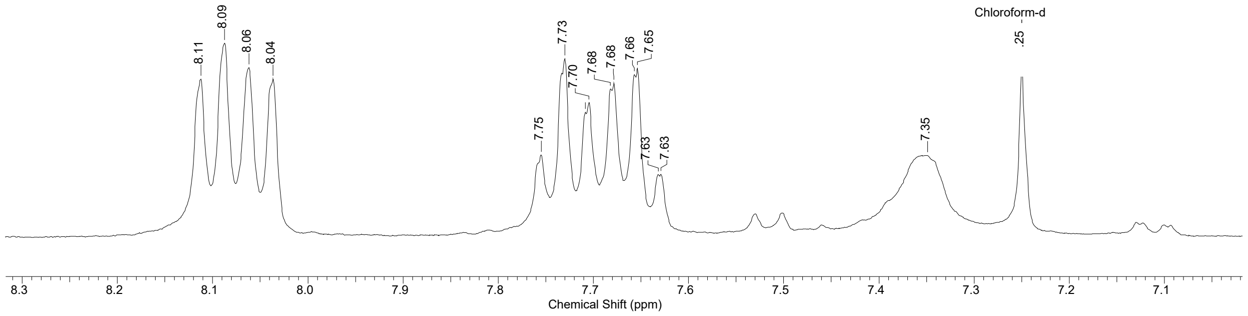

Figure S63. ^{1}H NMR spectrum (expansion) of compound **10**.

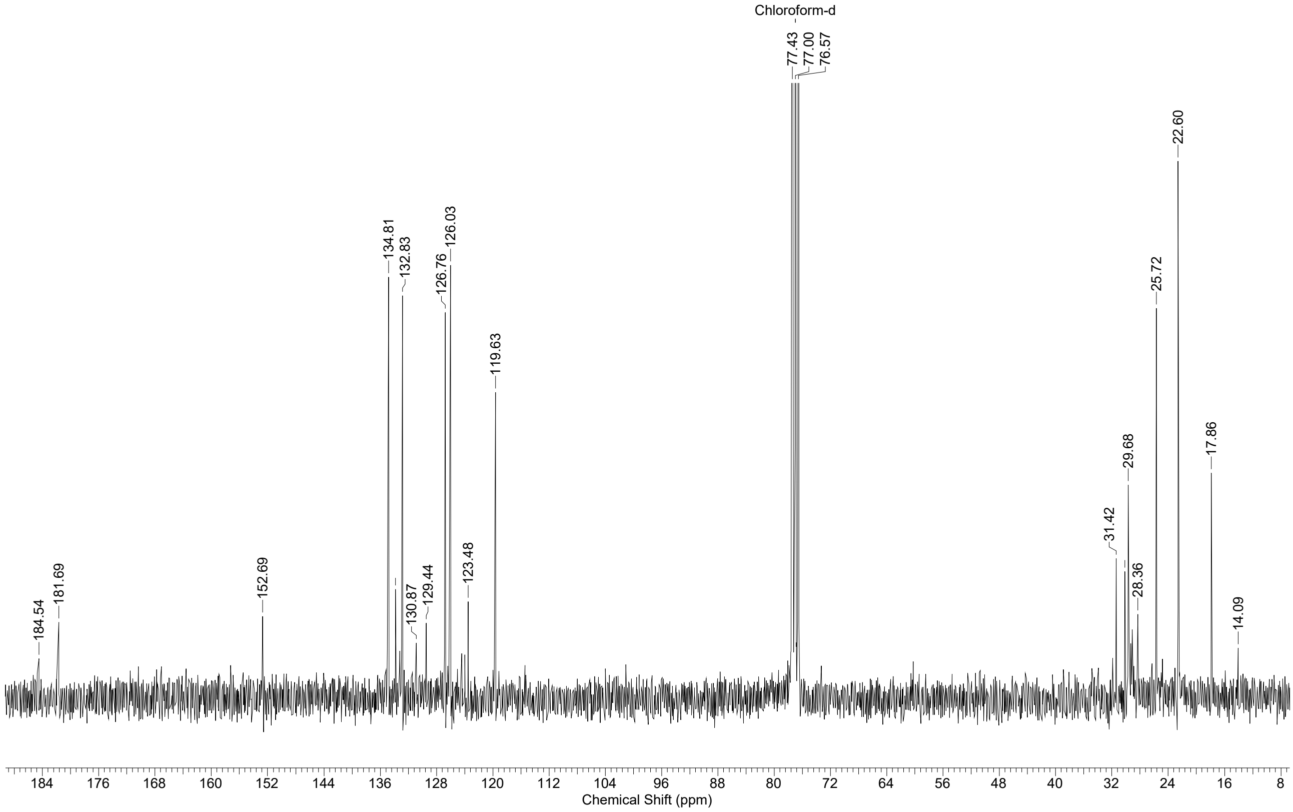

Figure S64. ^{13}C NMR spectrum (75 MHz, $CDCl_3$) of compound **10**.

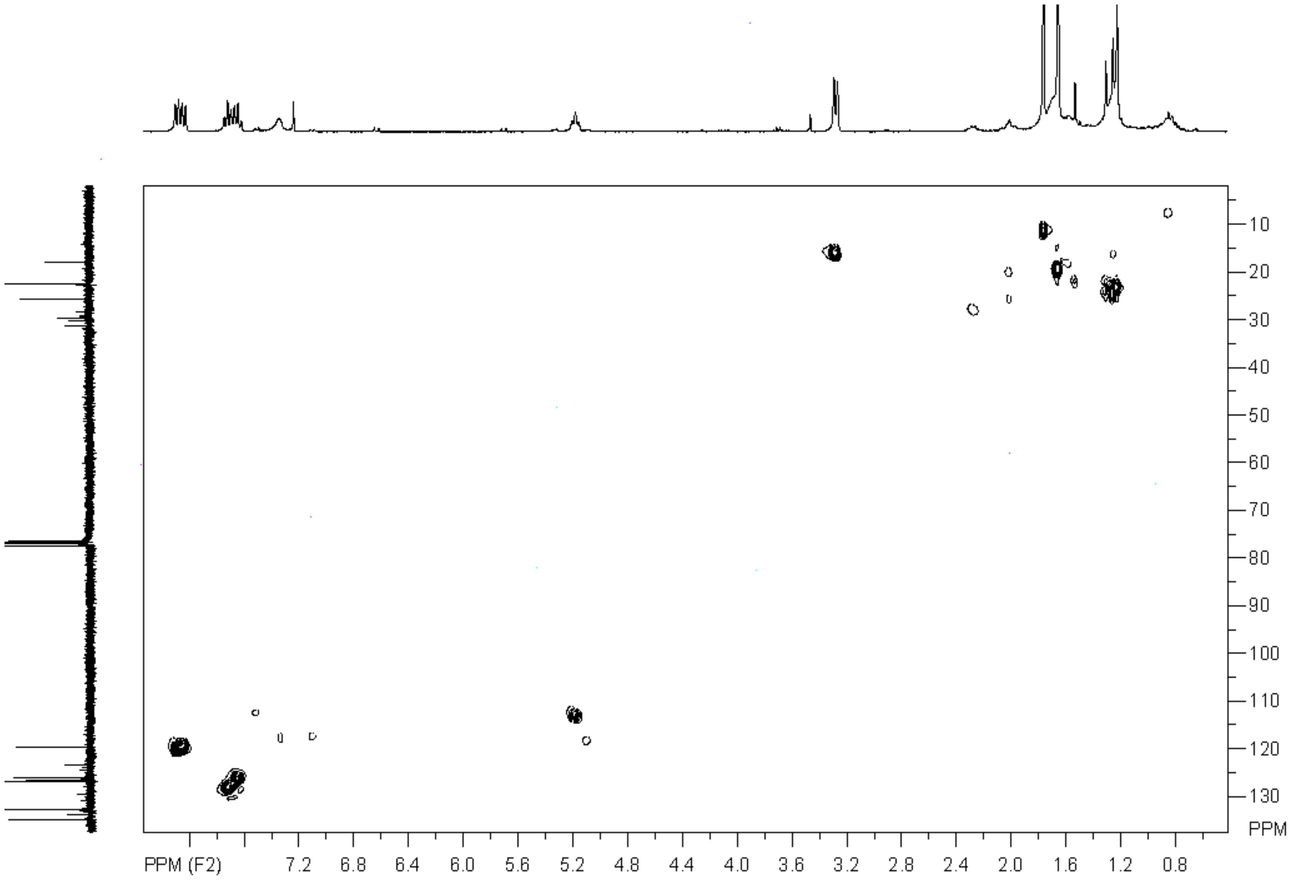

Figure S65. HSQC spectrum ($CDCl_3$) of compound **10**.

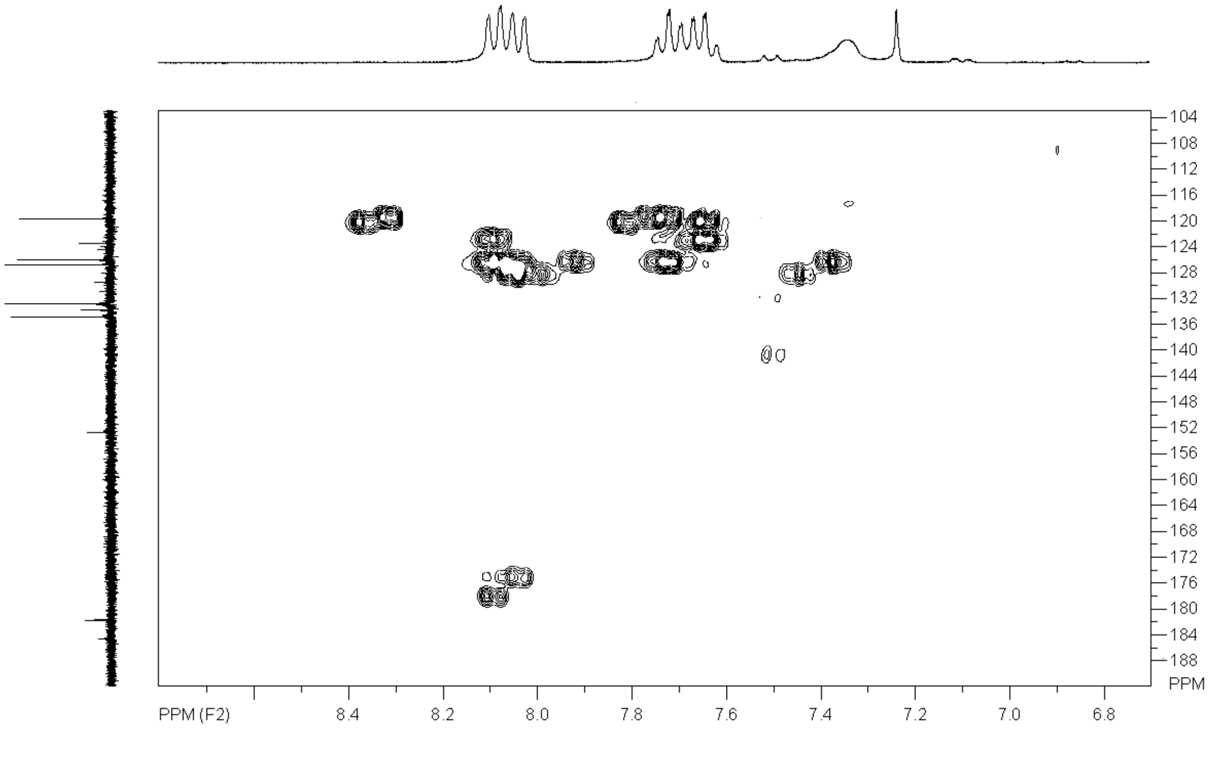

Figure S66. HMBC spectrum (expansion, $CDCl_3$) of compound **10**.

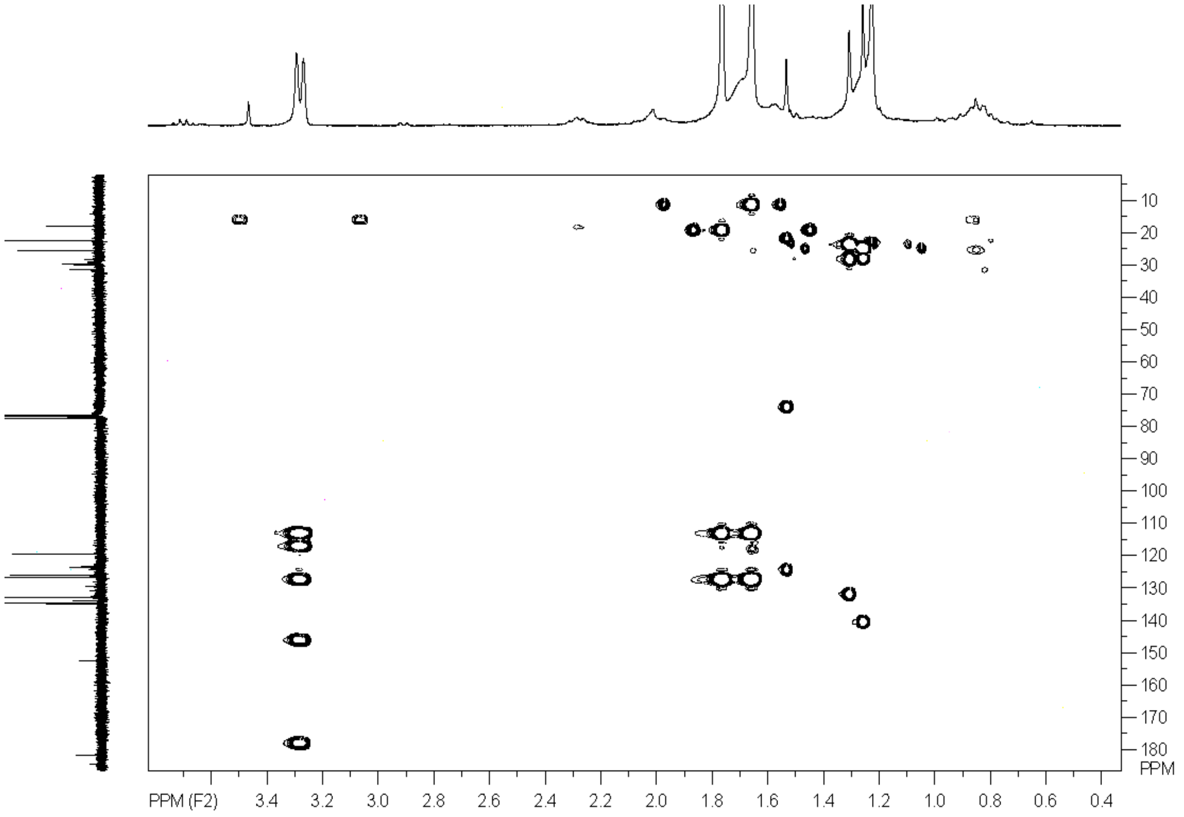

Figure S67. HMBC spectrum (expansion, $CDCl_3$) of compound **10**.

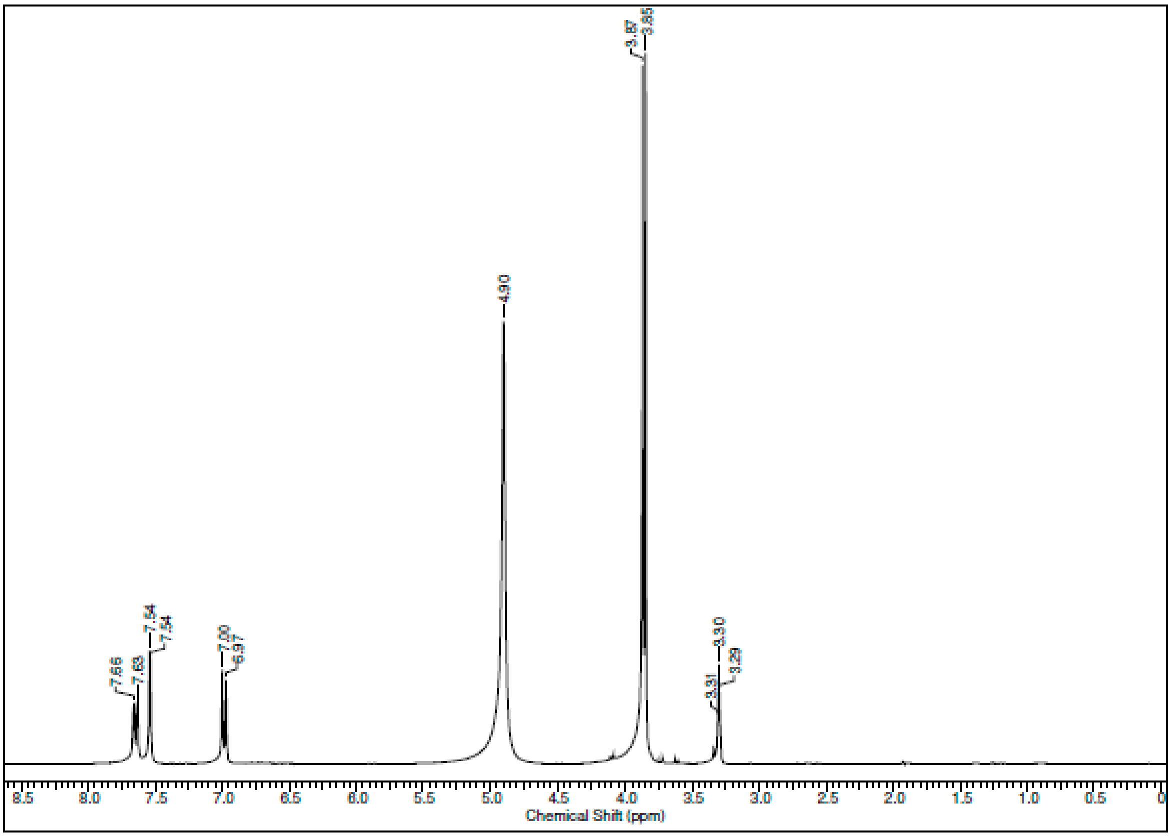

Figure S68. ^1H NMR spectrum (300 MHz, CD_3OD) of compound **11**.

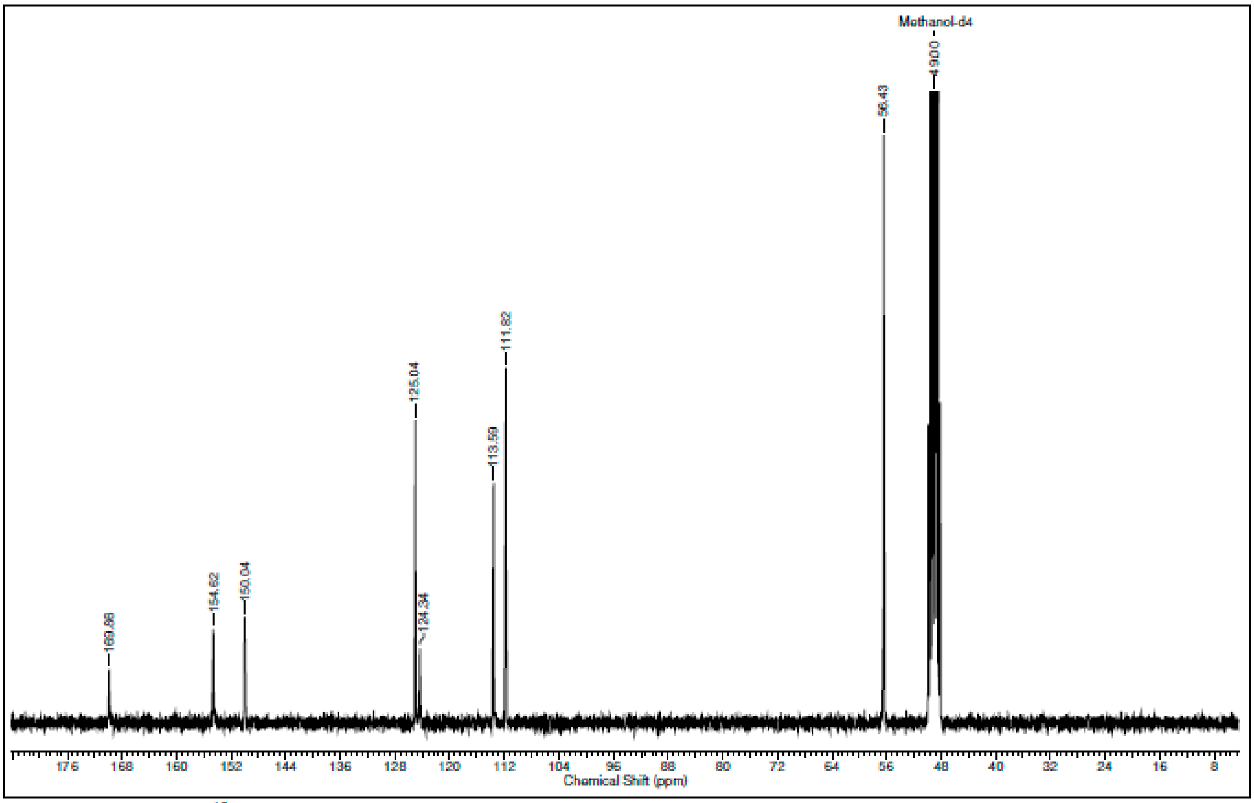

Figure S69. ^{13}C NMR spectrum (75 MHz, CD_3OD) of compound **11**.

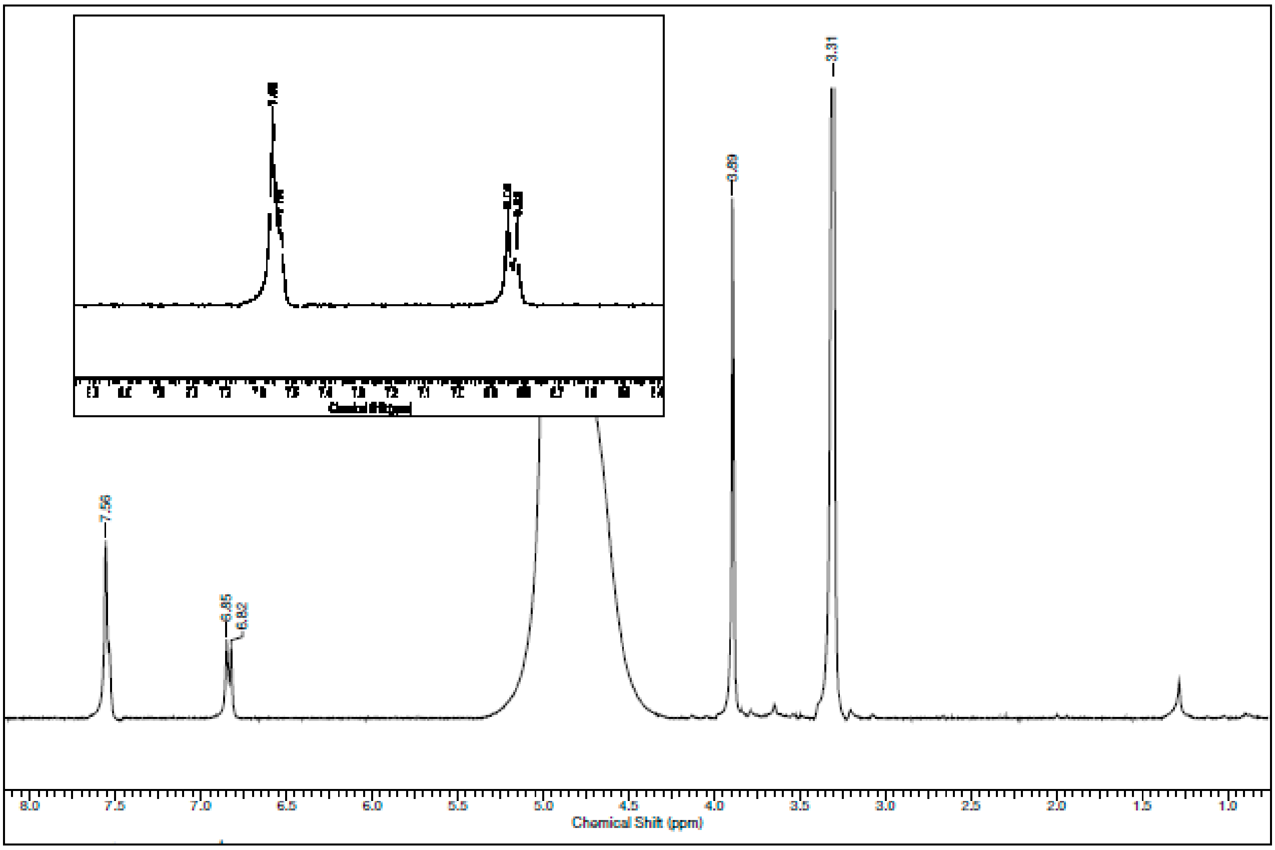

Figure S70. ^{1}H NMR spectrum (300 MHz, CD_3OD) of compound **12**.

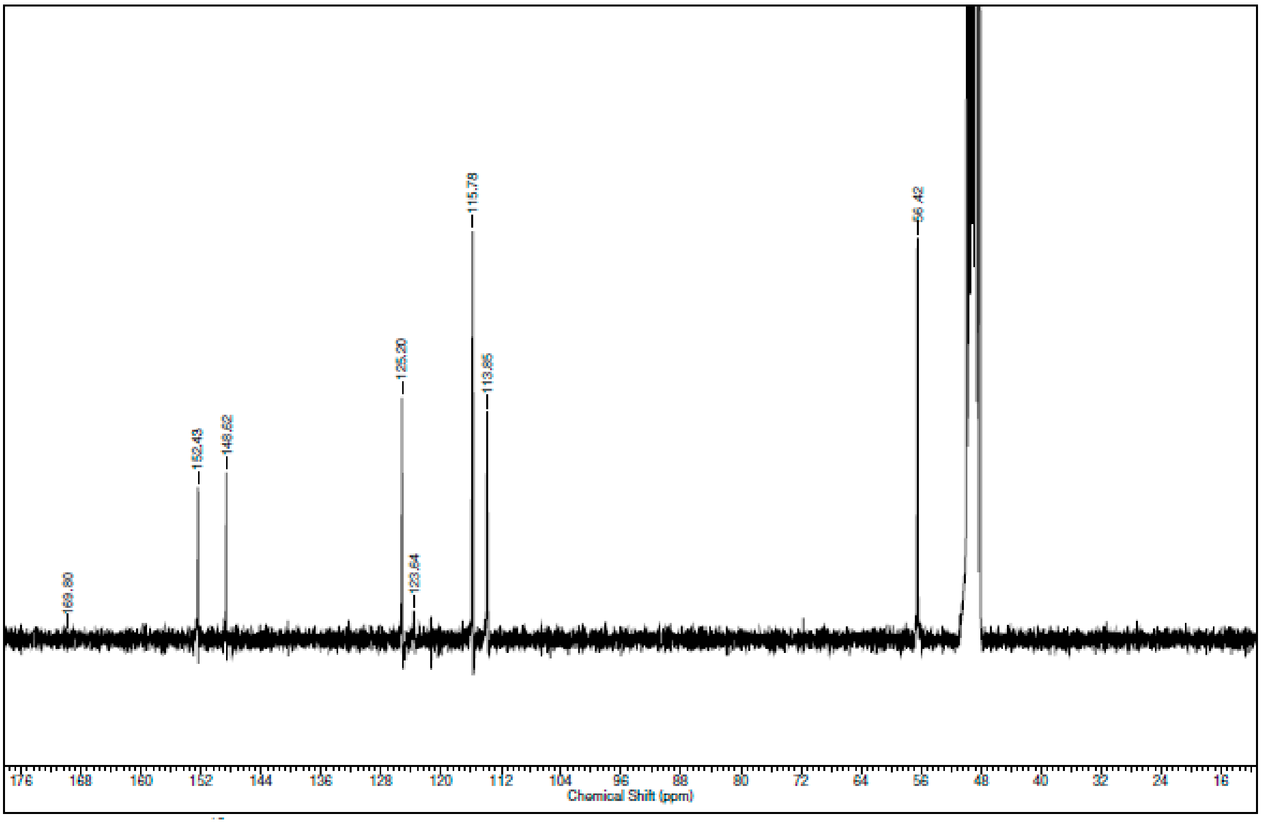

Figure S71. ^{13}C NMR spectrum (75 MHz, CD_3OD) of compound **12**.

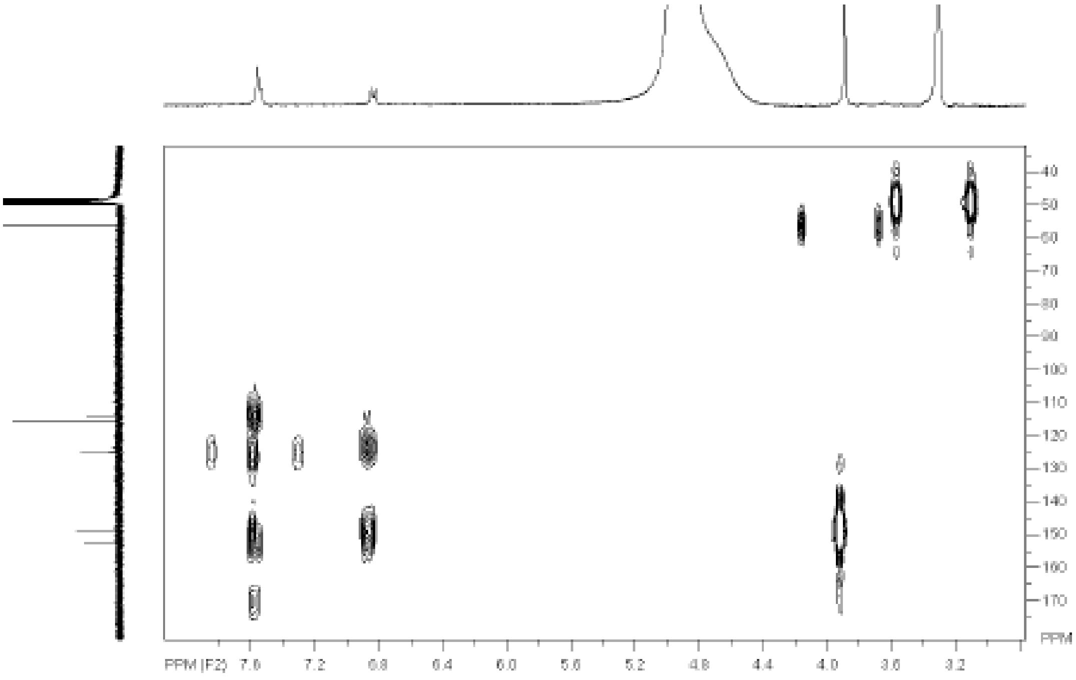

Figure S72. HSQC spectrum (CD_3OD) of compound **12**.

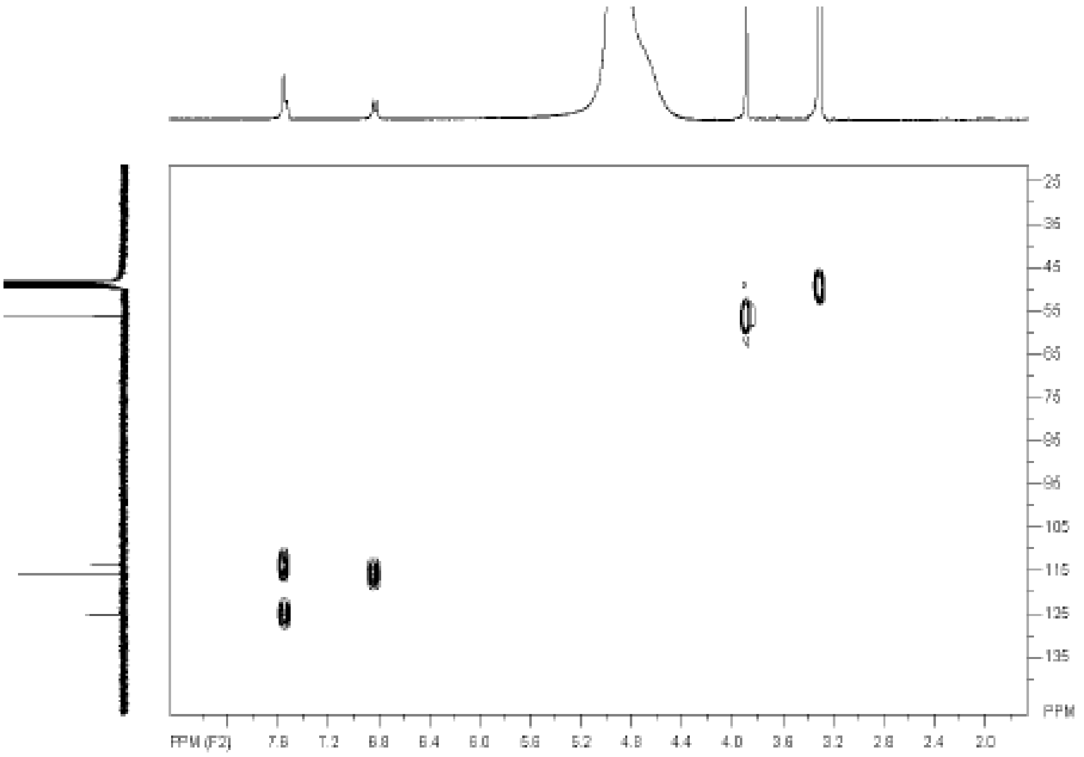

Figure S73. HMBC spectrum (CD_3OD) of compound **12**.

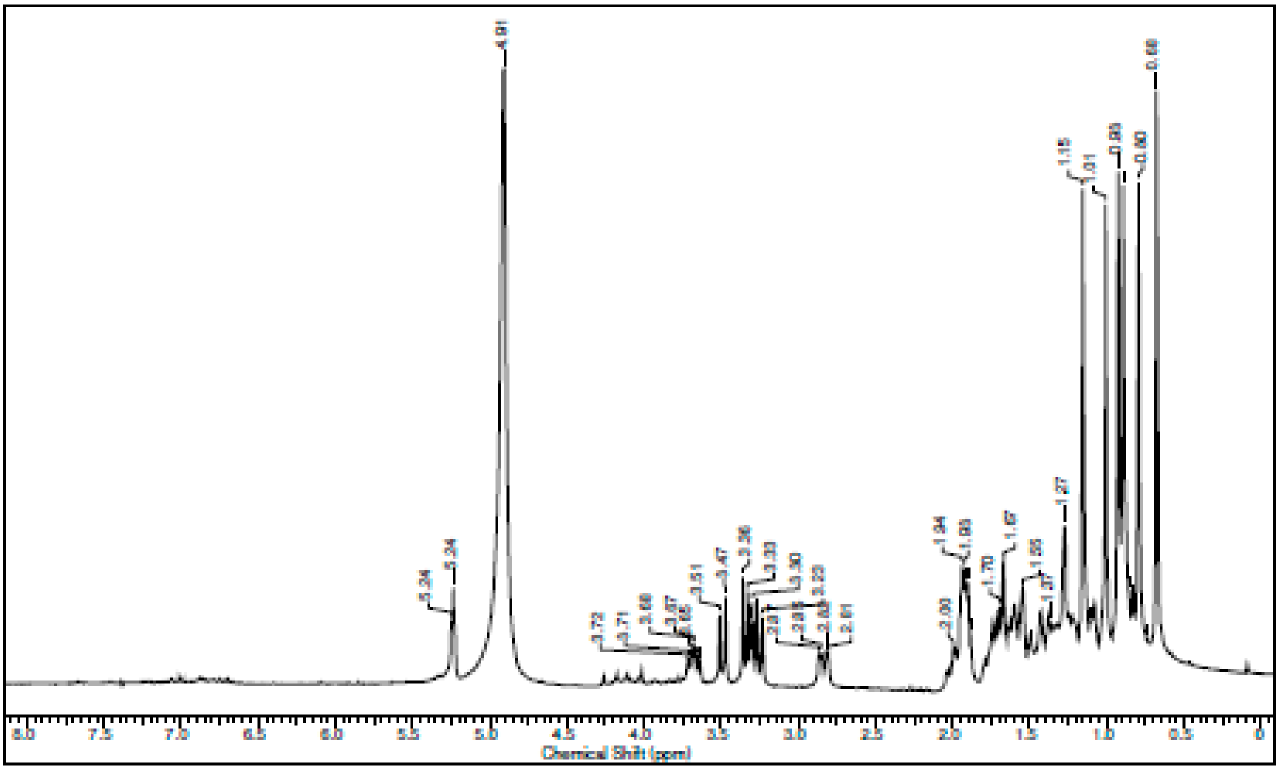

Figure S74. ^1H NMR spectrum (300 MHz, CD_3OD) of compound **13**.

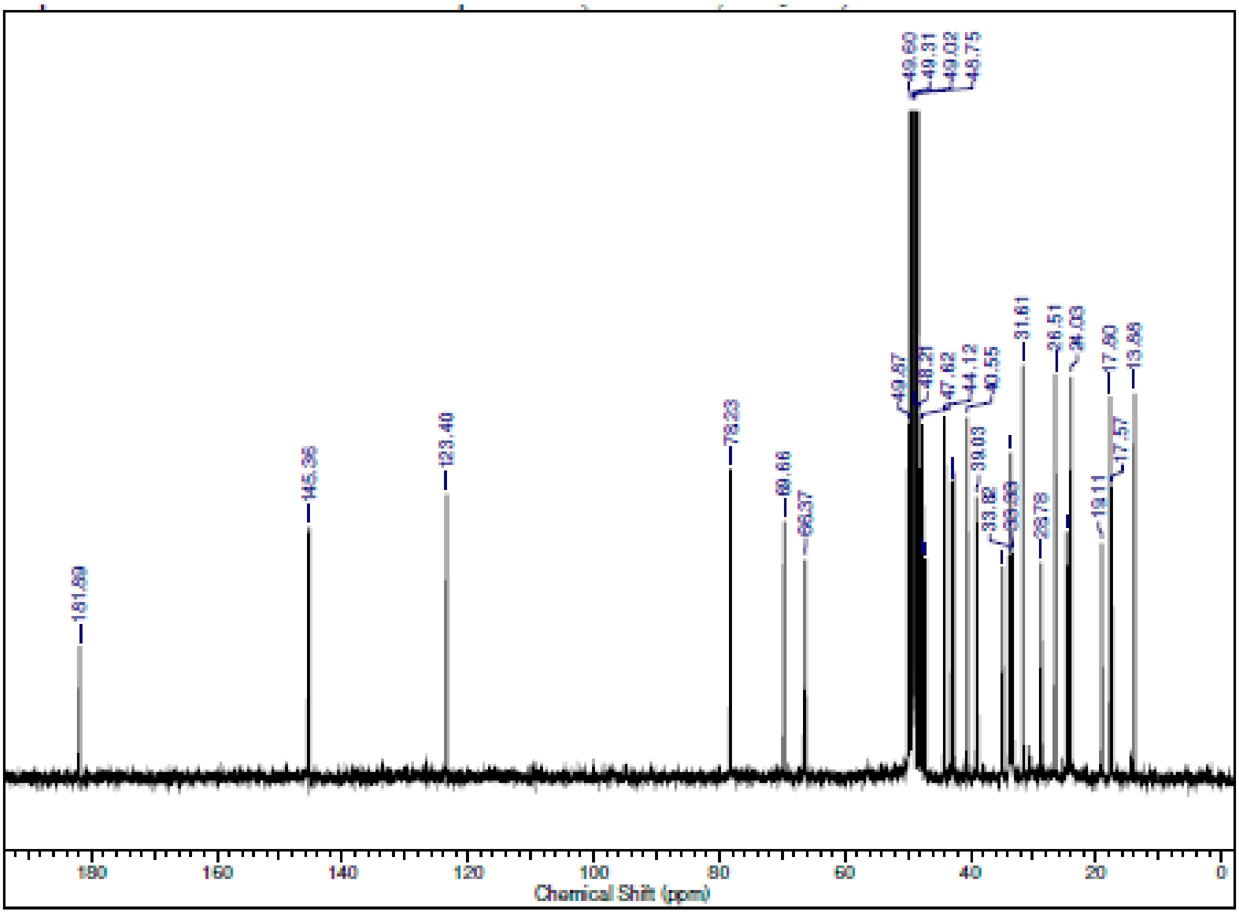

Figure S75. ^{13}C NMR spectrum (75 MHz, CD_3OD) of compound **13**.

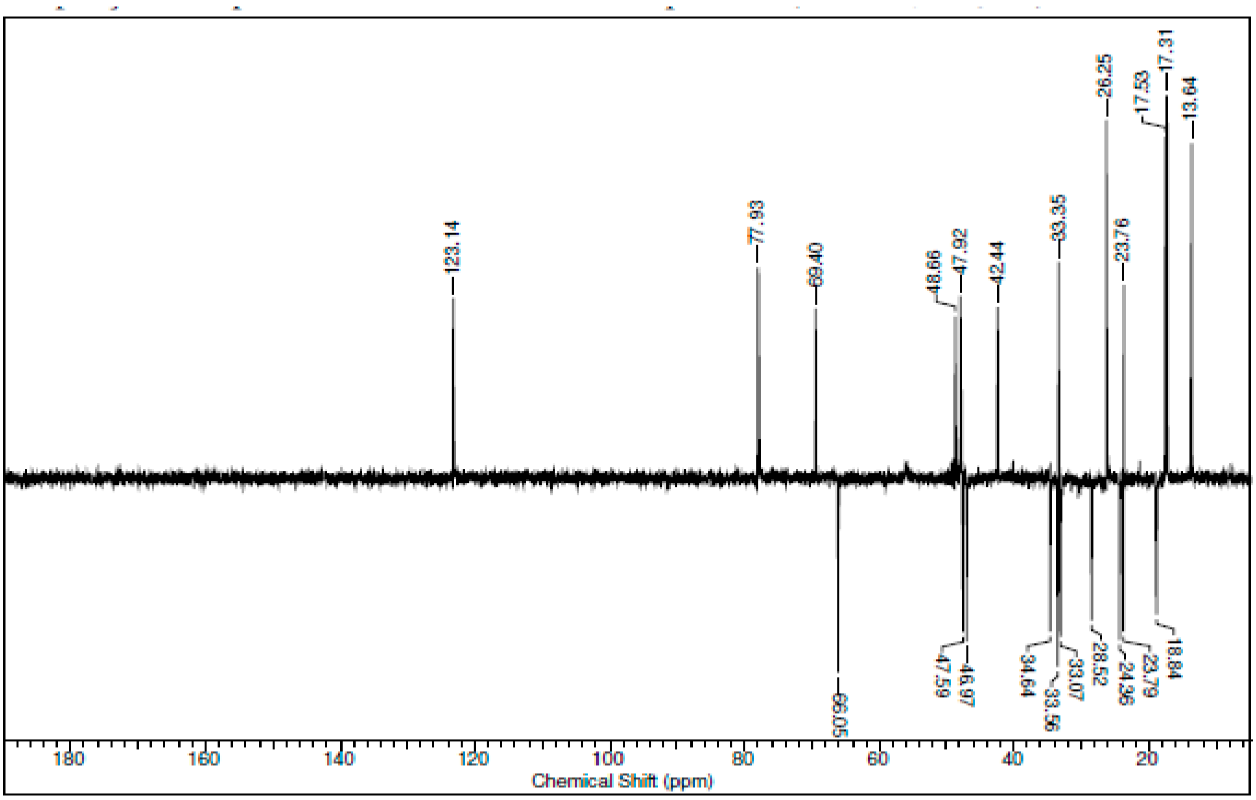

Figure S76. DEPT-135 spectrum (CD_3OD) of compound **13**.

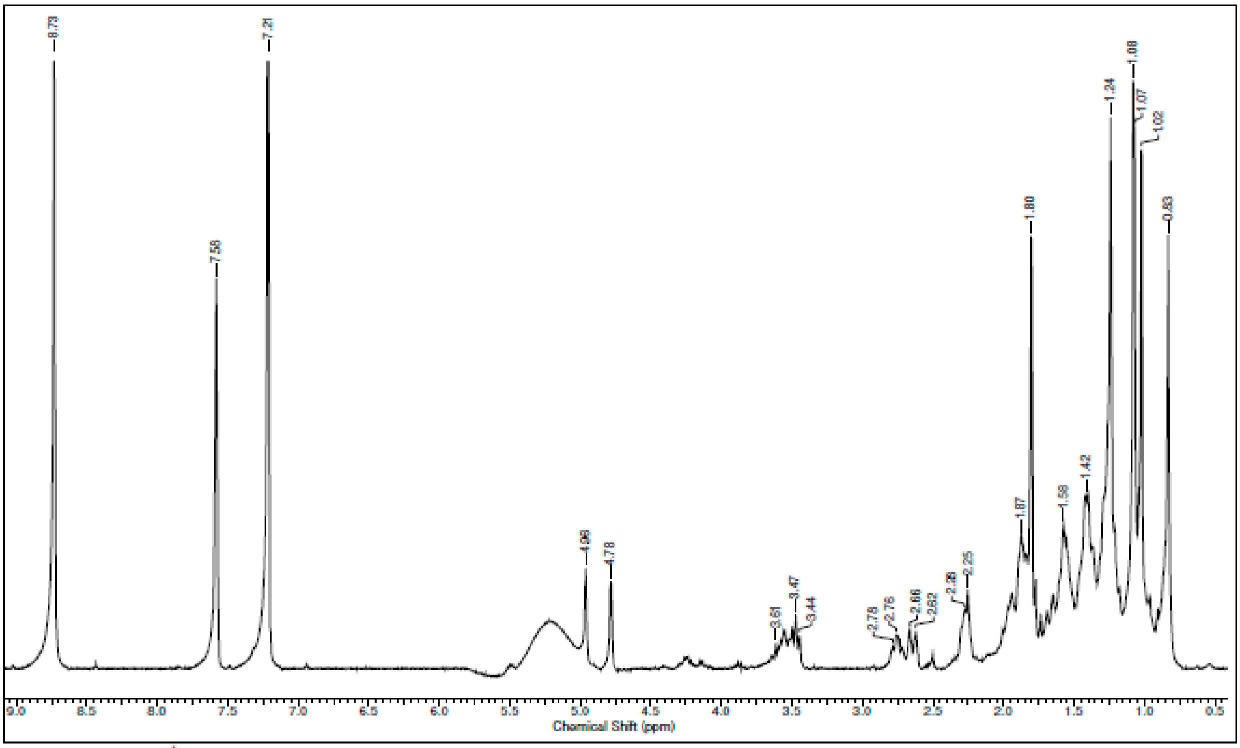

Figure S77. ^{1}H NMR spectrum (300 MHz, pyridine-d_5) of compound **14**.

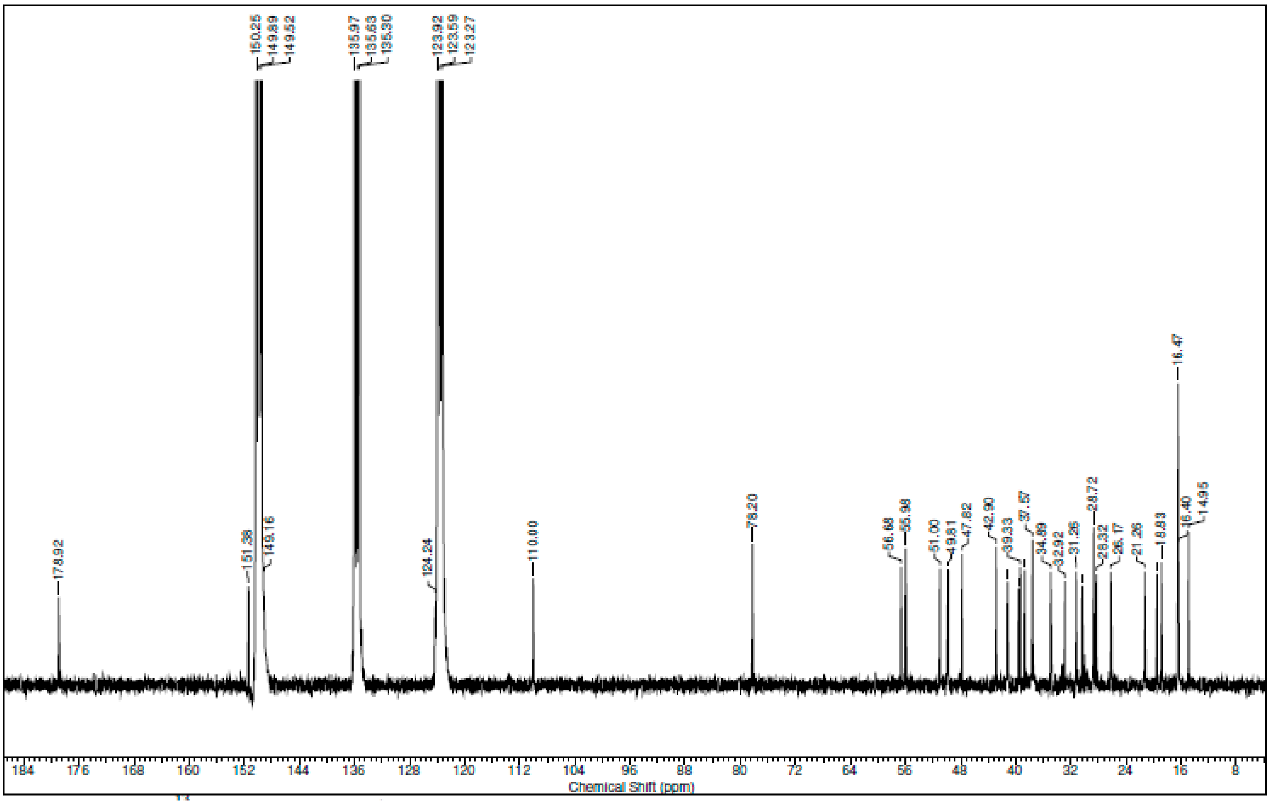

Figure S78. ^{13}C NMR spectrum (75 MHz, pyridine-d_5) of compound **14**.

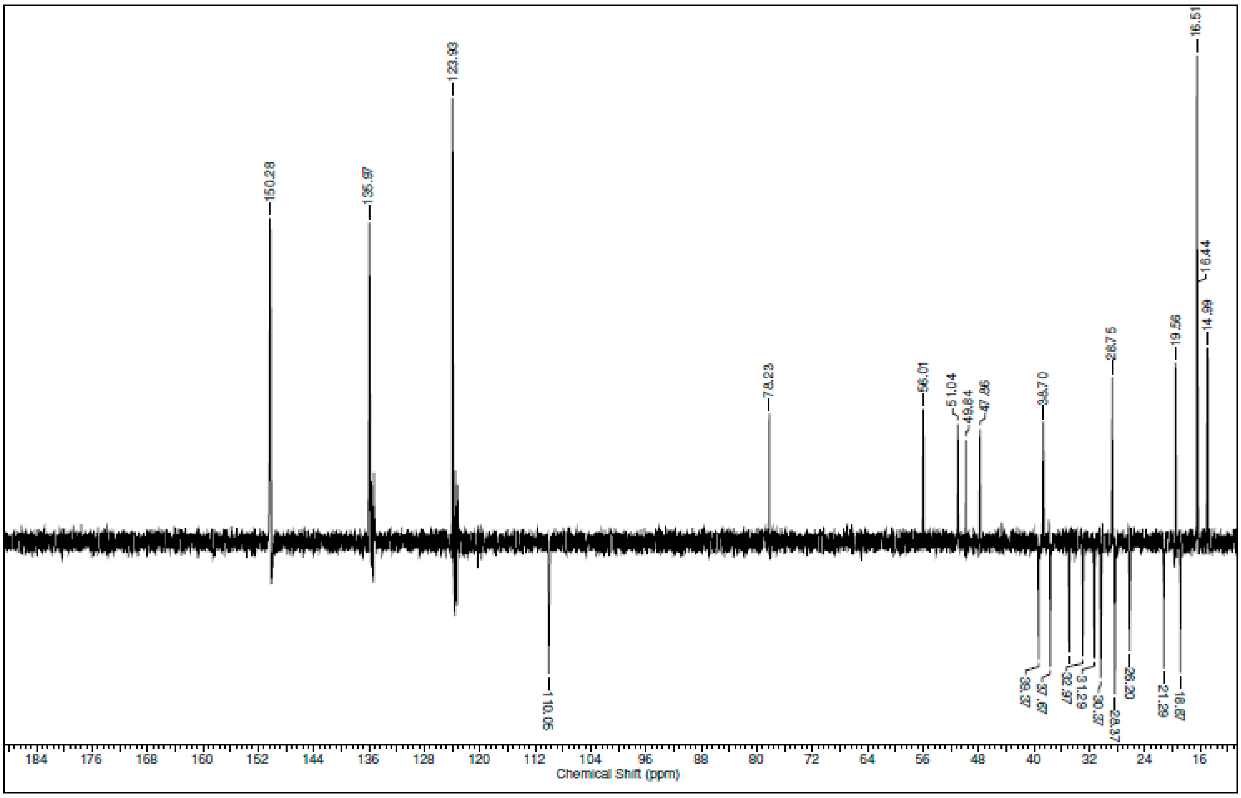

Figure S79. DEPT-135 spectrum (pyridine-d5) of compound **14**.

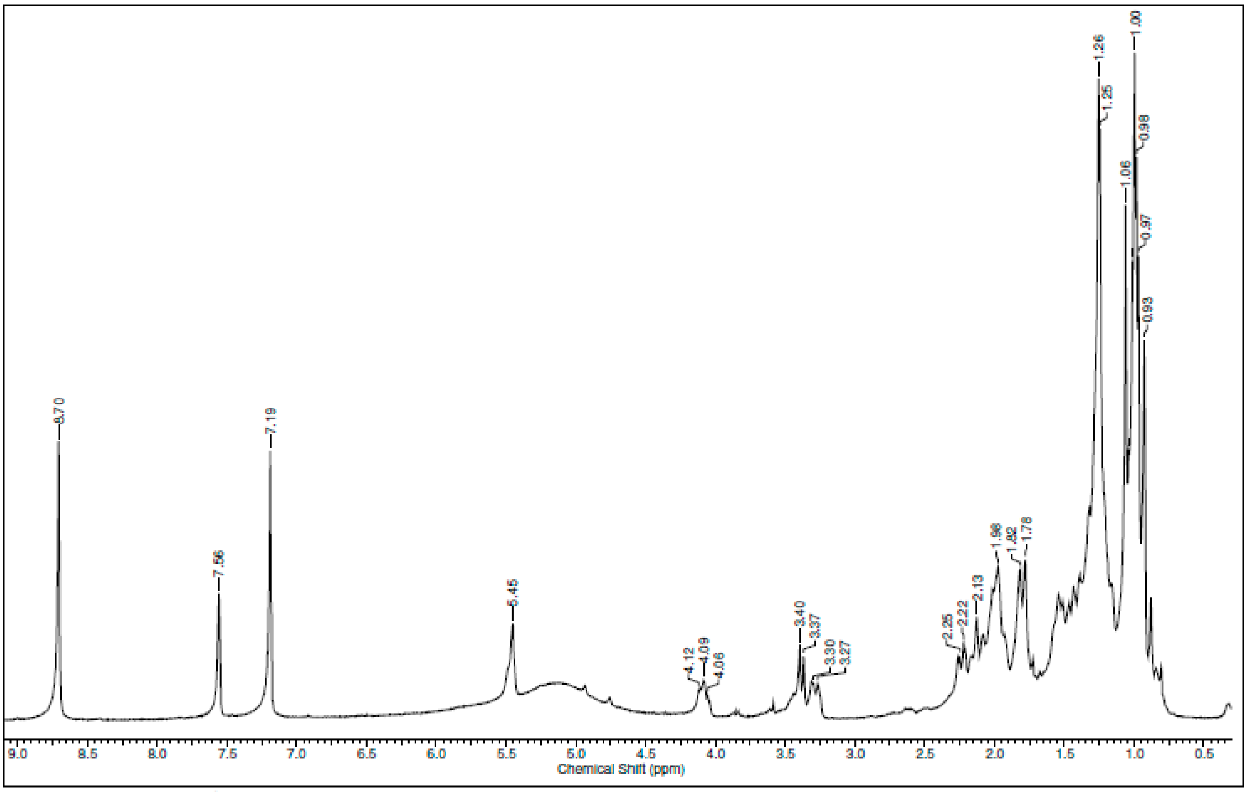

Figure S80. ^{1}H NMR spectrum (300 MHz, pyridine-d5) of compound **15**.

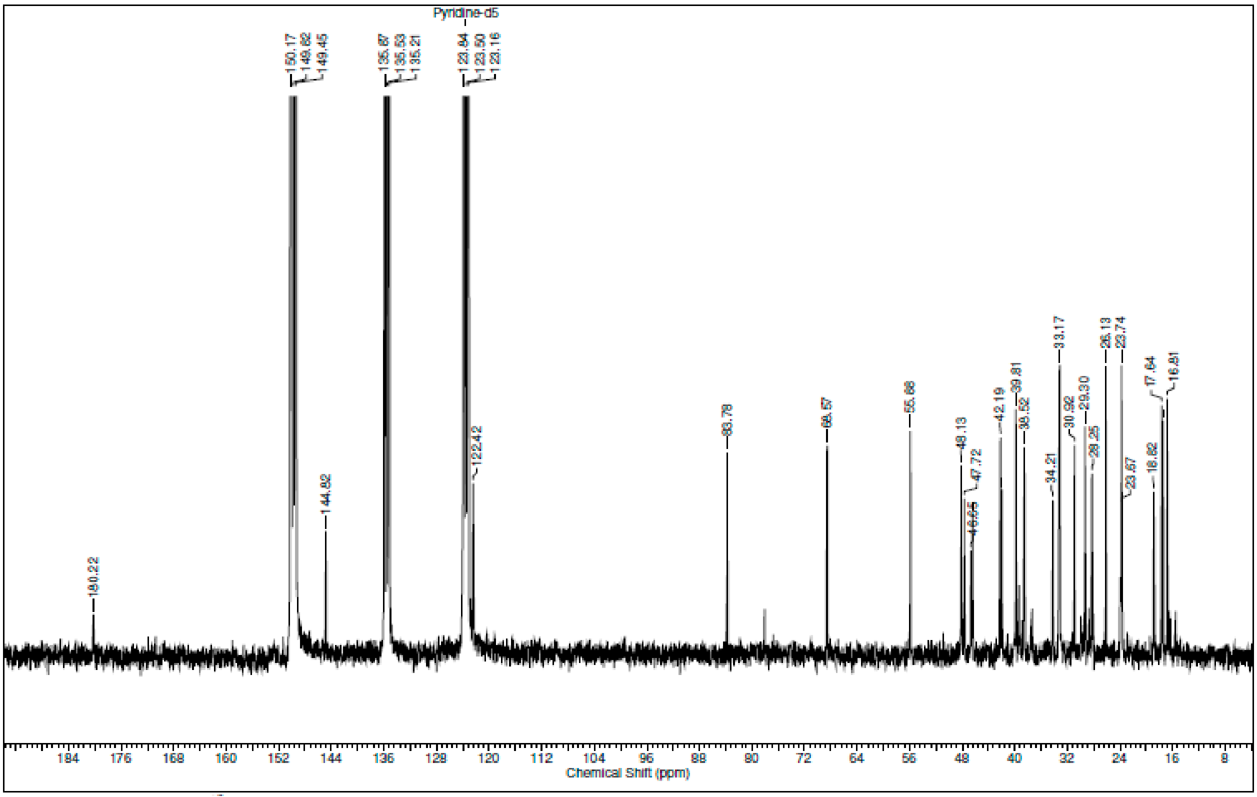

Figure S81. ^{13}C NMR spectrum (75 MHz, pyridine-d_5) of compound **15**.

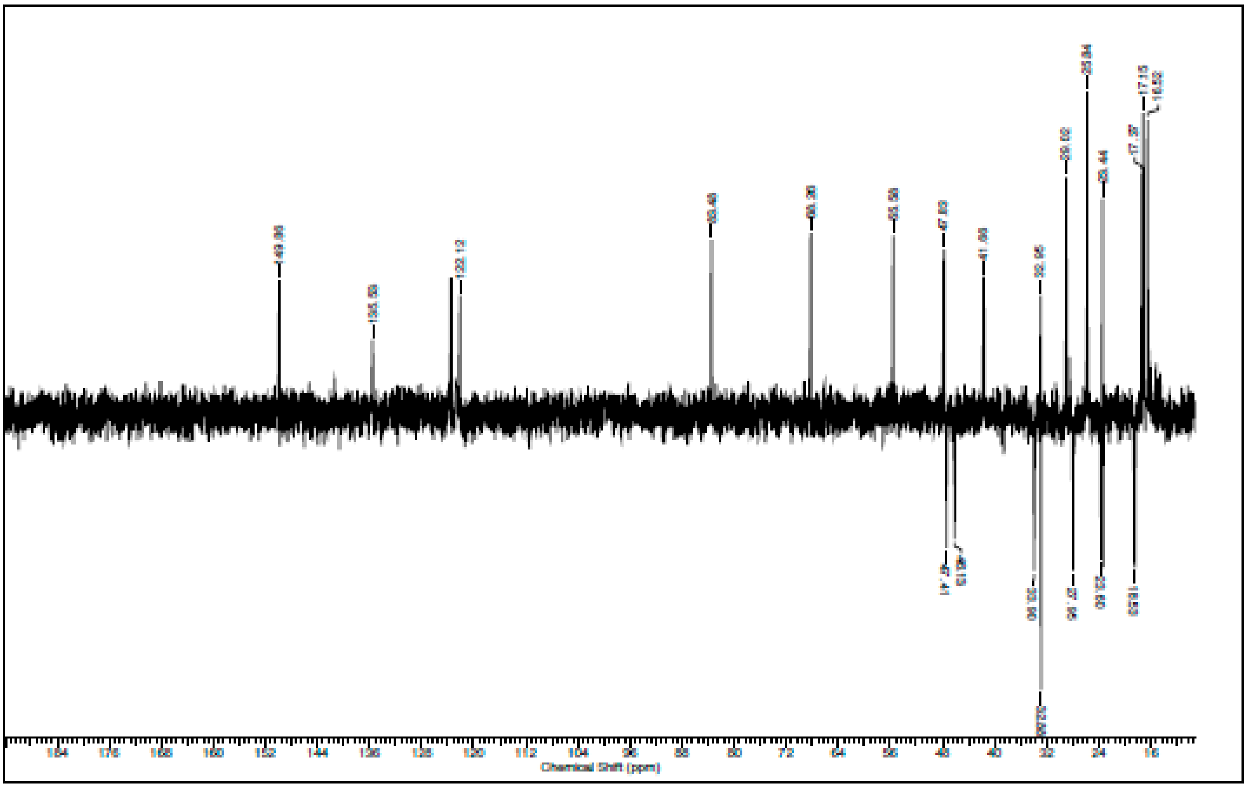

Figure S82. DEPT-135 spectrum (pyridine-d_5) of compound **15**.
